# Understanding the Influence of Donor‐Acceptor Diazo Compounds on the Catalyst Efficiency of B(C_6_F_5_)_3_ Towards Carbene Formation

**DOI:** 10.1002/chem.202104376

**Published:** 2022-01-27

**Authors:** Rasool Babaahmadi, Ayan Dasgupta, Christopher J. T. Hyland, Brian F. Yates, Rebecca L. Melen, Alireza Ariafard

**Affiliations:** ^1^ School of Natural Sciences (Chemistry) University of Tasmania Private Bag 75 Hobart Tasmania 7001 Australia; ^2^ Cardiff Catalysis Institute School of Chemistry Cardiff University Main Building, Park Place Cardiff CF10 3AT, Cymru/Wales UK; ^3^ School of Chemistry and Molecular Bioscience Molecular Horizons Research Institute University of Wollongong Wollongong New South Wales 2522 Australia

**Keywords:** carbene, density functional theory (DFT), diazo compounds, Lewis acid catalysis, mechanistic study, tris(pentafluorophenyl)borane

## Abstract

Diazo compounds have been largely used as carbene precursors for carbene transfer reactions in a variety of functionalization reactions. However, the ease of carbene generation from the corresponding diazo compounds depends upon the electron donating/withdrawing substituents either side of the diazo functionality. These groups strongly impact the ease of N_2_ release. Recently, tris(pentafluorophenyl)borane [B(C_6_F_5_)_3_] has been shown to be an alternative transition metal‐free catalyst for carbene transfer reactions. Herein, a density functional theory (DFT) study on the generation of carbene species from α‐aryl α‐diazocarbonyl compounds using catalytic amounts of B(C_6_F_5_)_3_ is reported. The significant finding is that the efficiency of the catalyst depends directly on the nature of the substituents on both the aryl ring and the carbonyl group of the substrate. In some cases, the boron catalyst has negligible effect on the ease of the carbene formation, while in other cases there is a dramatic reduction in the activation energy of the reaction. This direct dependence is not commonly observed in catalysis and this finding opens the way for intelligent design of this and other similar catalytic reactions.

## Graphical abstract

Extensive DFT calculations were carried out to interpret electronic effects of the substituents attached (electron donating/withdrawing substituents) to either side of a diazo functionality towards the ease of carbene generation from the corresponding diazo compounds in presence or absence of catalytic amounts of tris(pentafluorophenyl)borane [B(C_6_F_5_)_3_].

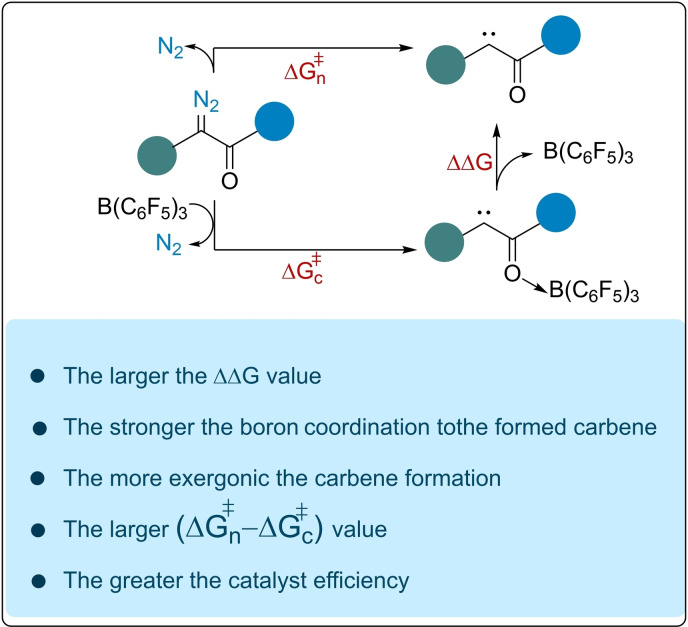

## Introduction

Diazo compounds have been extensively used as carbene precursors and have been employed as reagents for a range of functionalization reactions of organic molecules.^[1]^ The use of a precious transition metal catalyst is typically required for the generation of a metal carbenoid species.^[2]^ However, metal‐free approaches towards carbene generation are highly desirable for drug discovery where trace amounts of metal impurities in the target molecules can cause an issue.^[3]^ Recent studies have demonstrated that boranes can be used as an alternative to several transition metal catalysts to activate diazo compounds.^[4]^ In this regard, borane catalyzed carbene transfer has been employed for a range of reactions such as C−H,^[5]^ N−H,^[6]^ O−H^[7]^ insertion, C−C functionalization,^[8]^ carbocycle formation,[^5a^, ^9^] and ring opening reactions.^[5a]^ The efficacy of borane catalysts for carbene transfer reactions has raised our curiosity towards interpreting the mechanism for diazo‐activation. Many of the reports to date have included theoretical studies in which the mechanism for the borane catalyzed carbene transfer reaction has been proposed.[^5a^, ^10^] However, to date, the reactivity pattern between the Lewis acidic boranes and different donor‐acceptor diazo compounds has not yet been established. Theoretical calculations can provide a clear understanding on the stabilities and reactivities of diazo compounds, and their ease of carbene formation.^[11]^ With B(C_6_F_5_)_3_, initial DFT studies by Stephan et al. demonstrated an interaction between the Lewis acidic borane and diphenyldiazomethane^[12]^ which led to the formation of a reactive diazo‐borane adduct. Decomposition of the Ph_2_CN_2_ ⋅ B(C_6_F_5_)_3_ adduct subsequently led to the formation of a proposed carbene‐borane adduct [Ph_2_C ⋅ B(C_6_F_5_)_3_], which is exergonic by about 53 kcal/mol. In 2017, Wu et al. executed a theoretical calculation to rationalize the mode of activation of α‐aryl α‐diazocarbonyls using catalytic amounts of a Lewis acidic borane.^[10]^

**Scheme 1:**
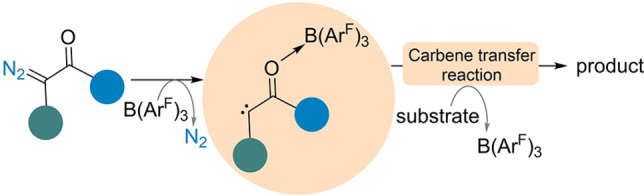
General mechanism for B(Ar^F^)_3_‐catalyzed carbene transfer reaction.

DFT calculations showed that B(C_6_F_5_)_3_ can bind with the α‐aryl α‐diazocarbonyl compound in three possible ways; as a B−N adduct, a B−C adduct, or through B−O adduct formation. The results showed that boron preferred to bind to the carbonyl functionality, promoting N_2_ release (Scheme 1). The resulting carbene is generated as the B−O adduct, exists as a conjugated system enhancing the electrophilic character of the carbene carbon due to the electron‐withdrawing effect of B(C_6_F_5_)_3_. However, the formation of the B−O adduct, barrier to N_2_ release, and the electrophilicity of the carbene center can also be directly influenced by the substituents attached to the either side of the aryl ring or carbonyl functionality. Indeed, in our previous studies, we have observed differing rates of reactivity when using different diazo compounds.[^5a^, ^5b^, ^5c^]

Therefore, we decided to undertake a DFT study at the SMD/M06‐2X/def2‐TZVP//SMD/M06‐2X/6‐31G(d) level of theory in dichloromethane (see Supporting Information) to establish the energy barriers for B(C_6_F_5_)_3_‐catalyzed carbene formation from the corresponding α‐aryl α‐diazocarbonyl compounds when varying the electronic effects on the diazo compound. The primary focus of this research is on the specific influence of substituents on diazo substrates on catalyst efficiency, rather than on the mechanism of the reaction, which has already been established.[^5a^, ^10^]

## Results and Discussion

**Scheme 2:**
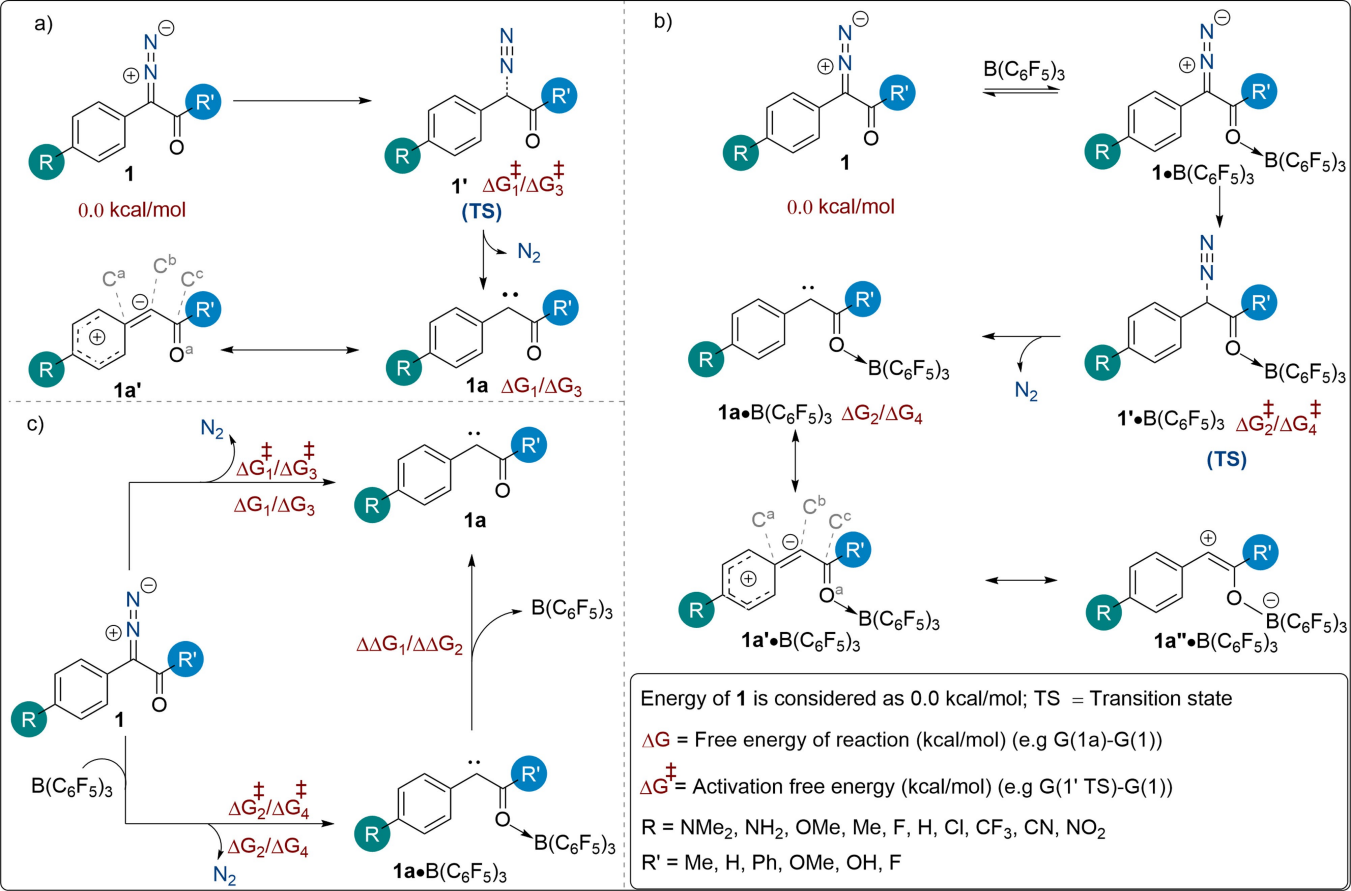
a) Carbene generation without using B(C_6_F_5_)_3_; b) Carbene generation in presence of B(C_6_F_5_)_3_; and c) Generic representation for carbene formation and the free energies associated with each step using catalytic B(C_6_F_5_)_3_.

We began our investigations with an examination of how readily a free carbene can be generated from an α‐aryl α‐diazocarbonyl precursor in the absence of the boron Lewis acid catalyst (Scheme 2a). Different R substitutions on the aryl ring were introduced when R′=OMe and their influence on the generation of a free carbene was calculated (Table 1). As shown in Table 1, the reaction free energy for carbene formation (ΔG_1_) was found to be highly dependent upon the electronic nature of the R group. An upward shift in the ΔG_1_ value was observed going from electron donating groups (NMe_2_, NH_2_, OMe, and Me; Table 1; entries 1–4) to electron withdrawing groups (CF_3_, CN, NO_2_; Table 1; entries 8–10).

**Table 1:** Calculated free energies for the uncatalyzed and B(C_6_F_5_)_3_ catalyzed carbene formation from α‐aryl α‐diazocarbonyl with different R groups. Free energies are given in kcal/mol. The distance (r_C_^a^_–C_^b^) and WBI for the C^a^−C^b^ bond in **1 a** (Scheme 2a). The B−O bond distance (r_B_−_O_) in **1 a** ⋅ B(C_6_F_5_)_3_. Hammett σ_p _values for different R groups

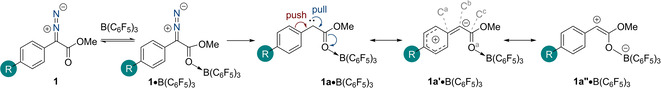											
Entry	R	ΔG^≠^_1_	ΔG_1_	r_C_^a^−_C_^b^ [Å]	σ_p_	WBI C^a^−C^b^	ΔG^≠^_2_	ΔG_2_	r_B−O_ [Å]	ΔΔG^≠^_1_=ΔG^≠^_1_−ΔG^≠^_2_	ΔΔG_1_=ΔG_1_−ΔG_2_
		*Uncatalyzed*					*B(C_6_F_5_)_3_ catalyzed*			*Catalyst efficiency* ^[x]^	
1	NMe_2_	26.7	−0.1	1.388	−0.83	1.460	15.4	−13.8	1.516	11.3	13.7
2	NH_2_	26.8	0.3	1.389	−0.66	1.457	16.5	−11.9	1.517	10.3	12.2
3	OMe	29.1	6.6	1.403	−0.27	1.369	21.1	−1.9	1.519	8.0	8.5
4	Me	30.7	11.2	1.415	−0.17	1.310	25.1	5.9	1.539	5.6	5.3
5	F	31.1	11.3	1.415	0.06	1.305	26.3	6.1	1.529	4.8	5.2
6	H	32.0	13.1	1.421	0.0	1.282	25.9	6.5	1.533	6.1	6.6
7	Cl	32.1	13.8	1.421	0.23	1.277	27.7	9.8	1.546	4.4	4.0
8	CF_3_	33.0	16.1	1.430	0.54	1.243	30.5	14.2	1.542	2.5	1.9
9	CN	34.6	18.3	1.432	0.66	1.233	32.2	14.9	1.548	2.4	3.4
10	NO_2_	35.1	19.1	1.435	0.78	1.219	34.3	16.7	1.551	0.8	2.4

[x] Ability of the catalyst to reduce the activation free energies. The large values for ΔΔG^≠^_1_/ΔΔG_1_ indicate a high efficiency of the catalyst while the small values indicate a low efficiency of the catalyst; All the σ_p _values for different R groups reported in the Table 1 were taken from the literature report (C. Hansch, A. Leo, R. W. Taft, *Chem. Rev*. **1991**, *91*, 165–195).

**Figure 1:**
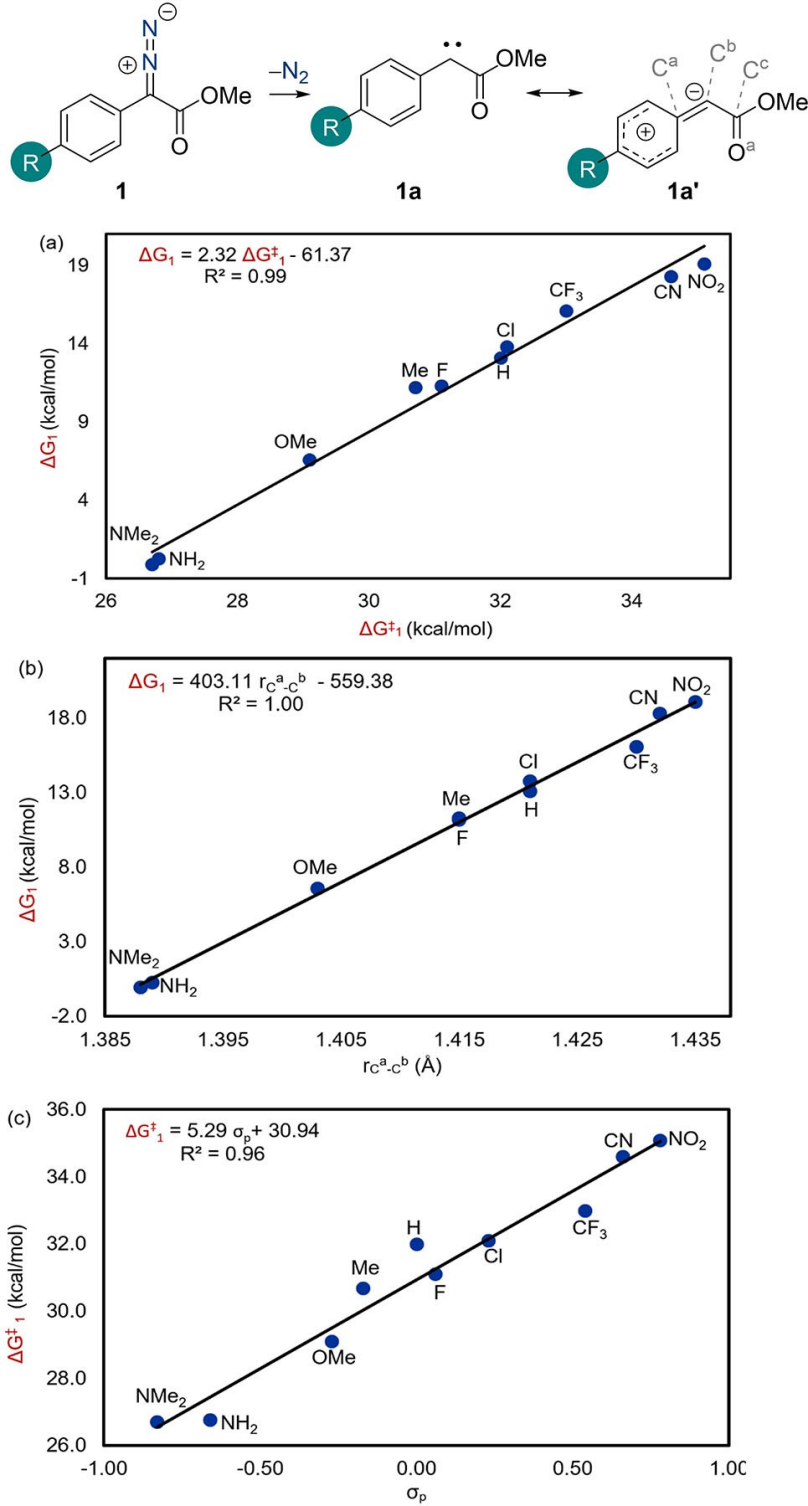
Correlation plots for uncatalyzed carbene formation. a) ΔG^≠^_1_ versus ΔG_1_; b) ΔG_1_ versus r_C_^a^−_C_^b^, and c) ΔG^≠^_1_ versus σ_p_.

The same trend was observed with the activation barrier for the reaction (ΔG^≠^_1_) which increased when moving from electron donating groups (NMe_2_: 26.7 kcal/mol) to electron withdrawing groups (NO_2_: 35.1 kcal/mol). These results indicate that the stability of the formed carbene determines the activation barrier for the reaction. Thus, more stabilized carbenes are formed through a lower energy transition structure. Consistent with Hammond's postulate,^[13]^ the more thermodynamically favorable the carbene formation, the lower the barrier to the N_2_ release. This finding is supported by the strong correlation between ΔG^≠^_1_ and ΔG_1_ with an R^2^ (the squared correlation coefficient) value of 0.99 (Figure 1a). The stability of the formed carbene **1 a** bearing an electron donating group (NMe_2_/NH_2_/OMe) is a result of the π‐donation from the aromatic ring to the empty p orbital on the C^b^ atom and is enhanced by increasing the contribution of resonance structure **1 a′** (Scheme 2a).

The degree of this stabilization can be ascertained through analysis of the C^a^−C^b^ bond lengths in **1 a**, with shorter bond lengths indicative of increased stabilization of the carbene. This was supported by a strong correlation between the C^a^−C^b^ bond length in **1 a** and the Gibbs free energy for carbene formation (ΔG_1_) (Table 1, Figure 1b). Shorter bond distances are observed when R is a π‐donating group, implying that the π‐bond character between the C^a^ and C^b^ atoms is increased, and hence structure **1 a′** has a greater contribution (Scheme 2a) This is reiterated by the Wiberg bond indices (WBI) analysis,^[14]^ which showed the largest WBI value for R=NMe_2_ (1.460) and the smallest for R=NO_2_ (1.219, Table 1).

The importance of the electronic nature of the R group with respect to the carbene stabilization is evidenced by the direct correlation between the Hammett σ_p_ value and the activation barrier to N_2_ release (ΔG^≠^_1_) (Figure 1c).

We next turned our attention to understand the effect of the B(C_6_F_5_)_3_ catalyst on the activation energy for N_2_ release from the α‐aryl α‐diazocarbonyl compound (Scheme 2b). The binding of the Lewis acidic borane to the carbonyl functionality (O→B adduct) facilitates this process both kinetically and thermodynamically (Table 1).^[5a]^ However, this facilitating effect is primarily dependent on the identity of the R group. The magnitude of ΔΔG^≠^_1_ determines the extent of this effect on the kinetic property (Table 1); ΔΔG^≠^_1_ is the difference in the activation energies between the uncatalyzed and catalyzed reactions, i. e., ΔG^≠^_1_‐ΔG^≠^_2_. Accordingly, the maximum effect is found for the systems bearing an electron‐donating R group and the minimum effect for the systems where the R group is highly electron withdrawing. This means that the nature of the R group influences the efficiency of the catalyst. For example, for R=NMe_2_ with ΔΔG^≠^_1_=11.3 kcal/mol (Table 1, entry 1), the activation barrier (ΔG^≠^_2_) for the catalyzed N_2_ release is calculated to be 15.4 kcal/mol, whereas the uncatalyzed reaction using the same functional group requires an activation energy of 26.7 kcal/mol (ΔG^≠^_1_). No significant energy difference was observed for electron‐withdrawing substituents such as the NO_2_ functionality; here, ΔΔG^≠^_1_ was found to be only 0.8 kcal/mol (Table 1, entry 10). These findings are consistent with our previous experimental study,^[15]^ in which we clearly demonstrated that the diazo compound bearing a strong electron‐withdrawing substituent (CF_3_) required a strong Lewis acidic borane^[16]^ and high temperature to generate the corresponding carbene intermediate.

**Figure 2:**
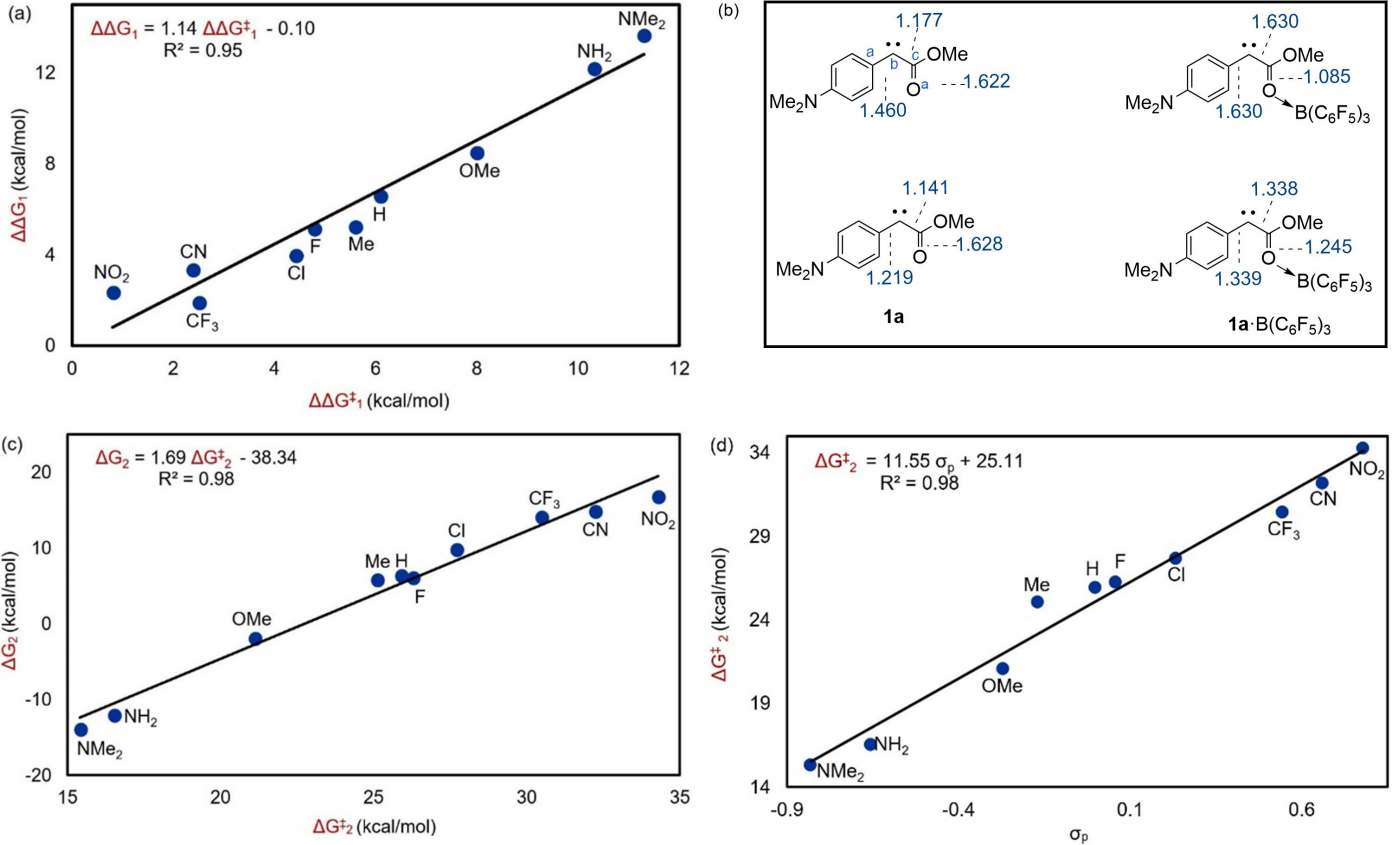
Correlation plots for B(C_6_F_5_)_3_ catalyzed carbene formation. a) ΔΔG_1_ versus ΔΔG^≠^_1_; b) WBI for **1 a** and **1 a** ⋅ B(C_6_F_5_)_3_ with R=NMe_2_ and NO_2_; c) ΔG^≠^_2_ versus ΔG_2_, and d) ΔG^≠^_2_ versus σ_p_.

Our DFT studies disclosed the same trend for ΔΔG_1_ which reveals the difference between the free energies of the uncatalyzed and catalyzed reactions (ΔG_1_‐ΔG_2_, Table 1, Scheme 2c). The magnitude of ΔΔG_1_ indicates how strongly the borane catalyst binds to the carbene species (**1 a**). Our results showed that the diazo compounds bearing a strongly electron donating R group exhibit strong coordination between the borane catalyst and the formed carbene. Likewise, a weak coordination is observed for diazo compounds bearing electron withdrawing R groups. For example, when R=NMe_2_, ΔΔG_1_ is found to be 13.7 kcal/mol, but when R is NO_2_, ΔΔG_1_ is only 2.4 kcal/mol (Table 1). Interestingly, we found a strong positive correlation between ΔΔG_1_ and ΔΔG^≠^_1_ with R^2^=0.95 (Figure 2a). This strong correlation indicates that the strength of the O−B bond in carbene **1 a** ⋅ B(C_6_F_5_)_3_ governs catalyst efficiency; the more electron donating the R group, the stronger the borane coordination to the carbene, the greater the ΔΔG^≠^_1_ value, and the more efficient the catalyst. This finding can serve as a guideline for determining how to alter the R group in a diazo compound in order to maximize catalyst efficiency.^[16]^

In line with the above discussion, an electron donating R group is expected to increase the contribution of the resonance structure **1 a′** ⋅ B(C_6_F_5_)_3_ (Table 1) which increases the electron density on the C^b^ atom. The borane bonded to the carbonyl group in **1 a** ⋅ B(C_6_F_5_)_3_ receives some of this electron density by involving resonance structure **1 a′′** ⋅ B(C_6_F_5_)_3_. Owing to the existence of a kind of push‐pull interaction in **1 a** ⋅ B(C_6_F_5_)_3_ (Table 1), the contribution of these two resonance structures is expected to be increased by increasing the electron donating ability of the R group. An increase in the contribution of these two resonance structures raises the π‐bond character between the C^a^ and C^b^ atoms and between the C^b^ and C^c^ atoms while decreasing the π‐bond character between the C^c^ and O^a^ atoms. This claim is supported by the WBI analysis for **1 a** and **1 a** ⋅ B(C_6_F_5_)_3_ with R=NO_2_ and NMe_2_ (Figure 2b). Accordingly, **1 a** ⋅ B(C_6_F_5_)_3_ with R=NMe_2_ has the highest WBI values for the C^a^−C^b^ and C^b^−C^c^ bonds and the lowest one for the C^c^−O bond, supporting the π‐bond character argument.

An increase in the contribution of these two resonance structures causes the boron catalyst to bind more strongly to the carbene, corroborated by the shortening of the B−O bond distance in **1 a** ⋅ B(C_6_F_5_)_3_ upon moving from R=NO_2_ to NMe_2_ (Table 1).

Figure 2c shows a plot with strong correlation between ΔG_2_ and ΔG^≠^_2_ (R^2^=0.98). This means that regardless of whether the borane is involved in the N_2_ release process, the thermodynamic favourability of carbene formation influences its activation barrier.^[17]^ The formula in Figure 2d, which is based on the strong correlation between the Hammett σ_p_ value and ΔG^≠^_2_, will allow researchers to estimate the barrier to N_2_ release from a specific α‐diazo‐carbonyl compound using the σ_p_ value.

Next, attention was turned to investigating the effect of changing the R′ substituent on the N_2_ release process while keeping R=H (Table 2). Our calculations revealed that in the absence of the boron‐catalyst, changing R′ plays no significant role in the ease of the N_2_ release process compared to changing R. This can be attributed to the inability of this group to stabilize the formed carbene. Specifically, the activation free energy (ΔG^≠^_3_) for N_2_ release occurs over a very narrow range of ∼30–32 kcal/mol for all R′ groups. The same is true for the reaction energy (ΔG_3_, Table 2, Scheme 2a).

**Table 2:** Calculated free energies for uncatalyzed carbene formation with different R′ groups, free energy difference between B(C_6_F_5_)_3_ catalyzed and uncatalyzed carbene formation, and free energy values for B(C_6_F_5_)_3_ catalyzed carbene formation. Free energies are given in kcal/mol. The B−O bond distance (r_B−O_) in **1 a** ⋅ B(C_6_F_5_)_3_ .

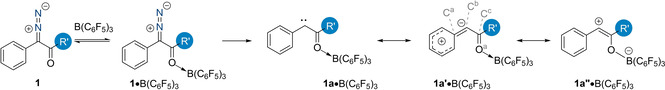								
Entry	R′	ΔG^≠^_3_	ΔG_3_	ΔG^≠^_4_	ΔG_4_	r_B−O_ [Å]	ΔΔG^≠^_2_=ΔG^≠^_3_–ΔG^≠^_4_	ΔΔG_2_=ΔG_3_–ΔG_4_
		*Uncatalyzed*		*B(C_6_F_5_)_3_ catalyzed*			*Catalyst efficiency* ^[a]^	
1	Me	30.4	10.4	9.5	−6.4	1.509	20.9	16.8
2	H	31.3	13.4	10.5	−6.8	1.512	20.8	20.2
3	Ph	31.2	9.3	11.4	−6.1	1.518	19.8	15.4
4	OMe	32.0	13.1	25.9	6.5	1.533	6.1	6.6
5	OH	30.6	12.1	26.1	7.8	1.534	4.5	4.3
6	F	30.1	11.1	27.3	9.0	1.543	2.8	2.1

[a] Ability of the catalyst to reduce the activation free energies. The large values for ΔΔG^≠^_2_/ΔΔG_2_ indicate a high efficiency of the catalyst while the small values indicate a low efficiency of the catalyst.

Finally, attention was turned towards the influence of R′ on the ease of the N_2_ release process in the presence of the boron‐catalyst, while keeping R=H (Table 2, Scheme 2b, ΔG_4_ and ΔG^≠^_4_). Interestingly, in this case, the electronic nature of R′ has a profound impact on the N_2_ release process, with R′ substituents lacking a lone pair (R′=Me, H and Ph) favoring this process.

**Figure 3:**
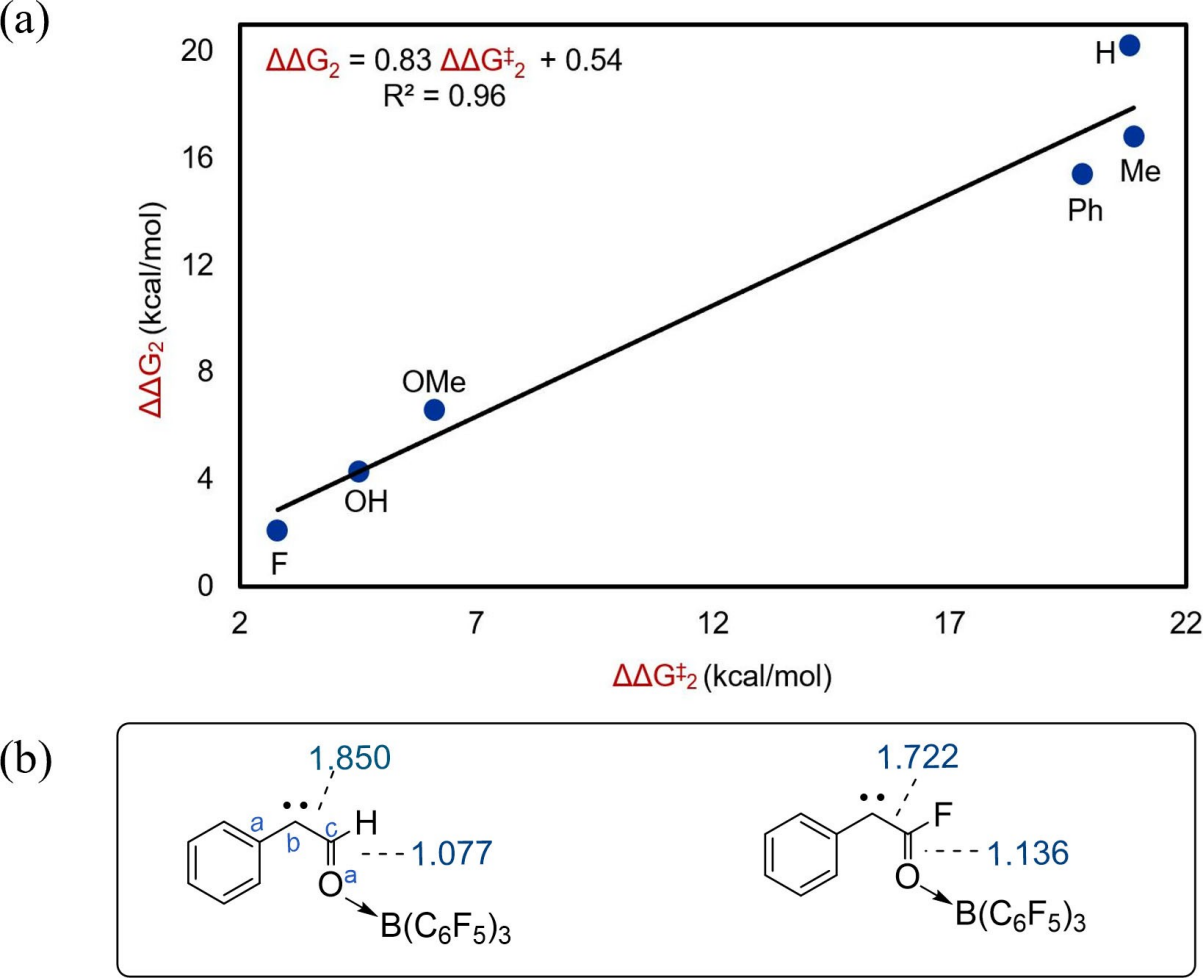
B(C_6_F_5_)_3_ catalyzed correlation plots for carbene formation with different R′ groups. a) ΔΔG^≠^_2_ versus ΔΔG_2_, and b) WBI for **1 a** ⋅ B(C_6_F_5_)_3_ with R′=H and F.

Again, we discovered that the activation barrier to the N_2_ liberation can be determined by how strongly the borane binds to the carbene in **1 a** ⋅ B(C_6_F_5_)_3_. A plot between ΔΔG_2_ and ΔΔG^≠^_2_ was found to be a straight line with R^2^=0.96 (Figure 3a) where ΔΔG_2_=ΔG_3_−ΔG_4_ and ΔΔG^≠^_2_=ΔG^≠^_3_−ΔG^≠^_4_ (Scheme 2c).

This result once again demonstrates that the stronger the borane coordination to the formed carbene, the lower the activation barrier to N_2_ liberation, the greater the efficiency of the catalyst.

The resonance contributor **1 a’′** ⋅ B(C_6_F_5_)_3_ (Table 2) is favored when R′=Me, H, Ph, whereas lone pair bearing donor R′ groups, such as OMe, OH, and F, significantly reduce this contribution. Indeed, the presence of a lone pair on R′ does not allow the carbene lone pair to effectively interact with the carbonyl functional group, resulting in the contribution of **1 a′′** ⋅ B(C_6_F_5_)_3_ being decreased.

The above assumption is supported by a comparison of the WBI values for **1 a** ⋅ B(C_6_F_5_)_3_ with R′=H and F (Figure 3b). The WBI for the bond between the C^b^ and C^c^ atoms is greater for R′=H than for R′=F, whereas WBI values between the C^c^ and O^a^ atoms has the opposite order. This indicates that, for R′=H, the π‐bond character between the C^b^ and C^c^ atoms is greater, while a reverse order is observed for the bond between the C^c^ and O^a^ atoms. This analysis demonstrates a greater contribution of **1 a′′** ⋅ B(C_6_F_5_)_3_ for the carbene with R′=H. The greater contribution of **1 a′′** ⋅ B(C_6_F_5_)_3_ leads to a stronger coordination of the borane to carbene, evidenced by a shorter B−O bond distance for the carbene with R′=Me, H and Ph (Table 2). We also found an excellent correlation between the ΔG_4_ and ΔG^≠^_4_ with R^2^=0.99, reconfirming the strong relationship between the stability of the formed carbene and the ease of N_2_ liberation (Figure S2).^[18]^

## Conclusion

In conclusion, we have demonstrated computationally the influence of different substituents on the aryl ring and carbonyl functionality of α‐aryl α‐diazocarbonyls towards the formation of carbene species in the presence and absence of a B(C_6_F_5_)_3_ catalyst. We have several important findings, which are detailed below. Based on these DFT calculations, we conclude that there is a strong correlation between the activation barrier to the N_2_ liberation and the reaction free energy of the carbene formation. In general, the more stable the carbene generated, the lower the activation barrier to the process. In the absence of any Lewis acidic borane catalyst, a change in the electronic nature of the R group attached to the aryl ring has a considerable impact upon the ease of the N_2_ release, whilst the identity of the R′ group is unimportant. The activation barrier is reduced as the π‐donor ability of the R group is enhanced. This is because the carbene becomes more stable as the π‐donor ability of the R group increases. The most important finding from this study is that when the borane catalyst is present, both the R and R′ substituents have a direct influence on the stability of the carbene and, as a result, on the efficiency of the catalyst. The catalyst efficiency is determined by how strongly the borane is bonded to the formed carbene and we found that it is the greatest if the R group is a strong π‐donor and the R′ group is a weak π‐donor. In other words, the nature of the diazo substrate affects the catalyst efficiency. In short, the stronger the coordination of the borane to the generated carbene, the more thermodynamically favorable is the carbene formation, the lower the activation barrier to the N_2_ liberation, and the more efficient the catalyst. These computational calculations will aid the further exploration of B(C_6_F_5_)_3_ as a catalyst for carbene transfer reactions and will help to understand both the reactivity of diazo substrates with other nucleophiles as well as the relative rates of reaction.

## Experimental Section

Gaussian 16^[19]^ was used to fully optimize all the structures at the M06‐2X level^[20]^ of theory using the SMD solvation model^[21]^ in dichloromethane. The 6‐31G(d) basis set^[22]^ was chosen for all atoms. Frequency calculations were carried out at the same level of theory as those for the structural optimization. Transition structures were located using the Berny algorithm and intrinsic reaction coordinate (IRC) calculations^[23]^ were employed to confirm the connectivity between transition structures and minima.

To further refine the energies obtained from the SMD/M06‐2X/6‐31G(d) calculations, single‐point energy calculations using the M06‐2X functional method were carried out for all of the structures with a larger basis set def2‐TZVP^[24]^ and the SMD solvation model in dichloromethane. All thermodynamic data were calculated in the standard state (298.15 K and 1 atm). An additional correction for compression of 1 mol of an ideal gas from 1 atm to the 1 M solution phase standard state (1.89 kcal/mol) was applied.^[25]^ Wiberg Bond Index (WBI) calculations were determined by the NBO6 program.^[26]^

## Conflict of interest

The authors declare no conflict of interest.

1

## Supporting information

As a service to our authors and readers, this journal provides supporting information supplied by the authors. Such materials are peer reviewed and may be re‐organized for online delivery, but are not copy‐edited or typeset. Technical support issues arising from supporting information (other than missing files) should be addressed to the authors.

Supporting Information

## Data Availability

The data that support the findings of this study are available in the supplementary material of this article.

## References

[1] For selected reviews see:

[1a] D. Hu , L. Chen , H. Fan , Q. Yao , S. Zhu , Chem. Soc. Rev. 2020, 49, 908 **–** 950; 31958107 10.1039/c9cs00542k

[1b] Y. Xiang , C. Wang , Q. Ding , Y. Peng , Adv. Synth. Catal. 2019, 361, 919 **–** 944;

[1c] Ł. W. Ciszewski , K. Rybicka-Jasińska , D. Gryko , Org. Biomol. Chem. 2019, 17, 432 **–** 448; 30543264 10.1039/c8ob02703j

[1d] Y. Xia , D. Qiu , J. Wang , Chem. Rev. 2017, 117, 13810 **–** 13889; 29091413 10.1021/acs.chemrev.7b00382

[1e] K. A. Mix , M. R. Aronoff , R. T. Raines , ACS Chem. Biol. 2016, 11, 3233 **–** 3244. 27739661 10.1021/acschembio.6b00810 PMC5161546

[2] For selected reviews see:

[2a] H. M. L. Davies , K. Liao , Nat. Chem. Rev. 2019, 3, 347 **–** 360; 10.1038/s41570-019-0099-x PMC7521778 32995499

[2b] D. Gillingham , N. Fei , Chem. Soc. Rev. 2013, 42, 4918 **–** 4931; 23407887 10.1039/c3cs35496b

[2c] H. M. L. Davies , J. R. Manning , Nature 2008, 451, 417 **–** 424. 18216847 10.1038/nature06485 PMC3033428

[3a] P. B. Tchounwou , C. G. Yedjou , A. K. Patlolla , D. J. Sutton , in: Molecular, Clinical and Environmental Toxicology, Volume 3: Environmental Toxicology (Ed.: A. Luch ), Springer Basel, Basel, 2012, pp. 133 **–**164;

[3b] K. S. Egorova , V. P. Ananikov , Organometallics 2017, 36, 4071 **–** 4090.

[4] For selected examples see:

[4a] A. Dasgupta , E. Richards , R L Melen , ACS Catal. 2022, 12, 442 **–** 452; 35028191 10.1021/acscatal.1c04746 PMC8749965

[4b] A. Dasgupta , S. Pahar , L. Gierlichs , R. Babaahmadi , B. F. Yates , A. Ariafard R L Melen , Adv. Synth. Catal. 2021, 10.1002/adsc.202101312; PMC8596400 34590773

[4c] S. Rao , P. K. S. Ashwathappa , K. R. Prabhu , Asian J. Org. Chem. 2019, 8, 320 **–** 323;

[4d] R. L. Melen , Angew. Chem. Int. Ed. 2018, 57, 880 **–** 882; 10.1002/anie.201711945 29219236

[4e] J. B. Geri , J. P. Shanahan , N. K. Szymczak , J. Am. Chem. Soc. 2017, 139, 5952 **–** 5956; 28414226 10.1021/jacs.7b01982 PMC5965694

[4f] A. Simonneau , R. Turrel , L. Vendier , M. Etienne , Angew. Chem. Int. Ed. 2017, 56, 12268 **–** 12272; 10.1002/anie.201706226 28766855

[5a] A. Dasgupta , R. Babaahmadi , B. Slater , B. F. Yates , A. Ariafard , R. L. Melen , Chem 2020, 6, 2364 **–** 2381;

[5b] A. Dasgupta , K. Stefkova , R. Babaahmadi , L. Gierlichs , A. Ariafard , R. L. Melen , Angew. Chem. Int. Ed. 2020, 59, 15492 **–** 15496; 10.1002/anie.202007176 PMC7497215 32485034

[5c] V. Nori , A. Dasgupta , R. Babaahmadi , A. Carlone , A. Ariafard , R. L. Melen , Catal. Sci. Technol. 2020, 10, 7523 **–** 7530;

[5d] H. H. San , C.-Y. Wang , H.-P. Zeng , S.-T. Fu , M. Jiang , X.-Y. Tang , J. Org. Chem. 2019, 84, 4478 **–** 4485; 30855950 10.1021/acs.joc.8b03278

[5e] H. H. San , S.-J. Wang , M. Jiang , X.-Y. Tang , Org. Lett. 2018, 20, 4672 **–** 4676. 30033730 10.1021/acs.orglett.8b01988

[6a] Y. Zhao , D. Mandal , J. Guo , Y. Wu , D. W. Stephan , Chem. Commun. 2021, 57, 7758 **–** 7761; 10.1039/d1cc03048e 34254070

[6b] F. He , R. M. Koenigs , Org. Lett. 2021, 23, 5831 **–** 5835. 34279967 10.1021/acs.orglett.1c01982

[7] Z. Yu , Y. Li , J. Shi , B. Ma , L. Liu , J. Zhang , Angew. Chem. Int. Ed. 2016, 55, 14807 **–** 14811; 10.1002/anie.201608937 27782348

[8] S. Rao , R. Kapanaiah , K. R. Prabhu , Adv. Synth. Catal. 2019, 361, 1301 **–** 1306.

[9a] K. Stefkova , M. J. Heard , A. Dasgupta , R. L. Melen , Chem. Commun. 2021, 57, 6736 **–** 6739; 10.1039/d1cc01856f 34132279

[9b] J. P. Mancinelli , S. M. Wilkerson-Hill , ACS Catal. 2020, 10, 11171 **–** 11176.

[10] Q. Zhang , X.-F. Zhang , M. Li , C. Li , J.-Q. Liu , Y.-Y. Jiang , X. Ji , L. Liu , Y.-C. Wu , J. Org. Chem. 2019, 84, 14508 **–** 14519. 31638807 10.1021/acs.joc.9b02035

[11] S. P. Green , K. M. Wheelhouse , A. D. Payne , J. P. Hallett , P. W. Miller , J. A. Bull , Org. Process Res. Dev. 2020, 24, 67 **–** 84. 31983869 10.1021/acs.oprd.9b00422 PMC6972035

[12] C. Tang , Q. Liang , A. R. Jupp , T. C. Johnstone , R. C. Neu , D. Song , S. Grimme , D. W. Stephan , Angew. Chem. Int. Ed. 2017, 56, 16588 **–** 16592; 10.1002/anie.201710337 29108092

[13] G. S. Hammond , J. Am. Chem. Soc. 1955, 77, 334 **–** 338.

[14] K. B. Wiberg , Tetrahedron 1968, 24, 1083 **–** 1096.

[15] M. Santi , D. M. C. Ould , J. Wenz , Y. Soltani , R. L. Melen , T. Wirth , Angew. Chem. Int. Ed. 2019, 58, 7861 **–** 7865; 10.1002/anie.201902985 30897253

[16] The identity of the borane catalyst may also have an impact on its efficiency. Our calculations indicate that the N_2_ release process is facilitated more effectively if a borane with a higher acidity is used (Table S2).

[17] The boron binds to the diazo substrate in an endergonic manner, implying that no catalyst poisoning is caused by the boron coordination (see Tables S1 and Figure S1).

[18] For existence of any correlation between ΔG_2_ and rC_a_−C_b_ and between ΔG4 and rC_a_−C_b_ see the Supporting Information.

[19] Gaussian 16, Revision C.01, M. J. Frisch, G. W. Trucks, H. B. Schlegel, G. E. Scuseria, M. A. Robb, J. R. Cheeseman, G. Scalmani, V. Barone, G. A. Petersson, H. Nakatsuji, X. Li, M. Caricato, A. V. Marenich, J. Bloino, B. G. Janesko, R. Gomperts, B. Mennucci, H. P. Hratchian, J. V. Ortiz, A. F. Izmaylov, J. L. Sonnenberg, D. Williams-Young, F. Ding, F. Lipparini, F. Egidi, J. Goings, B. Peng, A. Petrone, T. Henderson, D. Ranasinghe, V. G. Zakrzewski, J. Gao, N. Rega, G. Zheng, W. Liang, M. Hada, M. Ehara, K. Toyota, R. Fukuda, J. Hasegawa, M. Ishida, T. Nakajima, Y. Honda, O. Kitao, H. Nakai, T. Vreven, K. Throssell, J. A. Jr. Montgomery, J. E. Peralta, F. Ogliaro, M. J. Bearpark, J. J. Heyd, E. N. Brothers, K. N. Kudin, V. N. Staroverov, T. A. Keith, R. Kobayashi, J. Normand, K. Raghavachari, A. P. Rendell, J. C.; Burant, S. S. Iyengar, J. Tomasi, M. Cossi, J. M. Millam, M. Klene, C. Adamo, R. Cammi, J. W. Ochterski, R. L. Martin, K. Morokuma, O. Farkas, J. B. Foresman, D. J. Fox, Gaussian, Inc., Wallingford CT, **2016**.

[20] Y. Zhao , D. G. Truhlar , Acc. Chem. Res. 2008, 41, 157 **–** 167. 18186612 10.1021/ar700111a

[21] A. V. Marenich , C. J. Cramer , D. G. Truhlar , J. Phys. Chem. B. 2009, 113, 6378 **–** 6396. 19366259 10.1021/jp810292n

[22] P. C. Hariharan , J. Pople , Theor. Chim. Acta 1973, 28, 213 **–** 222.

[23a] K. Fukui , J. Phys. Chem. 1970, 74, 4161 **–** 4163;

[23b] K. Fukui , Acc. Chem. Res. 1981, 14, 363 **–** 368.

[24] F. Weigend , F. Furche , R. Ahlrichs , J. Chem. Phys. 2003, 119, 12753 **–** 12762.

[25] J. W. Ochterski, Thermochemistry, Gaussian, Inc., Wallingford, CT, **2000**.

[26] E. D. Glendening, J. K. Badenhoop, A. E. Reed, J. E. Carpenter, J. A. Bohmann, C. M. Morales, C. R. Landis, F. Weinhold, Theoretical Chemistry Institute, University of Wisconsin, Madison, WI, **2013**; http://nbo6.chem.wisc.edu/.

