# Supplementary material for: Understanding the Influence of Donor‐Acceptor Diazo Compounds on the Catalyst Efficiency of B(C6F5)3 Towards Carbene Formation
Source: Chemistry. 2022 Jan 27;28(11):e202104376. doi: 10.1002/chem.202104376 (PMC9303686; doi:10.1002/chem.202104376)
Supplement: Supplementary file 1 — Supporting Information [file CHEM-28-0-s001.pdf]

Chemistry–A European Journal

Supporting Information

Understanding the Influence of Donor-Acceptor Diazo Compounds on the Catalyst Efficiency of $B(C_6F_5)_3$ Towards Carbene Formation

Rasool Babaahmadi, Ayan Dasgupta, Christopher J. T. Hyland, Brian F. Yates, Rebecca L. Melen,* and Alireza Ariafard*

Table of Content

Catalyst poisoning and impact of the borane acidity on the catalyst efficiency	2
Correlation plot between $\Delta G^{\ddagger}_4$ and ΔG_4 (Figure S2)	4
Correlations between ΔG_2 and $r_{C^a\text{-}C^b}$ and between ΔG_4 and $r_{C^a\text{-}C^b}$	4
Cartesian coordinates and total energies for the calculated structures	6

Catalyst poisoning and impact of the borane acidity on the catalyst efficiency

Table S1 shows the free energy values for all species involved in the N_2 release from various diazo compounds with R′ = OMe catalysed by $B(C_6F_5)_3$. This table clearly shows that the boron catalyst binds to the diazo substrate in an endergonic manner with ΔG_b values ranging from 0.9 kcal/mol for R = NH_2 to 5.8 kcal/mol for R = NO_2. It follows that there is no catalyst poisoning caused by the coordination of the boron catalyst to the carbonyl group of the diazo molecule. This is also true when the borane coordinates to a R group with a strong electron donating feature such as OMe and NMe_2. This claim is supported by the endergonicity calculated for transformations given in Figure S1.

Our calculations also indicate that the catalyst efficiency could be affected by the nature of the borane catalyst. As shown in Table S2, the use of a borane with a higher acidity decreases the activation barrier to the N_2 release process more significantly. Accordingly, the more acidic the boron catalyst, the lower the activation barrier to the N_2 release and the more favourable thermodynamically the carbene formation (Table S2).

Table S1. Calculated free energy values for all species involved in the N_2 release from various diazo compounds with R′ = OMe catalyzed by $B(C_6F_5)_3$.

Entry	R	ΔG_b	$\Delta G^{\ddagger}_2$	ΔG_2
1	NMe_2	0.9	15.4	-13.8
2	NH_2	1.5	16.5	-11.9
3	OMe	2.5	21.1	-1.9
4	Me	2.2	25.1	5.9
5	F	3.9	26.3	6.1
6	H	2.5	25.9	6.5
7	Cl	3.2	27.7	9.8
8	CF_3	3.2	30.5	14.2
9	CN	4.7	32.2	14.9
10	NO_2	5.8	34.3	16.7

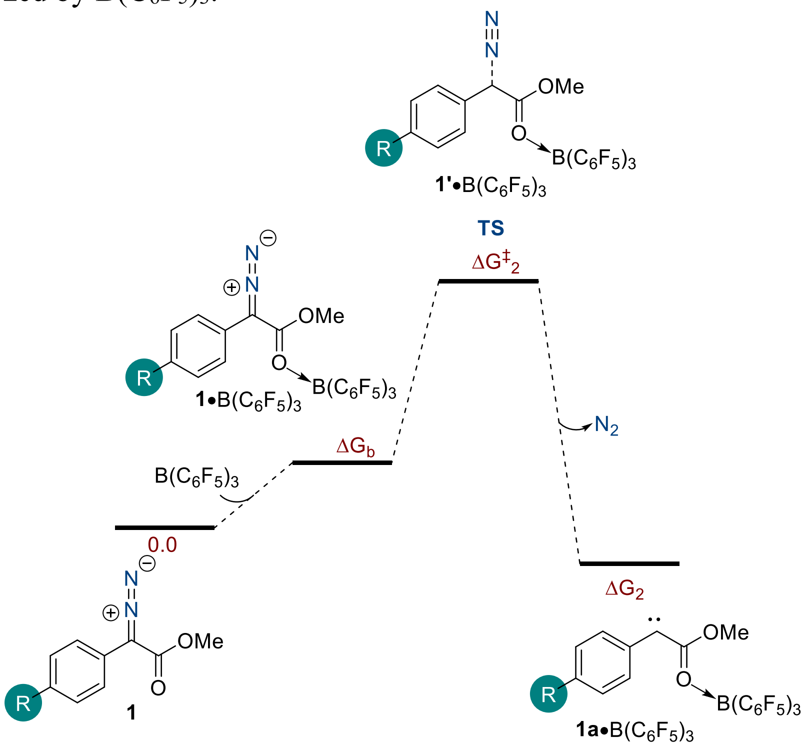

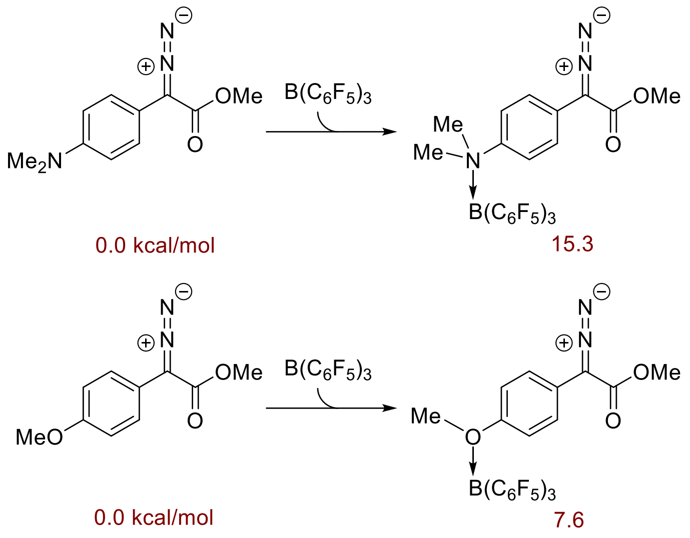

Figure S1. Calculated free energy for coordinating NMe_2 and OMe substituents to $B(C_6F_5)_3$.

Table S2. Calculated free energies for carbene formation catalysed by different boron catalyst.

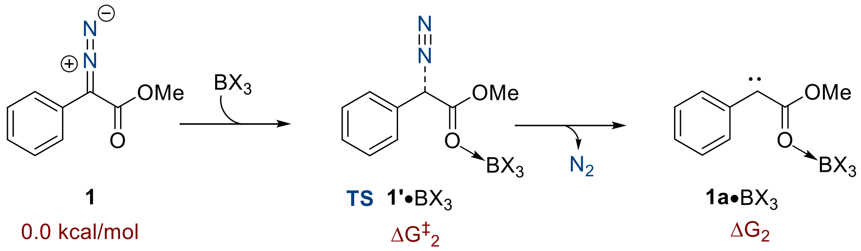

Entry	X	$\Delta G^{\ddagger}_2$	ΔG_2
1	Cl	21.2	3.0
2	H	23.7	4.5
3	F	24.3	5.3
4	(C_6F_5)	25.9	6.5
5	Ph	38.8	19.1

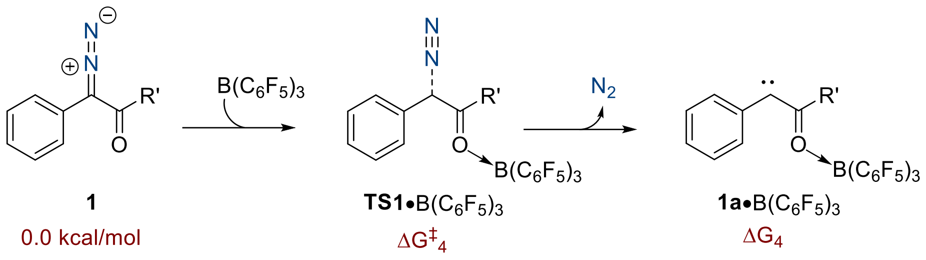

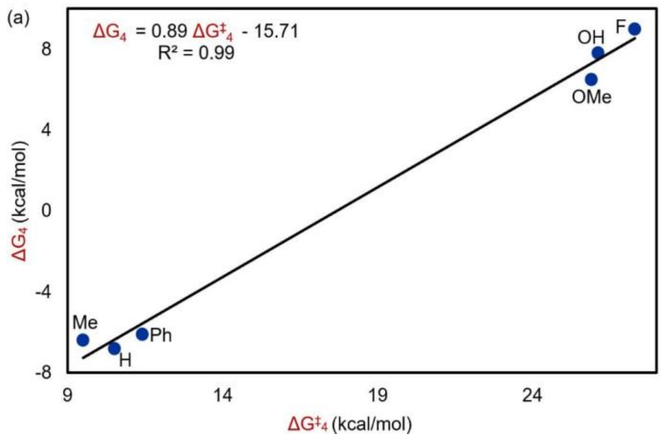

Figure S2. Correlation plot between $\Delta G^{\ddagger}_4$ and ΔG_4 for $B(C_6F_5)_3$-catalyzed carbene formation with R′ = H, Me, Ph, OMe, OH, and F.

Correlations between ΔG_2 and $r_{C^a\text{-}C^b}$ and between ΔG_4 and $r_{C^a\text{-}C^b}$. We also explored to see whether there is any correlation between ΔG_2 and $r_{C^a\text{-}C^b}$ as well as between ΔG_4 and $r_{C^a\text{-}C^b}$. We found a reasonable correlation with R^2 0.77 between the Gibbs free energy of N_2 release (ΔG_2) and $r_{C^a\text{-}C^b}$ in the carbene stabilized by $B(C_6F_5)_3$ (Figure S3). This moderate correlation demonstrates that both resonance structures **1a'**·$B(C_6F_5)_3$ and **1a''**·$B(C_6F_5)_3$ should contribute to stabilization of the carbene in the presence of the boron catalyst. In contrast, the lack of a good correlation with $R^2 = 0.21$ between ΔG_4 and $r_{C^a\text{-}C^b}$ (Figure S4) suggests that a change in the R′ group only changes the contribution of the resonance structure **1a''**·$B(C_6F_5)_3$ and not **1a'**·$B(C_6F_5)_3$.

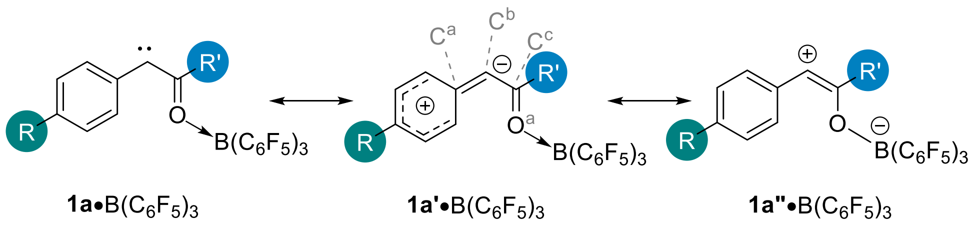

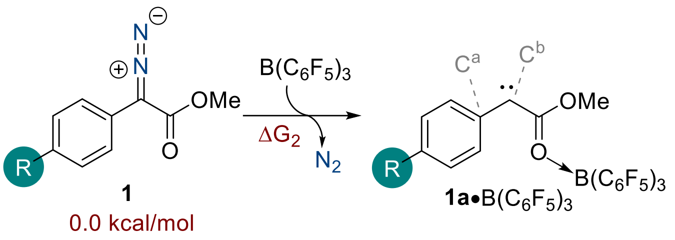

Entry	R	ΔG_2	$r_{C^a\text{-}C^b}$ (Å)
1	NMe_2	-13.8	1.342
2	NH_2	-11.9	1.343
3	OMe	-1.9	1.345
4	Me	5.9	1.378
5	F	6.1	1.356
6	H	6.5	1.364
7	Cl	9.8	1.388
8	CF_3	14.2	1.374
9	CN	14.9	1.398
10	NO_2	16.7	1.401

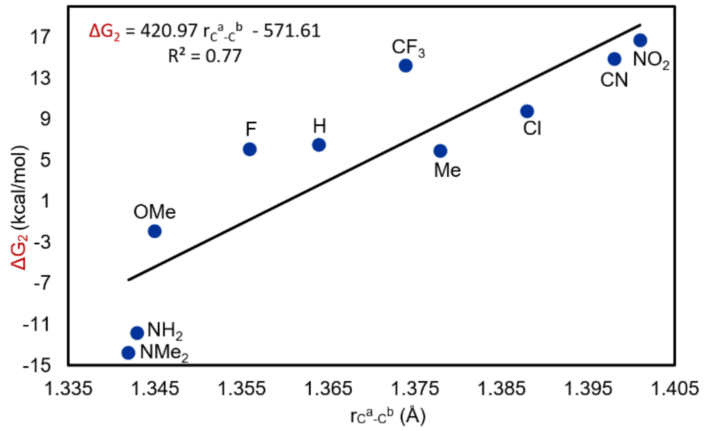

Figure S3. Correlation plot between ΔG_2 and $r_{C^a\text{-}C^b}$ for $B(C_6F_5)_3$-catalyzed carbene formation with different R group while keeping R′ = OMe.

Entry	R′	ΔG_4	$r_{C^a\text{-}C^b}$ (Å)
1	Me	-6.4	1.365
2	H	-6.8	1.36
3	Ph	-6.1	1.365
4	OMe	6.5	1.364
5	OH	7.8	1.363
6	F	9.0	1.353

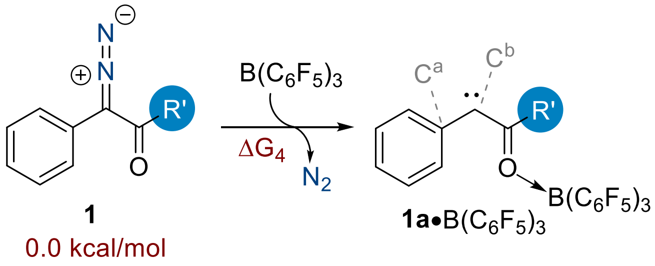

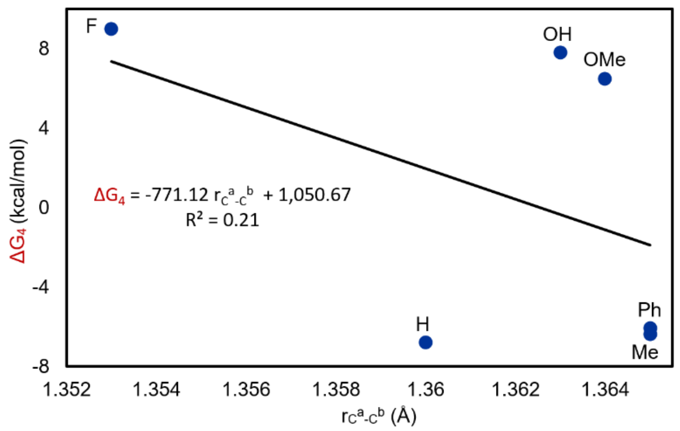

Figure S4. Correlation plot between ΔG_4 and $r_{C^a\text{-}C^b}$ for $B(C_6F_5)_3$-catalyzed carbene formation with different R′ group while keeping R = H

Table S3. Cartesian coordinates and total energies for the calculated structures.

1 (R = NMe_2 , R′ = OMe)
E(SMD/M06-2X/6-31G(d)) = -741.3517888 au
H(SMD/M06-2X/6-31G(d)) = -741.100277 au
G(SMD/M06-2X/6-31G(d)) = -741.161867 au
E(SMD/M06-2X/def2-TZVP//SMD/M06-2X /6-31G(d)) = -741.6425442 au

C	-1.72840400	0.42723100	-0.07754000
N	-2.20852800	1.64063400	-0.22478600
N	-2.60105100	2.69131500	-0.34765000
C	-0.26385400	0.25426400	-0.06972800
C	0.32895000	-0.98837700	-0.32510400
C	0.58576100	1.33852100	0.18274400
C	1.70882600	-1.13952500	-0.32376700
H	-0.29608600	-1.85267100	-0.51466200
C	1.96535400	1.19948800	0.17385600
H	0.16836900	2.31866200	0.40280400
C	2.57043800	-0.04733000	-0.09097200
H	2.11478800	-2.12610500	-0.51307700
H	2.57203000	2.07169700	0.38548000
C	-2.73169400	-0.62153000	0.10382800
O	-2.47941500	-1.79400600	0.28514700
O	-3.98189000	-0.13030000	0.04420600
C	-5.02369600	-1.09550200	0.21593400
H	-5.95553700	-0.53658800	0.14228700
H	-4.97311500	-1.85502000	-0.56734200
H	-4.94384700	-1.57409300	1.19433300
N	3.94597200	-0.18829800	-0.12849700
C	4.76446100	0.88246000	0.41025900
H	4.54251200	1.09245700	1.46732500
H	5.81448200	0.60036200	0.32549100
H	4.62172100	1.80595000	-0.15987500
C	4.50392300	-1.52809300	-0.11874500
H	4.19071700	-2.08562000	-1.00693100
H	5.59200600	-1.45668600	-0.14007100
H	4.20726500	-2.10310300	0.77134900

1 (R = NH_2 , R′ = OMe)
E(SMD/M06-2X/6-31G(d)) = -662.7793238 au
H(SMD/M06-2X/6-31G(d)) = -662.588176 au
G(SMD/M06-2X/6-31G(d)) = -662.642734 au
E(SMD/M06-2X/def2-TZVP//SMD/M06-2X /6-31G(d)) = -663.0469861 au

C	0.94723900	0.41782400	-0.05628500
N	1.36404100	1.65748300	-0.17617100
N	1.70127200	2.72941100	-0.27517800
C	-0.50715100	0.17418200	-0.01854600
C	-1.04135700	-1.09339700	-0.28771800

C	-1.39794700	1.21770400	0.26790200
C	-2.41316300	-1.30547600	-0.26039700
H	-0.37816500	-1.91889200	-0.51533900
C	-2.76921500	1.00977000	0.28260300
H	-1.01955400	2.21369300	0.48630900
C	-3.30397800	-0.26034100	0.02213500
H	-2.80730200	-2.29734100	-0.46693000
H	-3.43930300	1.83527900	0.50822900
C	2.00466700	-0.58538000	0.06685300
O	1.81446200	-1.77430700	0.21322100
O	3.22723200	-0.03058800	-0.00276700
C	4.31890300	-0.94756000	0.11585200
H	5.22014400	-0.34034600	0.04443500
H	4.28245200	-1.46217700	1.07840100
H	4.28998100	-1.68166400	-0.69235500
N	-4.67458100	-0.48993400	0.10622300
H	-5.24643100	0.32965600	-0.06632800
H	-4.99849300	-1.28172300	-0.43856700

1 (R = OMe , R′ = OMe)
E(SMD/M06-2X/6-31G(d)) = -721.9211765 au
H(SMD/M06-2X/6-31G(d)) = -721.712349 au
G(SMD/M06-2X/6-31G(d)) = -721.769653 au
E(SMD/M06-2X/def2-TZVP//SMD/M06-2X /6-31G(d)) = -722.2094288 au

C	-1.34496200	0.41390400	0.06490700
N	-1.72347400	1.66725100	0.17693300
N	-2.02944200	2.74802000	0.27202400
C	0.10130100	0.12559600	0.05009800
C	0.58742300	-1.16630000	0.31184100
C	1.02652700	1.14101900	-0.20776900
C	1.94909300	-1.41959000	0.30367100
H	-0.10622400	-1.97222700	0.51549000
C	2.39757100	0.89569400	-0.20530500
H	0.68458900	2.15057500	-0.42224300
C	2.86705100	-0.39442000	0.04787000
H	2.32716900	-2.41768800	0.50226500
H	3.08039900	1.71205500	-0.40954900
C	-2.43257600	-0.55453000	-0.07660600
O	-2.27901100	-1.74912900	-0.21837300
O	-3.63618900	0.04135000	-0.03113300
C	-4.75628200	-0.83864600	-0.16751800
H	-5.63754000	-0.20138300	-0.11090900
H	-4.72120500	-1.35450700	-1.12941700
H	-4.76495900	-1.57263100	0.64113500
O	4.17689400	-0.74625300	0.06659400
C	5.12901300	0.26888100	-0.19934500
H	6.10623500	-0.21120400	-0.14138700

H	4.99009000	0.69144400	-1.20118300
H	5.07334200	1.07127600	0.54532700

1 (R = Me , R′ = OMe)
E(SMD/M06-2X/6-31G(d)) = -646.7401709 au
H(SMD/M06-2X/6-31G(d)) = -646.537515 au
G(SMD/M06-2X/6-31G(d)) = -646.594206 au
E(SMD/M06-2X/def2-TZVP//SMD/M06-2X /6-31G(d)) = -646.9939794 au

C	0.95708800	0.40503100	-0.00245900
N	1.37580100	1.65277200	-0.00465100
N	1.71471300	2.72665600	-0.00649100
C	-0.49813800	0.16913600	-0.00292000
C	-1.02835000	-1.12833500	-0.01617200
C	-1.39155800	1.25152600	0.00494300
C	-2.40684800	-1.32129000	-0.01945200
H	-0.36366300	-1.98209700	-0.02718600
C	-2.76376800	1.04060100	0.00104400
H	-1.02034700	2.27284500	0.01059600
C	-3.30169900	-0.24987200	-0.00825400
H	-2.79344300	-2.33762400	-0.03309100
H	-3.43092000	1.89928300	0.00387100
C	2.02587100	-0.59596500	0.00313200
O	1.85526100	-1.79651300	0.00792100
O	3.23990000	-0.01977100	0.00250800
C	4.34526400	-0.92876700	0.00799900
H	5.23715100	-0.30392200	0.00640400
H	4.32009200	-1.55400200	0.90302000
H	4.32241800	-1.56190500	-0.88152600
C	-4.79136900	-0.47114400	0.01846200
H	-5.31750800	0.30588200	-0.54397900
H	-5.17287700	-0.44639700	1.04603900
H	-5.05475300	-1.44330700	-0.40751600

1 (R = F , R′ = OMe)
E(SMD/M06-2X/6-31G(d)) = -706.6475084 au
H(SMD/M06-2X/6-31G(d)) = -706.481851 au
G(SMD/M06-2X/6-31G(d)) = -706.535606 au
E(SMD/M06-2X/def2-TZVP//SMD/M06-2X /6-31G(d)) = -706.9356438 au

C	-0.92007400	0.40909500	0.00020800
N	-1.33237800	1.65892700	0.00015000
N	-1.66874700	2.73343200	0.00011300
C	0.53360200	0.16394100	0.00008000
C	1.04963500	-1.14088000	0.00004300
C	1.43177100	1.24291700	-0.00001000
C	2.42395400	-1.35718900	-0.00007900
H	0.37593600	-1.98718400	0.00011100
C	2.80466600	1.03358000	-0.00013200
H	1.06718500	2.26589200	0.00001500

C	3.27902600	-0.26834500	-0.00016400
H	2.83219800	-2.36234700	-0.00010700
H	3.50161000	1.86478000	-0.00020100
C	-1.99312800	-0.58756300	0.00035800
O	-1.82601100	-1.78869800	0.00015400
O	-3.20394300	-0.00674200	0.00001500
C	-4.31348400	-0.91131200	-0.00023500
H	-5.20251500	-0.28251100	-0.00047400
H	-4.29157000	-1.54038600	-0.89260000
H	-4.29200900	-1.54033300	0.89217800
F	4.60898400	-0.47848500	-0.00028200

1 (R = H , R′ = OMe)
E(SMD/M06-2X/6-31G(d)) = -607.4428884 au
H(SMD/M06-2X/6-31G(d)) = -607.269705 au
G(SMD/M06-2X/6-31G(d)) = -607.321306 au
E(SMD/M06-2X/def2-TZVP//SMD/M06-2X /6-31G(d)) = -607.6830308 au

C	0.49784700	0.38998100	-0.00015200
N	0.84709000	1.65948200	-0.00007700
N	1.13074000	2.74874800	0.00000000
C	-0.94166400	0.07353700	-0.00010300
C	-1.39154800	-1.25520500	0.00006200
C	-1.89242700	1.10677100	-0.00013600
C	-2.75635800	-1.53044400	0.00020600
H	-0.67468000	-2.06553200	0.00010800
C	-3.25196700	0.81918900	-0.00000800
H	-1.57626500	2.14637100	-0.00024900
C	-3.69477400	-0.50204200	0.00017000
H	-3.08398100	-2.56590700	0.00036400
H	-3.96682700	1.63660900	-0.00003400
H	-4.75697100	-0.72552200	0.00027300
C	1.62220200	-0.54891800	-0.00011600
O	1.52083100	-1.75718800	-0.00055500
O	2.80087500	0.09609100	0.00041200
C	3.95705300	-0.74762500	0.00020100
H	4.81135200	-0.07230200	0.00068400
H	3.96920000	-1.37744600	0.89225800
H	3.96952700	-1.37657100	-0.89246600

1 (R = Cl , R′ = OMe)
E(SMD/M06-2X/6-31G(d)) = -1067.014275 au
H(SMD/M06-2X/6-31G(d)) = -1066.849697 au
G(SMD/M06-2X/6-31G(d)) = -1066.905033 au
E(SMD/M06-2X/def2-TZVP//SMD/M06-2X /6-31G(d)) = -1067.287815 au

C	-1.33908500	0.41177600	0.00024400
N	-1.78954600	1.64952800	0.00015800
N	-2.15880000	2.71226000	0.00002300
C	0.11887800	0.21389600	0.00011500

C	0.67651100	-1.07312000	0.00006000
C	0.98340200	1.31978300	0.00004400
C	2.05663400	-1.24571400	-0.00006200
H	0.03171900	-1.94175000	0.00011500
C	2.36154400	1.15246800	-0.00008000
H	0.58932000	2.33209100	0.00008800
C	2.88896500	-0.13378100	-0.00013200
H	2.47776800	-2.24567100	-0.00010200
H	3.01721300	2.01672200	-0.00013500
C	-2.38079300	-0.61837600	0.00040600
O	-2.17519200	-1.81322700	0.00021200
O	-3.60805700	-0.07492300	0.00005000
C	-4.69041900	-1.01237200	-0.00019700
H	-5.59754100	-0.41003500	-0.00045000
H	-4.64949300	-1.64024500	-0.89266300
H	-4.64994700	-1.64018500	0.89233000
Cl	4.62538500	-0.35209400	-0.00029100

1 (R = CF_3 , R′ = OMe)
E(SMD/M06-2X/6-31G(d)) = -944.3819053 au
H(SMD/M06-2X/6-31G(d)) = -944.201138 au
G(SMD/M06-2X/6-31G(d)) = -944.259779 au
E(SMD/M06-2X/def2-TZVP//SMD/M06-2X /6-31G(d)) = -944.7764732 au

C	1.98783700	0.41642400	-0.00669600
N	2.46766700	1.64450500	0.00220600
N	2.86428700	2.69631100	0.00928600
C	0.52766100	0.25559200	-0.01797100
C	-0.05836700	-1.02034300	-0.03019700
C	-0.30851100	1.38462100	-0.01901700
C	-1.44057400	-1.15376200	-0.04025900
H	0.56856900	-1.90162100	-0.03412500
C	-1.68827100	1.24520600	-0.02800600
H	0.11300300	2.38578200	-0.01523200
C	-2.25717500	-0.02609700	-0.03918600
H	-1.88220900	-2.14549600	-0.05296800
H	-2.31937600	2.12804000	-0.03126000
C	3.00635000	-0.63780300	0.00069600
O	2.77404300	-1.82742400	-0.00360400
O	4.24457700	-0.12122400	0.01316300
C	5.30687400	-1.08173600	0.02251700
H	6.22647600	-0.49873900	0.02931600
H	5.24540600	-1.70743700	0.91528300
H	5.25966900	-1.70922500	-0.86983400
C	-3.74497200	-0.18585000	0.00800300
F	-4.38488100	0.87193800	-0.51570700
F	-4.19985200	-0.32280900	1.26806700
F	-4.15630100	-1.27416600	-0.66318200

1 (R = CN , R′ = OMe)
E(SMD/M06-2X/6-31G(d)) = -699.6626854 au
H(SMD/M06-2X/6-31G(d)) = -699.489103 au
G(SMD/M06-2X/6-31G(d)) = -699.545081 au
E(SMD/M06-2X/def2-TZVP//SMD/M06-2X /6-31G(d)) = -699.9374204 au

C	1.19534000	0.40320800	0.00021000
N	1.64135800	1.64554200	0.00025400
N	2.00956800	2.70690100	0.00025300
C	-0.25683900	0.20319100	0.00011100
C	-0.80880700	-1.08914400	0.00053000
C	-1.12292800	1.31143300	-0.00022600
C	-2.18450100	-1.26280800	0.00042000
H	-0.15849700	-1.95321300	0.00085900
C	-2.49607200	1.13969300	-0.00030900
H	-0.72766000	2.32279800	-0.00034900
C	-3.03523300	-0.15228600	-0.00002600
H	-2.60293000	-2.26394600	0.00068000
H	-3.15316700	2.00295200	-0.00059300
C	2.24331100	-0.62309700	-0.00014200
O	2.04269200	-1.81816900	-0.00032000
O	3.46557900	-0.07174400	-0.00022800
C	4.55550300	-1.00144600	-0.00011600
H	5.45773900	-0.39198000	-0.00031100
H	4.51891800	-1.62890600	0.89272400
H	4.51878300	-1.62927300	-0.89269600
C	-4.45945500	-0.33486900	-0.00012100
N	-5.60824900	-0.48135600	-0.00021000

1 (R = NO_2 , R′ = OMe)
E(SMD/M06-2X/6-31G(d)) = -811.8736413 au
H(SMD/M06-2X/6-31G(d)) = -811.695066 au
G(SMD/M06-2X/6-31G(d)) = -811.753158 au
E(SMD/M06-2X/def2-TZVP//SMD/M06-2X /6-31G(d)) = -812.2028104 au

C	1.59835900	0.41344400	-0.00732100
N	2.06179900	1.65014800	-0.02495600
N	2.44695900	2.70472200	-0.04054800
C	0.14564600	0.23392800	-0.00175100
C	-0.42192200	-1.05237600	-0.03150900
C	-0.70615000	1.35431300	0.03121800
C	-1.79921700	-1.21064400	-0.02953000
H	0.21761700	-1.92388300	-0.05616000
C	-2.08152600	1.20146200	0.03179900
H	-0.29807000	2.36020100	0.05951500
C	-2.61142900	-0.08387700	0.00107400
H	-2.24173200	-2.19925800	-0.05297700
H	-2.73731100	2.06332800	0.05691700
C	2.63260100	-0.62755400	0.00949700

O	2.41489300	-1.81921800	0.03071300
O	3.86148100	-0.09267800	-0.00031600
C	4.93952100	-1.03646400	0.01622900
H	5.84941700	-0.43866000	0.00591900
H	4.89440000	-1.64770700	0.91979100
H	4.89451900	-1.67885100	-0.86552100
N	-4.06111200	-0.25292700	0.00128100
O	-4.75577100	0.75056300	0.02207100
O	-4.50655800	-1.38893600	-0.01948700

1′ (R = NMe_2 , R′ = OMe)
E(SMD/M06-2X/6-31G(d)) = -741.3027561 au
H(SMD/M06-2X/6-31G(d)) = -741.05419 au
G(SMD/M06-2X/6-31G(d)) = -741.116547 au
E(SMD/M06-2X/def2-TZVP//SMD/M06-2X /6-31G(d)) = -741.5962532 au

C	-1.70966600	0.53812900	-0.59698300
N	-2.25336200	1.74601400	0.72638500
N	-2.39647400	2.79612800	1.03758800
C	-0.31292200	0.30120000	-0.36837200
C	0.63294700	1.23290300	-0.85249400
C	0.19542900	-0.83223400	0.31066700
C	1.98832800	1.06177300	-0.68104900
H	0.26600100	2.11133900	-1.37737800
C	1.54555500	-1.03879100	0.47564800
H	-0.50174800	-1.57372300	0.69237800
C	2.48902300	-0.08980000	-0.01235800
H	2.67199800	1.80596600	-1.07019900
H	1.88658700	-1.92966800	0.98872100
C	-2.67298100	-0.53993100	-0.44653900
O	-2.55964300	-1.56851300	-1.09662200
O	-3.75998800	-0.27601700	0.30729100
C	-4.77961200	-1.27454200	0.25801300
H	-5.57061400	-0.92049200	0.91877800
H	-5.16085900	-1.38866000	-0.75959500
H	-4.39765200	-2.23590200	0.60943600
N	3.82074600	-0.27968000	0.15260200
C	4.31331300	-1.46582600	0.83436100
H	3.94260500	-1.51519200	1.86451500
H	4.01028100	-2.38023500	0.31130700
H	5.40147800	-1.43011400	0.86227000
C	4.76767500	0.69582900	-0.36317700
H	4.61578100	1.67901400	0.09700300
H	5.77875600	0.36142000	-0.13476300
H	4.67553800	0.80299200	-1.45014200

1′ (R = NH_2 , R′ = OMe)
E(SMD/M06-2X/6-31G(d)) = -662.7290712 au
H(SMD/M06-2X/6-31G(d)) = -662.541439 au

G(SMD/M06-2X/6-31G(d)) = -662.59738 au
E(SMD/M06-2X/def2-TZVP//SMD/M06-2X /6-31G(d)) = -662.9994334 au

C	0.91985500	0.44587800	0.65168400
N	1.42965900	1.84343800	-0.49629900
N	1.53355700	2.92925300	-0.66821300
C	-0.45530300	0.16707000	0.34544500
C	-1.46142100	0.98524600	0.90922400
C	-0.87613500	-0.89880700	-0.48744300
C	-2.79934500	0.76733400	0.66567700
H	-1.15489200	1.80795800	1.55000500
C	-2.20867400	-1.14728700	-0.72391500
H	-0.12634400	-1.54831900	-0.93139800
C	-3.20132500	-0.31272800	-0.15455000
H	-3.55837700	1.40746300	1.10615900
H	-2.51753500	-1.97748600	-1.35308100
C	1.94596300	-0.55178800	0.39524500
O	1.87415200	-1.65877700	0.90748200
O	3.03315400	-0.13708000	-0.28581600
C	4.10594600	-1.07876800	-0.33368400
H	4.89128100	-0.60017000	-0.91821800
H	4.46818100	-1.30421400	0.67219600
H	3.78700700	-2.00471300	-0.81798400
N	-4.51628900	-0.56874300	-0.36349500
H	-5.20563000	0.12496500	-0.10598500
H	-4.78799800	-1.22316500	-1.08506600

1′ (R = OMe , R′ = OMe)
E(SMD/M06-2X/6-31G(d)) = -721.8675325 au
H(SMD/M06-2X/6-31G(d)) = -721.661926 au
G(SMD/M06-2X/6-31G(d)) = -721.720547 au
E(SMD/M06-2X/def2-TZVP//SMD/M06-2X /6-31G(d)) = -722.1584724 au

C	1.31639900	0.43268600	0.70148300
N	1.80106000	1.88123100	-0.47934900
N	1.93874100	2.96074200	-0.65501700
C	-0.05425500	0.12938600	0.35922900
C	-1.07306400	0.92825100	0.90920200
C	-0.43637600	-0.94087700	-0.48509100
C	-2.41358900	0.69818900	0.63866400
H	-0.78863700	1.74819100	1.56282200
C	-1.76315500	-1.20002300	-0.74656500
H	0.32975300	-1.57999300	-0.91476300
C	-2.76216100	-0.37631500	-0.19234000
H	-3.17199700	1.33641300	1.07578800
H	-2.06866800	-2.02393900	-1.38332200
C	2.35910800	-0.54047300	0.41223900
O	2.30680200	-1.64693200	0.92686400
O	3.42149300	-0.10768100	-0.28833300

C	4.50742100	-1.03479400	-0.36655600
H	5.27173600	-0.54101100	-0.96567600
H	4.89423900	-1.25971800	0.63000600
H	4.18917400	-1.96142900	-0.84957600
O	-4.02364600	-0.70065000	-0.51543000
C	-5.07893500	0.09812000	0.00593600
H	-5.99960000	-0.33644600	-0.38240100
H	-5.09055600	0.06646100	1.10023500
H	-4.98960000	1.13486600	-0.33456400

1′ (R = Me , R′ = OMe)

E(SMD/M06-2X/6-31G(d)) = -646.6829413 au

H(SMD/M06-2X/6-31G(d)) = -646.483853 au

G(SMD/M06-2X/6-31G(d)) = -646.542379 au

E(SMD/M06-2X/def2-TZVP//SMD/M06-2X /6-31G(d)) = -646.9396283 au

C	0.93846300	0.41980300	0.73059800
N	1.44194700	1.89186600	-0.47169700
N	1.63280500	2.96230200	-0.64744300
C	-0.44610100	0.15601300	0.38232700
C	-1.43598300	1.00826400	0.90526100
C	-0.85428600	-0.92270600	-0.42924600
C	-2.77794800	0.80084000	0.62186800
H	-1.12551400	1.83789000	1.53450900
C	-2.19716600	-1.14228700	-0.68658500
H	-0.10657100	-1.59679200	-0.83757600
C	-3.17830600	-0.28187000	-0.17065000
H	-3.52983700	1.47378100	1.02492000
H	-2.50075000	-1.98257200	-1.30560000
C	1.95490800	-0.57114400	0.41223500
O	1.89027400	-1.67072200	0.93992700
O	3.00191100	-0.16664900	-0.32317100
C	4.06724500	-1.11662500	-0.42535200
H	4.82270100	-0.64264700	-1.05086800
H	4.47813500	-1.34044900	0.56166800
H	3.71586600	-2.04012700	-0.89078600
C	-4.63403300	-0.54026800	-0.44314200
H	-5.02590900	-1.28125500	0.26368700
H	-5.22745100	0.37093000	-0.33325400
H	-4.78218600	-0.93908700	-1.45064800

1′ (R = F , R′ = OMe)

E(SMD/M06-2X/6-31G(d)) = -706.5903819 au

H(SMD/M06-2X/6-31G(d)) = -706.428245 au

G(SMD/M06-2X/6-31G(d)) = -706.48346 au

E(SMD/M06-2X/def2-TZVP//SMD/M06-2X /6-31G(d)) = -706.8811168 au

C	-0.89536700	0.41812000	-0.73461600
N	-1.39150100	1.89971500	0.46656700
N	-1.58294300	2.96944400	0.64449700
C	0.48426900	0.14174700	-0.37610800

C	1.48326100	0.97921200	-0.90982000
C	0.87271200	-0.93383000	0.45038500
C	2.82395000	0.77013000	-0.62573500
H	1.18223000	1.80075200	-1.55292000
C	2.20905600	-1.17495200	0.72255500
H	0.11553900	-1.59521400	0.86047000
C	3.15521200	-0.30955800	0.18306800
H	3.60752800	1.40645200	-1.02182700
H	2.53281300	-2.00183900	1.34516200
C	-1.92258900	-0.56217200	-0.41560000
O	-1.85887300	-1.66699200	-0.93196700
O	-2.97298800	-0.14218300	0.30407900
F	4.44626000	-0.53096600	0.45434500
C	-4.04682800	-1.08324400	0.40640500
H	-4.80484000	-0.59613000	1.01845700
H	-4.44883600	-1.31520100	-0.58231300
H	-3.70683200	-2.00355000	0.88630800

1′ (R = H , R′ = OMe)
E(SMD/M06-2X/6-31G(d)) = -607.3840118 au
H(SMD/M06-2X/6-31G(d)) = -607.214398 au
G(SMD/M06-2X/6-31G(d)) = -607.2677 au
E(SMD/M06-2X/def2-TZVP//SMD/M06-2X /6-31G(d)) = -607.6267506 au

C	0.45839500	0.37232200	0.73457600
N	0.89988000	1.93395900	-0.40696000
N	1.03709100	3.01767700	-0.54434700
C	-0.88956900	0.02630100	0.30796300
C	-1.95244700	0.81577900	0.78502000
C	-1.18003500	-1.07210900	-0.52569200
C	-3.26429900	0.53004400	0.43004300
H	-1.72205300	1.65515200	1.43516100
C	-2.49365800	-1.37659600	-0.85429300
H	-0.36885900	-1.69556700	-0.89025100
C	-3.53317000	-0.57051400	-0.38457700
H	-4.07703200	1.14956100	0.79548700
H	-2.71260100	-2.23225000	-1.48527100
H	-4.55922200	-0.80508000	-0.65256600
C	1.55846500	-0.52659500	0.42254500
O	1.55561600	-1.64356900	0.91655300
O	2.59524600	-0.02238300	-0.26124700
C	3.73190600	-0.88784900	-0.35384700
H	4.47129600	-0.33794200	-0.93471800
H	4.12269500	-1.11489100	0.64047400
H	3.46655000	-1.81752300	-0.86203800

1′ (R = Cl , R′ = OMe)
E(SMD/M06-2X/6-31G(d)) = -1066.954829 au
H(SMD/M06-2X/6-31G(d)) = -1066.793804 au
G(SMD/M06-2X/6-31G(d)) = -1066.850843 au
E(SMD/M06-2X/def2-TZVP//SMD/M06-2X /6-31G(d)) = -1067.231357 au

C	-1.32994400	0.42139900	-0.75577700
N	-1.83680000	1.89071000	0.49019100
N	-2.07648900	2.94639900	0.68939400
C	0.07080000	0.19413400	-0.43328800
C	1.01706400	1.10615600	-0.93448900
C	0.52562400	-0.90474500	0.32164900
C	2.37199800	0.94735700	-0.68270300
H	0.66794100	1.94754700	-1.52576000
C	1.87877100	-1.09675700	0.55423100
H	-0.18870600	-1.62584600	0.70704700
C	2.78340900	-0.15988600	0.05563800
H	3.10045400	1.65553200	-1.06186300
H	2.23260200	-1.94926100	1.12381000
C	-2.31050700	-0.59493500	-0.40541600
O	-2.22559300	-1.68960800	-0.93994400
O	-3.34186200	-0.22162300	0.36245100
Cl	4.48477600	-0.38510100	0.36187400
C	-4.37515100	-1.20362200	0.50187500
H	-5.11951800	-0.75268400	1.15669000
H	-4.81531900	-1.43647500	-0.47014800
H	-3.97839200	-2.11662800	0.95089200

1′ (R = CF_3, R′ = OMe)
E(SMD/M06-2X/6-31G(d)) = -944.3198923 au
H(SMD/M06-2X/6-31G(d)) = -944.141762 au
G(SMD/M06-2X/6-31G(d)) = -944.204824 au
E(SMD/M06-2X/def2-TZVP//SMD/M06-2X /6-31G(d)) = -944.7168893 au

C	-1.98505800	0.40539000	-0.78638700
N	-2.50056000	1.89818000	0.47332100
N	-2.79257700	2.93908700	0.67746900
C	-0.56673100	0.22854800	-0.49200100
C	0.32008100	1.23162500	-0.91760600
C	-0.04926500	-0.91268900	0.15270300
C	1.68522900	1.12158400	-0.68536500
H	-0.08175600	2.10159900	-1.42842400
C	1.31569200	-1.04663200	0.34928400
H	-0.72186300	-1.70252600	0.47091900
C	2.17239600	-0.02244100	-0.06001700
H	2.36408100	1.90491700	-1.00334500
H	1.71885100	-1.93109700	0.83386800
C	-2.93045200	-0.62626700	-0.39182800
O	-2.83929200	-1.71653800	-0.93463700
O	-3.93301100	-0.27774500	0.42028800
C	-4.93760000	-1.28380500	0.60253500
H	-5.66378900	-0.84925800	1.28788300
H	-5.41168600	-1.52631300	-0.35085800
H	-4.50070100	-2.18648500	1.03451700
C	3.64483900	-0.19533000	0.18838000
F	3.90470400	-0.35746300	1.49618000
F	4.12225200	-1.28541900	-0.43368000
F	4.36331600	0.85206800	-0.23399900

1′ (R = CN, R′ = OMe)
E(SMD/M06-2X/6-31G(d)) = -699.5994266 au
H(SMD/M06-2X/6-31G(d)) = -699.429465 au
G(SMD/M06-2X/6-31G(d)) = -699.487571 au
E(SMD/M06-2X/def2-TZVP//SMD/M06-2X /6-31G(d)) = -699.8765275 au

C	1.19212900	0.34945600	0.78217200
N	1.69876400	1.93404700	-0.39772300
N	1.98550000	2.98594600	-0.54297200
C	-0.20995900	0.14733600	0.43703000
C	-1.12366100	1.16835100	0.76015100
C	-0.69013900	-1.04378200	-0.13976800
C	-2.47369100	1.02669500	0.48687300
H	-0.75237100	2.07790600	1.22285400
C	-2.04609500	-1.21587600	-0.37198500
H	0.00139400	-1.84496900	-0.37741000
C	-2.93164000	-0.17418300	-0.07074200
H	-3.17596500	1.81927200	0.72068200
H	-2.42140000	-2.13779600	-0.80285600
C	2.18915800	-0.62479800	0.37359100
O	2.13370000	-1.73111700	0.88824400
O	3.18260900	-0.21330900	-0.41641600
C	4.23185300	-1.17113800	-0.61398300
H	4.94992000	-0.68354100	-1.27145700
H	4.69941300	-1.42557700	0.33937400
H	3.83912500	-2.07512400	-1.08372200
C	-4.33692900	-0.34207900	-0.33205800
N	-5.46665500	-0.47637300	-0.54355900

1′ (R = NO_2, R′ = OMe)
E(SMD/M06-2X/6-31G(d)) = -811.8094114 au
H(SMD/M06-2X/6-31G(d)) = -811.63455 au
G(SMD/M06-2X/6-31G(d)) = -811.694962 au
E(SMD/M06-2X/def2-TZVP//SMD/M06-2X /6-31G(d)) = -812.140919 au

C	1.59444400	0.35306000	0.79653700
N	2.11452800	1.94106800	-0.40783500
N	2.43868600	2.97757700	-0.57957600
C	0.18583500	0.17595800	0.46763700
C	-0.69445600	1.25095000	0.69879100
C	-0.33316600	-1.04969600	0.00854000
C	-2.04933200	1.12794900	0.44051600
H	-0.29706600	2.18784600	1.07720000
C	-1.69583500	-1.20147000	-0.20380000
H	0.33191500	-1.89037500	-0.15447200
C	-2.52258700	-0.10440300	0.00219100
H	-2.73378200	1.95212100	0.59873000
H	-2.11050400	-2.14146500	-0.54687500
C	2.57545200	-0.63069800	0.37546400
O	2.51296400	-1.73076700	0.90260600
O	3.55571700	-0.23836700	-0.43695100
C	4.58985500	-1.21111100	-0.64785500
H	5.29939400	-0.73748700	-1.32431400

H	5.07351200	-1.46199200	0.29830300
H	4.17590100	-2.11383100	-1.10123500
N	-3.96263400	-0.25317600	-0.24871400
O	-4.37001100	-1.34713600	-0.59741800
O	-4.67050600	0.72672800	-0.09606100

1a (R = NMe_2, R′ = OMe)
E(SMD/M06-2X/6-31G(d)) = -631.8392398 au
H(SMD/M06-2X/6-31G(d)) = -631.599998 au
G(SMD/M06-2X/6-31G(d)) = -631.656697 au
E(SMD/M06-2X/def2-TZVP//SMD/M06-2X /6-31G(d)) = -632.092357 au

C	-1.94647600	-1.24942700	0.08441200
C	-0.63498000	-0.79457100	0.06103500
C	0.43078400	-1.73992500	-0.11074700
C	-0.23824800	0.58193700	0.18793200
C	1.74483800	-1.37431800	-0.15221000
H	0.15550500	-2.78625600	-0.21040000
C	1.06642000	0.97569500	0.15187800
H	-1.01242100	1.33412600	0.32353100
C	2.11183800	0.00531300	-0.01678800
H	2.51081500	-2.12769700	-0.28534200
H	1.31559100	2.02465000	0.25177500
C	-3.00185700	-0.28143900	0.30539100
O	-3.44797500	0.02183100	1.40141700
O	-3.54162500	0.17026000	-0.85149500
C	-4.69119300	1.00277600	-0.69742700
H	-5.01036800	1.25867300	-1.70780100
H	-5.48968400	0.47010400	-0.17483300
H	-4.44439200	1.91103100	-0.14151600
N	3.39519100	0.38174400	-0.04451200
C	3.76000300	1.78864100	0.09863100
H	3.32086400	2.38763100	-0.70491300
H	3.42142400	2.18181100	1.06217500
H	4.84331100	1.87762300	0.04666300
C	4.45529100	-0.60889500	-0.21035600
H	4.34843700	-1.13646700	-1.16324900
H	5.41712500	-0.09997700	-0.19926400
H	4.43574700	-1.33889600	0.60486900

1a (R = NH_2, R′ = OMe)
E(SMD/M06-2X/6-31G(d)) = -553.2652612 au
H(SMD/M06-2X/6-31G(d)) = -553.087067 au
G(SMD/M06-2X/6-31G(d)) = -553.137523 au
E(SMD/M06-2X/def2-TZVP//SMD/M06-2X /6-31G(d)) = -553.4947037 au

C	1.06515000	1.22689900	0.01894400
C	-0.20617300	0.66785100	0.02132900
C	-1.33968600	1.51817800	-0.21271500
C	-0.48746900	-0.72833900	0.23033000
C	-2.61862200	1.04330600	-0.24445400
H	-1.14329600	2.57503700	-0.36924000
C	-1.75630900	-1.22534400	0.20457700
H	0.34481200	-1.40236500	0.42018100

C	-2.85766000	-0.34758200	-0.03716400
H	-3.46525300	1.69918700	-0.42365900
H	-1.95721000	-2.28064600	0.36404700
C	2.19395500	0.36115500	0.29530100
O	2.66256600	0.16559100	1.40606700
O	2.76534600	-0.12084100	-0.83343400
C	3.97715600	-0.84925500	-0.63360900
H	4.31260800	-1.14635000	-1.62726800
H	4.73279200	-0.22218900	-0.15383100
H	3.80334100	-1.73471100	-0.01679900
N	-4.09950600	-0.82968900	-0.06765000
H	-4.89367600	-0.22495400	-0.23628700
H	-4.28291500	-1.81439800	0.08011700

1a (R = OMe, R′ = OMe)
E(SMD/M06-2X/6-31G(d)) = -612.3979685 au
H(SMD/M06-2X/6-31G(d)) = -612.202142 au
G(SMD/M06-2X/6-31G(d)) = -612.255301 au
E(SMD/M06-2X/def2-TZVP//SMD/M06-2X /6-31G(d)) = -612.6470602 au

C	-1.51471100	1.23378900	-0.00582000
C	-0.23490200	0.65916900	-0.04172700
C	0.88719900	1.50675800	0.18099900
C	0.02878300	-0.73048700	-0.26749600
C	2.17956300	1.03043100	0.19145700
H	0.69301300	2.56176600	0.34951100
C	1.30548200	-1.22262700	-0.26462300
H	-0.80569900	-1.40293400	-0.45045300
C	2.39280000	-0.34524000	-0.03122100
H	3.01220000	1.70040900	0.36703600
H	1.52172200	-2.27213600	-0.43390100
C	-2.64747100	0.36561100	-0.26948500
O	-3.11348400	0.18839100	-1.38273600
O	-3.20122300	-0.12700900	0.85566600
C	-4.41360900	-0.86179100	0.66390700
H	-4.73420700	-1.16750500	1.65938300
H	-5.17600800	-0.23279600	0.19837100
H	-4.23931100	-1.74050900	0.03834300
O	3.59140100	-0.91592800	-0.04090600
C	4.74662200	-0.10801600	0.19038400
H	5.59429200	-0.78948800	0.13728800
H	4.83975800	0.66130500	-0.58127400
H	4.70214100	0.35267900	1.18124800

1a (R = Me, R′ = OMe)
E(SMD/M06-2X/6-31G(d)) = -537.2100051 au
H(SMD/M06-2X/6-31G(d)) = -537.020774 au
G(SMD/M06-2X/6-31G(d)) = -537.072849 au
E(SMD/M06-2X/def2-TZVP//SMD/M06-2X /6-31G(d)) = -537.4243245 au

C	1.10125700	1.24122200	-0.00920700
C	-0.19537600	0.67440800	0.01143100
C	-1.29919400	1.53392500	-0.22690900
C	-0.46432200	-0.70617200	0.23684100

C	-2.59301400	1.04734900	-0.24606900
H	-1.09487800	2.58712900	-0.39460800
C	-1.75595500	-1.18549600	0.22082700
H	0.36409700	-1.38278600	0.43234300
C	-2.83742500	-0.31640600	-0.02474600
H	-3.42997300	1.71449600	-0.42948700
H	-1.95455900	-2.23902700	0.39699000
C	2.21684300	0.35693200	0.27552100
O	2.64537000	0.18392500	1.40377000
O	2.78895100	-0.14694000	-0.83084000
C	3.98719400	-0.89861600	-0.60713200
H	4.32679500	-1.21156600	-1.59376700
H	4.74561500	-0.27751500	-0.12524500
H	3.78379400	-1.77224200	0.01645600
C	-4.23413800	-0.86034300	-0.06718300
H	-4.97742600	-0.06309800	0.00031500
H	-4.39380200	-1.39650000	-1.01051300
H	-4.39944800	-1.57559800	0.74383400

1a (R = F, R′ = OMe)
E(SMD/M06-2X/6-31G(d)) = -597.116936 au
H(SMD/M06-2X/6-31G(d)) = -596.964697 au
G(SMD/M06-2X/6-31G(d)) = -597.014828 au
E(SMD/M06-2X/def2-TZVP//SMD/M06-2X /6-31G(d)) = -597.3648833 au

C	-1.04355200	1.23683900	0.01249800
C	0.24485300	0.65076300	0.01928800
C	0.48993000	-0.73792500	0.22343500
C	1.35785500	1.50380000	-0.21145200
C	1.77070900	-1.24621000	0.19722400
H	-0.34763900	-1.40426200	0.41155800
C	2.64722000	1.01064800	-0.24702700
H	1.16477300	2.56092900	-0.36310800
C	2.81981800	-0.35594900	-0.04071100
H	1.98785600	-2.29738200	0.35058500
H	3.51221200	1.63942700	-0.42485800
C	-2.17250600	0.36486800	0.28366200
O	-2.58999200	0.16941500	1.41208100
O	-2.76412400	-0.09690200	-0.82925900
C	-3.97468100	-0.83253700	-0.61477900
H	-4.32890200	-1.11098500	-1.60650800
H	-3.78151700	-1.72678300	-0.01789300
H	-4.71627100	-0.21069300	-0.10848700
F	4.05606000	-0.84401500	-0.07074400

1a (R = H, R′ = OMe)
E(SMD/M06-2X/6-31G(d)) = -497.9094392 au
H(SMD/M06-2X/6-31G(d)) = -497.74978 au
G(SMD/M06-2X/6-31G(d)) = -497.79772 au
E(SMD/M06-2X/def2-TZVP//SMD/M06-2X /6-31G(d)) = -498.1093296 au

C	-0.53374700	-1.19773300	0.03202800
C	0.70767900	-0.50613200	0.01638200
C	1.88000200	-1.27179900	-0.20823200

C	0.83870000	0.89852700	0.19376900
C	3.12507400	-0.66689300	-0.26055900
H	1.76826000	-2.34361300	-0.34047300
C	2.08252200	1.50006400	0.14887100
H	-0.05085800	1.49684900	0.37425400
C	3.21976300	0.71522800	-0.08029600
H	4.01933600	-1.25517300	-0.43643100
H	2.18278000	2.57143300	0.28737000
H	4.19502200	1.19223400	-0.11744800
C	-1.72727100	-0.41359000	0.29368900
O	-2.14978000	-0.23278100	1.42271200
O	-2.36192300	-0.02299500	-0.82179600
C	-3.62607600	0.61774900	-0.61077000
H	-4.00799500	0.84807400	-1.60454700
H	-4.31203100	-0.05184500	-0.08721900
H	-3.50077100	1.53572500	-0.03212500

1a (R = Cl, R′ = OMe)
E(SMD/M06-2X/6-31G(d)) = -957.4797063 au
H(SMD/M06-2X/6-31G(d)) = -957.328565 au
G(SMD/M06-2X/6-31G(d)) = -957.379658 au
E(SMD/M06-2X/def2-TZVP//SMD/M06-2X /6-31G(d)) = -957.7137662 au

C	-1.54210800	1.25389000	-0.01121100
C	-0.21649200	0.74167900	0.01117800
C	0.10754300	-0.62414400	0.23296100
C	0.84268700	1.65365100	-0.22324200
C	1.41859000	-1.05653200	0.22040500
H	-0.68836200	-1.33967700	0.42214300
C	2.16127600	1.23579300	-0.24447700
H	0.59233500	2.69651600	-0.38983100
C	2.42692700	-0.11628900	-0.02074100
H	1.67287300	-2.09692300	0.38977100
H	2.97323400	1.93076000	-0.42617100
C	-2.61485100	0.31643700	0.27065100
O	-3.01846600	0.12320400	1.40421200
O	-3.16992500	-0.20783800	-0.83088800
C	-4.32928000	-1.01940900	-0.60216900
H	-4.66046400	-1.34234200	-1.58819600
H	-4.07698500	-1.88516300	0.01419300
H	-5.11214900	-0.43786600	-0.11057900
Cl	4.07534600	-0.65639300	-0.04106200

1a (R = CF_3, R′ = OMe)
E(SMD/M06-2X/6-31G(d)) = -834.8426793 au
H(SMD/M06-2X/6-31G(d)) = -834.674392 au
G(SMD/M06-2X/6-31G(d)) = -834.732004 au
E(SMD/M06-2X/def2-TZVP//SMD/M06-2X /6-31G(d)) = -835.1964212 au

C	2.24955800	1.26878700	0.01123900
C	0.89272700	0.81960600	0.03123600
C	-0.10947600	1.78562500	-0.21144000
C	0.50422500	-0.52755600	0.25456700
C	-1.45085200	1.42997800	-0.24520800

H	0.19810700	2.81312100	-0.37612100
C	-0.82980800	-0.88499200	0.23343900
H	1.26270700	-1.27986400	0.45248100
C	-1.79263200	0.09933200	-0.02098600
H	-2.21949300	2.16974800	-0.43663600
H	-1.13549300	-1.91257900	0.40539100
C	3.27418800	0.27249900	0.26863700
O	3.65302400	0.03657500	1.40255000
O	3.81348300	-0.24835100	-0.83916800
C	4.93202800	-1.11991500	-0.62247400
H	5.25443800	-1.43417700	-1.61404000
H	5.73716000	-0.58704400	-0.11228600
H	4.63370200	-1.98682100	-0.02897600
C	-3.23828300	-0.32878600	-0.03101100
F	-3.43999000	-1.32664600	-0.90364300
F	-3.61157300	-0.78339400	1.17394300
F	-4.06546400	0.66830200	-0.35973000

1a (R = CN, R′ = OMe)
E(SMD/M06-2X/6-31G(d)) = -590.1212224 au
H(SMD/M06-2X/6-31G(d)) = -589.961193 au
G(SMD/M06-2X/6-31G(d)) = -590.013716 au
E(SMD/M06-2X/def2-TZVP//SMD/M06-2X /6-31G(d)) = -590.3552038 au

C	1.40916300	1.24493400	0.00000400
C	0.07511400	0.72517500	0.01803600
C	-0.97665100	1.64083200	-0.21483600
C	-0.23925900	-0.64064300	0.23310100
C	-2.29586800	1.21899600	-0.24305400
H	-0.72392200	2.68383900	-0.37438500
C	-1.55177700	-1.07383200	0.21633900
H	0.55909700	-1.35308100	0.42162700
C	-2.56918900	-0.13692400	-0.02482300
H	-3.10748800	1.91440300	-0.42475600
H	-1.80332800	-2.11557600	0.38126500
C	2.47964300	0.30013500	0.26490000
O	2.85663900	0.08957000	1.40433900
O	3.05174800	-0.20024000	-0.83435700
C	4.20723400	-1.01926900	-0.60324300
H	4.55108200	-1.32484700	-1.59023200
H	4.98317100	-0.44696300	-0.09062100
H	3.94304100	-1.89470000	-0.00617300
C	-3.93904400	-0.58567300	-0.04790800
N	-5.03784700	-0.94572800	-0.06681000

1a (R = NO_2, R′ = OMe)
E(SMD/M06-2X/6-31G(d)) = -702.3300564 au
H(SMD/M06-2X/6-31G(d)) = -702.165084 au
G(SMD/M06-2X/6-31G(d)) = -702.220659 au
E(SMD/M06-2X/def2-TZVP//SMD/M06-2X /6-31G(d)) = -702.6183971 au

C	1.84147100	1.26342300	0.08316200
C	0.49369600	0.77082900	0.07561100
C	-0.53467800	1.72001900	-0.11834500

C	0.14978700	-0.59532500	0.22797000
C	-1.86496400	1.33038200	-0.17358600
H	-0.25923700	2.76351900	-0.22786800
C	-1.17328800	-0.99933700	0.18978800
H	0.93008000	-1.33387000	0.38791100
C	-2.14233900	-0.02078300	-0.01422700
H	-2.66673300	2.04159000	-0.32711700
H	-1.45959200	-2.03702200	0.30691800
C	2.89309200	0.28105000	0.27520100
O	3.26271000	-0.01344300	1.39837900
O	3.45573600	-0.15182700	-0.85541900
C	4.59569000	-1.00723400	-0.68313100
H	4.93540200	-1.24677400	-1.68951900
H	5.38081100	-0.48750800	-0.13018800
H	4.31341800	-1.91818700	-0.15106000
N	-3.55865200	-0.44841900	-0.06111600
O	-3.79574300	-1.63052200	0.09816400
O	-4.39950000	0.40817300	-0.25561400

1′•B(C_6F_5)$_3$ (R = NMe_2, R′ = OMe)
E(SMD/M06-2X/6-31G(d)) = -2948.864545 au
H(SMD/M06-2X/6-31G(d)) = -2948.426868 au
G(SMD/M06-2X/6-31G(d)) = -2948.556604 au
E(SMD/M06-2X/def2-TZVP//SMD/M06-2X /6-31G(d)) = -2950.119982 au

C	2.25019000	-0.11552800	-0.80212200
N	1.65248400	-1.86038100	-0.83377900
N	1.59188800	-2.94865100	-0.68604000
C	3.64346900	-0.07473300	-0.88201200
C	4.44434700	-0.19548600	0.28698900
C	4.31098000	0.09539100	-2.12661900
C	5.80991100	-0.12327700	0.23173500
H	3.94864100	-0.33872300	1.24358100
C	5.67599200	0.17658800	-2.20073500
H	3.71471600	0.17562800	-3.03109000
C	6.47600200	0.07079300	-1.01964200
H	6.38441500	-0.20960500	1.14491600
H	6.14729400	0.32314900	-3.16414200
C	1.06143500	0.52761600	-0.93439600
O	-0.06441300	-0.08718300	-1.14563100
O	0.96572400	1.85038200	-0.81147500
C	2.06533300	2.59961400	-0.28802100
H	2.41297600	2.15735000	0.65068700
H	2.88418100	2.64209100	-1.01030000
H	1.67226000	3.59921600	-0.10869900
B	-1.20284800	-0.00869400	-0.13510500
C	-2.04353000	1.38742400	-0.32480300
C	-2.24519200	1.90366900	-1.60260600
C	-2.67993900	2.09501700	0.69047600
C	-2.98165800	3.05154700	-1.86385200
C	-3.42783800	3.24488100	0.47323500
C	-3.57790700	3.73091700	-0.81483000

C	-0.47710600	-0.20206000	1.33148900
C	-0.15657900	-1.46607800	1.82319700
C	0.07260300	0.84837800	2.06708100
C	0.60595300	-1.68725100	2.96414500
C	0.84064600	0.67411700	3.21062500
C	1.10562800	-0.60610400	3.66931900
C	-2.21708900	-1.24231600	-0.51000500
C	-3.40490800	-1.38036700	0.20185400
C	-2.02501800	-2.20246300	-1.50003400
C	-4.35197100	-2.36382700	-0.04028900
C	-2.95002400	-3.20315400	-1.77895900
C	-4.12237700	-3.28628100	-1.04799500
F	-2.59672200	1.69636400	1.96931600
F	-4.00518500	3.88503600	1.49498800
F	-4.29219900	4.83398800	-1.04222400
F	-3.12815700	3.49853900	-3.11530100
F	-1.73149500	1.28446700	-2.67746600
F	-3.67808400	-0.52736600	1.20416600
F	-5.47126600	-2.43154600	0.68545000
F	-5.01408400	-4.24325500	-1.30567100
F	-2.70920300	-4.09451600	-2.74541500
F	-0.91147800	-2.24168600	-2.24913100
F	-0.08686200	2.12447400	1.68203500
F	1.33767700	1.72879600	3.86158600
F	1.84229800	-0.79312700	4.76282300
F	0.86965500	-2.92955700	3.37520900
F	-0.54685000	-2.57956000	1.17767300
N	7.81519100	0.15343500	-1.08266700
C	8.61878600	0.06028700	0.13098900
H	9.66935400	0.16177500	-0.13435300
H	8.35840100	0.85779800	0.83456300
H	8.47485800	-0.90698300	0.62356900
C	8.48374000	0.34600300	-2.36453300
H	8.17956000	1.29063500	-2.82768500
H	9.55946400	0.37004600	-2.19970300
H	8.25499700	-0.47468500	-3.05198200

1′• $B(C_6F_5)_3$ (R = NH_2, R′ = OMe)
E(SMD/M06-2X/6-31G(d)) = -2870.292508 au
H(SMD/M06-2X/6-31G(d)) = -2869.915697 au
G(SMD/M06-2X/6-31G(d)) = -2870.036444 au
E(SMD/M06-2X/def2-TZVP//SMD/M06-2X /6-31G(d)) = -2871.524147 au

C	-2.51549900	-0.22363900	-1.30689500
N	-2.10560000	1.57393700	-1.32002000
N	-2.29571500	2.65647700	-1.25425000
C	-3.90426000	-0.35635600	-1.31722700
C	-4.64734300	-0.30935700	-0.10198900
C	-4.61938900	-0.51781500	-2.53786300
C	-6.00867200	-0.44276500	-0.09850700
H	-4.10823200	-0.17578400	0.83244600
C	-5.98092500	-0.65700400	-2.54804000

H	-4.06110500	-0.54043000	-3.46913700
C	-6.71000400	-0.62048000	-1.32579100
H	-6.57207300	-0.41959100	0.82911600
H	-6.52249500	-0.79547200	-3.47862100
C	-1.27989500	-0.77441200	-1.22222800
O	-0.16960300	-0.10256100	-1.33949900
O	-1.12037500	-2.08905200	-1.06190000
C	-2.22313900	-2.90894400	-0.67043000
H	-2.69851200	-2.50367800	0.22628000
H	-2.95173200	-2.99619400	-1.48064200
H	-1.78856800	-3.88485900	-0.45585600
B	0.80252800	0.09429200	-0.17763900
C	1.27720400	-1.33168100	0.47813500
C	1.70701500	-2.34729100	-0.37313300
C	1.41423000	-1.60808900	1.83171200
C	2.19107400	-3.56843000	0.06834900
C	1.90018800	-2.81692100	2.31922600
C	2.28887700	-3.80501000	1.43201200
C	-0.01725000	1.07778000	0.85610900
C	0.13603500	2.45608200	0.95425600
C	-1.10959500	0.57241700	1.55787300
C	-0.72440300	3.27625400	1.67721000
C	-1.99817900	1.34948100	2.28366100
C	-1.80776200	2.72172600	2.33822300
C	2.16513900	0.74576500	-0.79318500
C	3.13946200	1.18601300	0.09850900
C	2.53297000	0.79254700	-2.13169500
C	4.37934200	1.66865000	-0.28125300
C	3.77094100	1.26841600	-2.55803600
C	4.69828000	1.70933700	-1.63163600
F	1.07332300	-0.70323400	2.76361900
F	1.99872100	-3.02931500	3.63530600
F	2.75718000	-4.97014800	1.88101100
F	2.57563900	-4.51066900	-0.79842400
F	1.66640500	-2.16286000	-1.70116000
F	2.87366600	1.15893100	1.41695900
F	5.26542700	2.08819800	0.62639700
F	5.88641000	2.16653900	-2.02891600
F	4.07103700	1.29602000	-3.86048800
F	1.71251400	0.37711100	-3.10608900
F	-1.36210200	-0.75103400	1.54033700
F	-3.04143100	0.79342000	2.90890800
F	-2.64815800	3.49270400	3.02532300
F	-0.52331700	4.59475800	1.72558500
F	1.12802600	3.09488900	0.31493400
N	-8.04059100	-0.75268800	-1.32848400
H	-8.55295400	-0.87713900	-2.19204000
H	-8.57049500	-0.72491300	-0.46681100

1′•$B(C_6F_5)_3$ (R = OMe, R′ = OMe)

E(SMD/M06-2X/6-31G(d)) = -2929.425809 au

H(SMD/M06-2X/6-31G(d)) = -2929.031266 au
G(SMD/M06-2X/6-31G(d)) = -2929.15568 au
E(SMD/M06-2X/def2-TZVP//SMD/M06-2X /6-31G(d)) = -2930.678309 au

C	2.38143600	0.24598900	-1.15989500
N	1.92531300	-1.63667500	-1.23701300
N	2.03242600	-2.73065600	-1.21382500
C	3.77291000	0.30180400	-1.06186800
C	4.41651800	0.21878100	0.20629600
C	4.57042300	0.43885400	-2.22559300
C	5.78130100	0.29519800	0.29793200
H	3.80842200	0.10534000	1.09922900
C	5.94375500	0.52062100	-2.14324200
H	4.07873300	0.48890500	-3.19219000
C	6.55692500	0.44738400	-0.87557500
H	6.29506600	0.24488300	1.25171300
H	6.53527300	0.63793300	-3.04288100
C	1.14237100	0.78713400	-1.11374700
O	0.03876700	0.12194500	-1.29218500
O	0.98652900	2.10178400	-0.94571400
C	2.08719400	2.91082200	-0.52430100
H	2.52329500	2.51090800	0.39464900
H	2.84481600	2.97606400	-1.31002400
H	1.66109200	3.89596200	-0.33848500
B	-0.99068900	-0.08591300	-0.17879900
C	-1.48274900	1.33491100	0.47421800
C	-1.86773600	2.36389800	-0.38242100
C	-1.67678200	1.59583200	1.82389800
C	-2.36291700	3.58284600	0.05232500
C	-2.17555300	2.80220800	2.30452500
C	-2.51866400	3.80360300	1.41334900
C	-0.23029800	-1.09209100	0.87670200
C	-0.40687600	-2.46876900	0.95546700
C	0.83367600	-0.60806500	1.63440500
C	0.40551500	-3.30656800	1.71318900
C	1.67412400	-1.40295700	2.39726200
C	1.46254700	-2.77275400	2.43125600
C	-2.32747800	-0.71417600	-0.86917200
C	-3.34490800	-1.16073500	-0.03023500
C	-2.63469000	-0.73245800	-2.22350900
C	-4.57043800	-1.62474700	-0.47423000
C	-3.85598400	-1.18950900	-2.71380800
C	-4.82794900	-1.63814000	-1.83821200
F	-1.38280100	0.67730200	2.75812300
F	-2.33025000	2.99920700	3.61741500
F	-2.99820500	4.96654600	1.85569600
F	-2.70229100	4.53820600	-0.81877800
F	-1.76785800	2.19518300	-1.70942800
F	-3.13835800	-1.15909200	1.29895300
F	-5.50058400	-2.05163900	0.38442200
F	-6.00040500	-2.07762300	-2.29690400

F	-4.09762400	-1.19094900	-4.02855900
F	-1.76757400	-0.30526200	-3.15141600
F	1.10439200	0.71220600	1.64009400
F	2.69306000	-0.86554700	3.07658100
F	2.25819800	-3.56065400	3.15134100
F	0.18457900	-4.62236900	1.73941900
F	-1.37369300	-3.08897800	0.26205100
O	7.86868200	0.51425200	-0.68322600
C	8.72912700	0.66581000	-1.81367700
H	8.51042800	1.59834800	-2.34152800
H	9.73942800	0.69757000	-1.40900200
H	8.62681200	-0.18609000	-2.49176400

1′•B(C_6F_5)$_3$ (R = Me, R′ = OMe)
E(SMD/M06-2X/6-31G(d)) = -2854.238518 au
H(SMD/M06-2X/6-31G(d)) = -2853.850462 au
G(SMD/M06-2X/6-31G(d)) = -2853.973441 au
E(SMD/M06-2X/def2-TZVP//SMD/M06-2X /6-31G(d)) = -2855.457137 au

C	2.51329100	0.31198700	-1.34520900
N	2.08481400	-1.64044300	-1.35107800
N	2.15643700	-2.73591900	-1.30560200
C	3.91150100	0.37110700	-1.34121300
C	4.62581300	0.32247100	-0.11619200
C	4.62537000	0.49628300	-2.55776700
C	6.00108900	0.41770800	-0.11770900
H	4.07152800	0.21983300	0.81217300
C	6.00328900	0.59594600	-2.54187100
H	4.07241800	0.52012900	-3.49160900
C	6.70761800	0.55842600	-1.32726600
H	6.55177000	0.38662100	0.81782100
H	6.55317600	0.70168100	-3.47200100
C	1.26298100	0.81214500	-1.23518300
O	0.17370300	0.11189500	-1.33808000
O	1.08488900	2.12579000	-1.08069400
C	2.18973400	2.96437100	-0.73091100
H	2.68280100	2.58567100	0.16816000
H	2.90136700	3.03381500	-1.55850400
H	1.75360400	3.94305700	-0.53528700
B	-0.79867100	-0.09016500	-0.17137600
C	-1.29833100	1.33700900	0.46091400
C	-1.75086400	2.32844400	-0.40701200
C	-1.43620900	1.63500800	1.80978300
C	-2.25970100	3.54660100	0.01383300
C	-1.94640400	2.84188100	2.27697100
C	-2.35905700	3.80519100	1.37344000
C	0.03496700	-1.04354800	0.87745500
C	-0.10980500	-2.41918700	1.01645200
C	1.12161800	-0.51066800	1.56711500
C	0.75224300	-3.21126500	1.76858900
C	2.01062900	-1.25902700	2.32183700
C	1.82823300	-2.63004300	2.41814700

C	-2.14545400	-0.77364600	-0.78535700
C	-3.11851300	-1.21358100	0.10791300
C	-2.50383400	-0.84923000	-2.12507500
C	-4.34797700	-1.72252900	-0.27117400
C	-3.73105300	-1.35245400	-2.55085200
C	-4.65726500	-1.79196900	-1.62266800
F	-1.07246400	0.75491300	2.75638400
F	-2.04494900	3.07586600	3.58916800
F	-2.85073900	4.96775300	1.80295500
F	-2.66593800	4.46464900	-0.86852000
F	-1.70660000	2.12181700	-1.73186800
F	-2.86116900	-1.15974700	1.42712900
F	-5.23314500	-2.14018700	0.63800700
F	-5.83498100	-2.27538200	-2.01925700
F	-4.02185300	-1.40738200	-3.85441800
F	-1.68381300	-0.43662900	-3.10099900
F	1.36335400	0.81425000	1.51184000
F	3.04707100	-0.67695300	2.93439800
F	2.67015000	-3.37414100	3.13217900
F	0.56003900	-4.52896700	1.85438000
F	-1.09274300	-3.08420600	0.39068800
C	8.20051300	0.68971400	-1.30699800
H	8.47713900	1.70495300	-0.99769800
H	8.64342800	-0.00178300	-0.58463300
H	8.63210900	0.50612900	-2.29314400

1′•B(C_6F_5)$_3$ (R = F, R′ = OMe)
E(SMD/M06-2X/6-31G(d)) = -2914.144204 au
H(SMD/M06-2X/6-31G(d)) = -2913.79298 au
G(SMD/M06-2X/6-31G(d)) = -2913.9134 au
E(SMD/M06-2X/def2-TZVP//SMD/M06-2X /6-31G(d)) = -2915.396607 au

C	2.52266000	0.32946500	-1.34836000
N	2.09919600	-1.63651500	-1.35076200
N	2.15806900	-2.73255100	-1.30357100
C	3.92009400	0.38864200	-1.34738800
C	4.63264600	0.34921000	-0.12015300
C	4.62881600	0.50548500	-2.57039800
C	6.00807700	0.44465800	-0.11232900
H	4.07843000	0.25474900	0.80849500
C	6.00505500	0.60579700	-2.56939300
H	4.07217000	0.52405900	-3.50158500
C	6.66095400	0.57118900	-1.33923400
H	6.58768600	0.42749600	0.80349900
H	6.58174400	0.70740900	-3.48158800
C	1.26886700	0.82143700	-1.23434900
O	0.18582500	0.11343400	-1.33307100
O	1.08431900	2.13344800	-1.08161900
C	2.18624800	2.98043900	-0.74145700
H	2.68409500	2.61038900	0.15860100
H	2.89405800	3.04820100	-1.57248500
H	1.74563000	3.95798600	-0.55075500

B	-0.78843300	-0.09141600	-0.16625900
C	-1.29276700	1.33468500	0.46340900
C	-1.75102900	2.32205600	-0.40612600
C	-1.42950100	1.63510600	1.81183700
C	-2.26490200	3.53874900	0.01267700
C	-1.94462300	2.84064800	2.27702800
C	-2.36336700	3.79985200	1.37190000
C	0.04873000	-1.04071900	0.88300900
C	-0.09363900	-2.41606600	1.02732200
C	1.13574100	-0.50360800	1.56875000
C	0.77104400	-3.20383500	1.78108900
C	2.02753400	-1.24760700	2.32444200
C	1.84717200	-2.61850400	2.42669300
C	-2.13102100	-0.78058100	-0.78203900
C	-3.10426600	-1.22190000	0.11033200
C	-2.48629700	-0.86036000	-2.12230500
C	-4.33109100	-1.73611100	-0.27014500
C	-3.71074500	-1.36902300	-2.54947900
C	-4.63726100	-1.80974000	-1.62214800
F	-1.05975200	0.75884200	2.75956600
F	-2.04197100	3.07725000	3.58875700
F	-2.85975200	4.96100200	1.79951900
F	-2.67658900	4.45287500	-0.87108600
F	-1.70681000	2.11257800	-1.73057600
F	-2.84943200	-1.16423700	1.42983800
F	-5.21660000	-2.15487900	0.63808500
F	-5.81228900	-2.29838100	-2.02004900
F	-3.99858800	-1.42796700	-3.85347400
F	-1.66561300	-0.44670700	-3.09725300
F	1.37496700	0.82172800	1.50786700
F	3.06443100	-0.66146200	2.93245200
F	2.69145600	-3.35848400	3.14199300
F	0.58111100	-4.52136400	1.87216000
F	-1.07624000	-3.08508400	0.40566300
F	7.98473400	0.66529200	-1.33392300

1′•B(C_6F_5)$_3$ (R = H, R′ = OMe)
E(SMD/M06-2X/6-31G(d)) = -2814.937673 au
H(SMD/M06-2X/6-31G(d)) = -2814.579064 au
G(SMD/M06-2X/6-31G(d)) = -2814.699649 au
E(SMD/M06-2X/def2-TZVP//SMD/M06-2X /6-31G(d)) = -2816.142177 au

C	2.63574400	0.41831600	-1.52252700
N	2.28801100	-1.58198600	-1.46136400
N	2.38416400	-2.67460400	-1.40038700
C	4.03376900	0.51158200	-1.56917200
C	4.78419500	0.50980400	-0.36674500
C	4.69366700	0.63083800	-2.81644400
C	6.15949300	0.64387300	-0.41729400
H	4.26144200	0.41295300	0.57988800
C	6.07027700	0.77030800	-2.85335900
H	4.10229000	0.62099400	-3.72648400

C	6.79504200	0.77424900	-1.65750900
H	6.74355800	0.65123300	0.49642000
H	6.58541100	0.87458200	-3.80195900
C	1.37235100	0.86435800	-1.34962900
O	0.31164400	0.11766600	-1.38476300
O	1.15012500	2.17293100	-1.21376500
C	2.23920900	3.06234800	-0.94689100
H	2.78893400	2.73300000	-0.06121500
H	2.90630600	3.12799500	-1.81125900
H	1.77704300	4.03101000	-0.76217100
B	-0.60359300	-0.09776300	-0.17319600
C	-1.11725600	1.32079100	0.46668500
C	-1.64561700	2.28548200	-0.38843800
C	-1.19897700	1.63072800	1.81752600
C	-2.17513200	3.49071000	0.04381300
C	-1.72701400	2.82530900	2.29621900
C	-2.21650700	3.76258900	1.40385200
C	0.30375400	-1.01364800	0.84620600
C	0.20321300	-2.39013600	1.01225700
C	1.40509900	-0.44016000	1.47733200
C	1.11841100	-3.14542900	1.73919600
C	2.34637300	-1.15096600	2.20433900
C	2.20471300	-2.52428600	2.33225300
C	-1.95484500	-0.82674000	-0.72048600
C	-2.87399400	-1.28498300	0.21942200
C	-2.36965200	-0.92637100	-2.04222100
C	-4.10303400	-1.83444500	-0.09985700
C	-3.59859700	-1.47061600	-2.40819300
C	-4.46915400	-1.92765800	-1.43562600
F	-0.76027500	0.77492200	2.75451900
F	-1.76875700	3.07229200	3.60897700
F	-2.72590600	4.91310900	1.84471200
F	-2.65557500	4.38333200	-0.82711100
F	-1.65895000	2.06344200	-1.71146600
F	-2.56094500	-1.20799700	1.52532700
F	-4.93462900	-2.26771700	0.85155900
F	-5.64799000	-2.45012400	-1.77514900
F	-3.94525100	-1.54820000	-3.69679300
F	-1.60646800	-0.49838300	-3.05693700
F	1.60762100	0.88990200	1.38884400
F	3.39071000	-0.53016200	2.76273300
F	3.09580900	-3.23313200	3.02199100
F	0.96695500	-4.46604700	1.85469800
F	-0.78687800	-3.09172400	0.44037500
H	7.87516700	0.88168700	-1.69152800

1′•B(C_6F_5)$_3$ (R = Cl, R′ = OMe)
E(SMD/M06-2X/6-31G(d)) = -3274.506638 au
H(SMD/M06-2X/6-31G(d)) = -3274.156807 au
G(SMD/M06-2X/6-31G(d)) = -3274.279654 au
E(SMD/M06-2X/def2-TZVP//SMD/M06-2X /6-31G(d)) = -3275.745395 au

C	2.31278300	0.28016300	-1.43998800
N	1.84307000	-1.69779500	-1.38865000
N	1.82356000	-2.79522200	-1.42881000
C	3.70912700	0.29624500	-1.30608500
C	4.30712100	0.38472200	-0.02478500
C	4.52830900	0.23039600	-2.45847000
C	5.68170300	0.43031000	0.10005100
H	3.67415500	0.43123400	0.85519200
C	5.90454900	0.26863100	-2.33898300
H	4.06112300	0.15353400	-3.43488500
C	6.46053200	0.36841900	-1.06025100
H	6.15363900	0.51011100	1.07280600
H	6.54554300	0.22527500	-3.21225900
C	1.06568200	0.78979400	-1.29620200
O	-0.02201400	0.08716200	-1.35845900
O	0.89205700	2.10709700	-1.20944500
C	2.00764300	2.96578100	-0.94793500
B	-0.98162300	-0.08713400	-0.17529700
C	-1.48616600	1.35925100	0.40647400
C	-1.95332300	2.31306700	-0.49524100
C	-1.61435800	1.70892500	1.74401000
C	-2.46797300	3.54295700	-0.11747000
C	-2.12967000	2.92913900	2.16844300
C	-2.55752400	3.85358800	1.23178900
C	-0.13214900	-0.99322100	0.90126600
C	-0.27002500	-2.36170600	1.10447400
C	0.94817900	-0.42384100	1.56994700
C	0.58748100	-3.11083600	1.90469200
C	1.83160700	-1.12790600	2.37182500
C	1.65168000	-2.49248100	2.53871000
C	-2.32433500	-0.80535500	-0.75516200
C	-3.28814000	-1.21868300	0.16054100
C	-2.68753800	-0.93951100	-2.08897600
C	-4.51366100	-1.75586400	-0.19133500
C	-3.91098500	-1.47307100	-2.48758400
C	-4.82809700	-1.88397700	-1.53735700
F	-1.23586600	0.86871900	2.72051600
F	-2.21836600	3.21300900	3.47127600
F	-3.05415300	5.02824100	1.62008400
F	-2.88912900	4.42233300	-1.03131900
F	-1.92092300	2.05499700	-1.81133900
F	-3.02480900	-1.10784000	1.47500400
F	-5.39018800	-2.14511200	0.73840300
F	-6.00190300	-2.39635400	-1.90778800
F	-4.20669500	-1.58585200	-3.78616400
F	-1.87584300	-0.55857200	-3.08460300
F	1.19250600	0.89721800	1.44180200
F	2.85863800	-0.50875300	2.96374900
F	2.48801300	-3.19499800	3.29934000
F	0.40104100	-4.42360300	2.05269200

F	-1.24207500	-3.06156300	0.50094300
H	2.70869300	2.95534900	-1.78749400
H	1.58035200	3.96108400	-0.83629700
H	2.50802500	2.66689000	-0.02347400
Cl	8.18428800	0.41781200	-0.90549400

1′•B(C_6F_5)$_3$ (R = CF_3, R′ = OMe)
E(SMD/M06-2X/6-31G(d)) = -3151.869293 au
H(SMD/M06-2X/6-31G(d)) = -3151.502289 au
G(SMD/M06-2X/6-31G(d)) = -3151.631129 au
E(SMD/M06-2X/def2-TZVP//SMD/M06-2X /6-31G(d)) = -3153.227888 au

C	1.98998900	0.26558100	-1.33648300
N	1.49289100	-1.74273200	-1.31416700
N	1.41719100	-2.83746000	-1.35457400
C	3.38562900	0.24295000	-1.16049700
C	3.93984400	0.31050900	0.13810100
C	4.23215500	0.16568700	-2.29147000
C	5.31535200	0.32040400	0.30171700
H	3.27883500	0.36723300	0.99638000
C	5.60460200	0.16954600	-2.12227400
H	3.78979500	0.10679000	-3.28034100
C	6.12896400	0.24650500	-0.82862200
H	5.75271300	0.37974500	1.29167900
H	6.26936300	0.11393200	-2.97842700
C	0.74289900	0.78187400	-1.22430600
O	-0.34529700	0.08689000	-1.32343800
O	0.58145900	2.09972400	-1.13307700
C	1.70133200	2.94270700	-0.83630700
B	-1.34634800	-0.08243000	-0.17157500
C	-1.86366300	1.36740400	0.38880100
C	-2.29325600	2.32227700	-0.53034100
C	-2.03623100	1.72005700	1.72057400
C	-2.81357400	3.55573300	-0.17251200
C	-2.55894100	2.94389400	2.12493500
C	-2.94834900	3.86915900	1.17232800
C	-0.53606400	-0.98849400	0.93406800
C	-0.68638800	-2.35569000	1.13754700
C	0.52376400	-0.42106800	1.63613400
C	0.14063000	-3.10515800	1.96894000
C	1.37738900	-1.12576500	2.46905900
C	1.18613100	-2.48887600	2.63529700
C	-2.66999400	-0.79735000	-0.79633700
C	-3.66457900	-1.20781700	0.08713300
C	-2.98943500	-0.92992300	-2.14141500
C	-4.87974900	-1.74044900	-0.30505700
C	-4.20119600	-1.45852200	-2.58025700
C	-5.15045000	-1.86642900	-1.66076900
F	-1.69599500	0.87940200	2.71059000
F	-2.69168700	3.23053800	3.42331200
F	-3.45118000	5.04737700	1.54104600
F	-3.19656100	4.43638600	-1.10174300

F	-2.21424000	2.06244500	-1.84422100
F	-3.44381600	-1.09809500	1.40940400
F	-5.78750400	-2.12721300	0.59519700
F	-6.31328900	-2.37421800	-2.06978200
F	-4.45477300	-1.56927300	-3.88785000
F	-2.14360200	-0.55194400	-3.10934700
F	0.77840100	0.89829600	1.51057900
F	2.38863500	-0.50890200	3.08979700
F	1.99442500	-3.19192600	3.42505700
F	-0.05610800	-4.41654100	2.11515600
F	-1.63961500	-3.05402700	0.50320000
C	7.63148800	0.26827100	-0.68538600
F	8.14049900	1.40045300	-1.19018600
F	8.19221700	-0.74755600	-1.35420400
F	8.02205700	0.18129400	0.58897300
H	2.42704500	2.92294400	-1.65476700
H	1.28531200	3.94389000	-0.73657400
H	2.16822400	2.63546500	0.10281300

1′•B(C_6F_5)$_3$ (R = CN, R′ = OMe)
E(SMD/M06-2X/6-31G(d)) = -2907.147285 au
H(SMD/M06-2X/6-31G(d)) = -2906.788609 au
G(SMD/M06-2X/6-31G(d)) = -2906.913566 au
E(SMD/M06-2X/def2-TZVP//SMD/M06-2X /6-31G(d)) = -2908.386191 au

C	2.36479400	0.31866800	-1.53996100
N	1.91470700	-1.70383900	-1.42348300
N	1.84730200	-2.79983700	-1.42792500
C	3.76631700	0.32614300	-1.39143300
C	4.35122300	0.44940700	-0.10927300
C	4.58428800	0.21768700	-2.53885700
C	5.72725000	0.48282500	0.02089200
H	3.71261400	0.53303900	0.76308400
C	5.96189800	0.24550800	-2.41148700
H	4.11781600	0.11561900	-3.51295400
C	6.51917700	0.37779300	-1.13216200
H	6.19444100	0.58588600	0.99369400
H	6.60644000	0.16857500	-3.27964100
C	1.11332200	0.80751000	-1.34634900
O	0.04168200	0.08323500	-1.37074900
O	0.92286500	2.12096700	-1.27203700
C	2.03562200	2.99983300	-1.06335800
H	2.56634900	2.72772900	-0.14755500
H	2.70905500	2.97916100	-1.92519700
H	1.59895600	3.99181300	-0.96084300
B	-0.90253100	-0.08665000	-0.17073500
C	-1.41478700	1.36227600	0.39484000
C	-1.91244500	2.29312400	-0.51454500
C	-1.51978300	1.73484600	1.72813800
C	-2.43634200	3.52266200	-0.14881100
C	-2.04312400	2.95569400	2.14067700
C	-2.50269400	3.85674100	1.19624900

C	-0.02576800	-0.96744100	0.90384300
C	-0.14834200	-2.33280500	1.13564600
C	1.06209400	-0.37689300	1.54064600
C	0.72999700	-3.05869500	1.93475700
C	1.96625800	-1.05713700	2.33987900
C	1.80017800	-2.41942800	2.53727000
C	-2.24131900	-0.83082500	-0.72409700
C	-3.19028200	-1.23681200	0.21017500
C	-2.61537800	-0.99814200	-2.05103900
C	-4.41190700	-1.79742300	-0.11774600
C	-3.83537800	-1.55625300	-2.42573800
C	-4.73760700	-1.95831900	-1.45757500
F	-1.10903300	0.91785800	2.71109400
F	-2.10844500	3.26268600	3.43945700
F	-3.00683400	5.03168900	1.57324700
F	-2.88704400	4.37957000	-1.06962200
F	-1.90013700	2.01175500	-1.82627800
F	-2.91444600	-1.09494800	1.51903200
F	-5.27388700	-2.17813700	0.82879500
F	-5.90795300	-2.49379300	-1.80520900
F	-4.14227700	-1.70152700	-3.71834800
F	-1.81789800	-0.62788200	-3.06211100
F	1.29334400	0.94351100	1.37924200
F	2.99816800	-0.41767800	2.90030300
F	2.65538800	-3.10013800	3.29636400
F	0.55785500	-4.36961000	2.11145000
F	-1.12371800	-3.05259800	0.56228500
C	7.95379800	0.40741500	-0.99651500
N	9.10463400	0.43128200	-0.88806800

1′•B(C_6F_5)$_3$ (R = NO_2, R′ = OMe)
E(SMD/M06-2X/6-31G(d)) = -3019.355577 au
H(SMD/M06-2X/6-31G(d)) = -3018.991809 au
G(SMD/M06-2X/6-31G(d)) = -3019.118271 au
E(SMD/M06-2X/def2-TZVP//SMD/M06-2X /6-31G(d)) = -3020.648867 au

C	2.14911900	0.28369000	-1.56053200
N	1.67805800	-1.74911200	-1.41586700
N	1.58743700	-2.84318000	-1.43834900
C	3.54297300	0.27287400	-1.32925500
C	4.06047000	0.46498400	-0.02774700
C	4.41745700	0.08376000	-2.42238700
C	5.42992400	0.48943900	0.17549200
H	3.37952100	0.61142400	0.80313500
C	5.78874500	0.09872300	-2.22484300
H	4.00250400	-0.06903200	-3.41292200
C	6.25248800	0.30380600	-0.93049300
H	5.85491700	0.64422000	1.15947900
H	6.48443700	-0.03927800	-3.04302200
C	0.89954800	0.78768700	-1.36139700
O	-0.17400200	0.07006800	-1.36454000
O	0.71874600	2.10194600	-1.33317000

C	1.84691900	2.98315800	-1.24556500
H	2.40417700	2.79752700	-0.32414100
H	2.49168600	2.86498800	-2.12154000
H	1.42396600	3.98589100	-1.23008700
B	-1.12457500	-0.08955600	-0.16570800
C	-1.61256200	1.36529500	0.40434900
C	-2.09809700	2.30703800	-0.50032200
C	-1.71039700	1.73367600	1.73962700
C	-2.60268200	3.54296100	-0.12845500
C	-2.21456800	2.96034000	2.15809600
C	-2.66190600	3.87221100	1.21801400
C	-0.26033500	-0.98728700	0.90330100
C	-0.39953000	-2.35173500	1.12974700
C	0.83660100	-0.41296000	1.53871500
C	0.47279200	-3.09131000	1.92304400
C	1.73510600	-1.10635500	2.33272400
C	1.55216200	-2.46737200	2.52533400
C	-2.47231800	-0.81070100	-0.72602600
C	-3.42706900	-1.21275000	0.20401300
C	-2.84844600	-0.95859100	-2.05478000
C	-4.65656400	-1.75224400	-0.13000900
C	-4.07621600	-1.49503600	-2.43539400
C	-4.98422600	-1.89440400	-1.47148200
F	-1.31130100	0.90608600	2.71829100
F	-2.27360200	3.26261900	3.45813900
F	-3.14764700	5.05281600	1.60087500
F	-3.04239300	4.41003100	-1.04488400
F	-2.09405700	2.03017600	-1.81290500
F	-3.14985300	-1.08750900	1.51423700
F	-5.52424800	-2.13012300	0.81234400
F	-6.16204000	-2.40914100	-1.82477700
F	-4.38496100	-1.62166900	-3.72944300
F	-2.04578400	-0.58858200	-3.06177600
F	1.08352800	0.90552200	1.37809200
F	2.77512500	-0.48153500	2.89401100
F	2.40101700	-3.16153600	3.27910500
F	0.28574500	-4.40073000	2.09443000
F	-1.38512300	-3.05658900	0.55574700
N	7.71801400	0.32823000	-0.71521000
O	8.43033100	0.19330400	-1.69061300
O	8.11506400	0.48200100	0.42303100

1a•B(C_6F_5)$_3$ (R = NMe_2, R′ = OMe)
E(SMD/M06-2X/6-31G(d)) = -2839.407118 au
H(SMD/M06-2X/6-31G(d)) = -2838.978879 au
G(SMD/M06-2X/6-31G(d)) = -2839.10378 au
E(SMD/M06-2X/def2-TZVP//SMD/M06-2X /6-31G(d)) = -2840.619045 au

C	2.16842700	0.26659400	1.93383300
C	3.46881500	0.07441400	1.66489900
C	4.37560400	-0.46232400	2.66040600
C	4.03663900	0.39350800	0.36710500

C	5.69145900	-0.65233900	2.40466200
H	3.96631800	-0.71138100	3.63459400
C	5.34975700	0.20791300	0.09661100
H	3.36981100	0.78842000	-0.39247100
C	6.23756700	-0.32204900	1.10726400
H	6.33645800	-1.05410700	3.17517600
H	5.73679200	0.45651200	-0.88290500
C	0.90898300	0.62841300	1.63507400
O	0.02112100	-0.26271900	1.28263600
O	0.43650900	1.85999800	1.86878700
C	1.41154000	2.89004100	2.01659800
H	2.04752600	2.69667900	2.88749000
H	2.02649700	2.96392300	1.11396400
H	0.85247400	3.81324100	2.16513100
B	-1.05356100	-0.17922600	0.21726700
C	-1.31313700	1.34201500	-0.36316900
C	-0.23825400	2.05472200	-0.88695400
C	-2.53817900	1.98281800	-0.49812100
C	-0.32773700	3.32051100	-1.43909800
C	-2.68398500	3.24781500	-1.06095600
C	-1.57321700	3.92460300	-1.53192100
C	-2.34703800	-0.82311500	0.99312900
C	-2.92174900	-2.06943200	0.78710600
C	-2.87475800	-0.11448800	2.07052100
C	-3.95942400	-2.57319900	1.56716600
C	-3.90373400	-0.57453400	2.87428800
C	-4.45271700	-1.82314900	2.61846500
C	-0.58097900	-1.02597400	-1.10861900
C	-1.49717700	-1.23370800	-2.13553300
C	0.70805000	-1.45892300	-1.38453000
C	-1.19164800	-1.85109400	-3.33621000
C	1.06384300	-2.08072200	-2.57819400
C	0.10936000	-2.28094600	-3.55899200
F	-3.67778700	1.40835700	-0.08352300
F	-3.89162600	3.81158400	-1.15376700
F	-1.69586500	5.13910700	-2.06776800
F	0.76046500	3.95366200	-1.88805900
F	0.99784400	1.51211100	-0.84404900
F	-2.76794900	-0.82561800	-1.96863600
F	-2.12231400	-2.03186400	-4.27783300
F	0.43463200	-2.87510600	-4.70823100
F	2.32410700	-2.47725500	-2.78680800
F	1.71094500	-1.28468100	-0.50630700
F	-2.38892000	1.10328000	2.35840000
F	-4.37120000	0.16253000	3.88675500
F	-5.44285000	-2.29212600	3.37874900
F	-4.47131200	-3.78217400	1.31527000
F	-2.49664300	-2.88786100	-0.19005600
N	7.52349000	-0.49982400	0.84865400
C	8.43037500	-1.03244000	1.86974300

H	9.43149400	-1.09192900	1.44951800
H	8.45008700	-0.37356600	2.74185300
H	8.11383700	-2.03301700	2.17632400
C	8.07938000	-0.16851100	-0.46696700
H	7.94222700	0.89436500	-0.68228800
H	9.14323200	-0.39327000	-0.46170000
H	7.59544100	-0.76324600	-1.24585900

1a•B(C_6F_5)$_3$ (R = NH_2, R′ = OMe)
E(SMD/M06-2X/6-31G(d)) = -2760.832245 au
H(SMD/M06-2X/6-31G(d)) = -2760.464661 au
G(SMD/M06-2X/6-31G(d)) = -2760.582727 au
E(SMD/M06-2X/def2-TZVP//SMD/M06-2X /6-31G(d)) = -2762.019963 au

C	-2.44921400	0.70193000	-2.03483700
C	-3.75195000	0.40257200	-1.90203700
C	-4.57486100	0.11887400	-3.06282100
C	-4.39406300	0.34553300	-0.59924300
C	-5.88867000	-0.18682200	-2.94548500
H	-4.10098000	0.15752700	-4.03859900
C	-5.70678900	0.04174200	-0.47017200
H	-3.78370100	0.55191100	0.27381200
C	-6.49293500	-0.23574400	-1.64200700
H	-6.50558000	-0.40170000	-3.81250800
H	-6.18920300	-0.00329400	0.50121300
C	-1.23745500	1.00229700	-1.52629900
O	-0.34247500	0.07053600	-1.35457800
O	-0.81230900	2.26093100	-1.37193100
C	-1.82456200	3.26568400	-1.32558200
H	-2.37798200	3.29752300	-2.27053800
H	-2.51176200	3.07450100	-0.49542000
H	-1.30329500	4.20945000	-1.17009000
B	0.67509600	-0.10761700	-0.24307200
C	0.81062200	1.18928800	0.76403600
C	-0.33444000	1.68514200	1.38054900
C	1.98751500	1.80219400	1.17510400
C	-0.35169800	2.73960400	2.27649500
C	2.02543700	2.85636300	2.08358100
C	0.85005800	3.33140900	2.63741600
C	2.04034900	-0.44440300	-1.08569400
C	2.65120300	-1.68154600	-1.23629600
C	2.59937700	0.57332900	-1.85552200
C	3.75062500	-1.89820600	-2.06268400
C	3.69093600	0.40578300	-2.69068400
C	4.27387900	-0.84904100	-2.79564500
C	0.18167600	-1.31906900	0.74890400
C	1.06291100	-1.80442700	1.71079400
C	-1.10379600	-1.83618100	0.82440300
C	0.73036600	-2.75717500	2.65835100
C	-1.48573300	-2.79194700	1.76190000
C	-0.56419400	-3.25796900	2.68195500
F	3.18136200	1.40812600	0.70674900

F	3.19120600	3.40955000	2.42922000
F	0.87025000	4.34194500	3.50657200
F	-1.50040400	3.17951400	2.79916700
F	-1.53298400	1.13579100	1.08614600
F	2.32417000	-1.33766000	1.73461900
F	1.62803800	-3.19197000	3.54735800
F	-0.91576900	-4.17297500	3.58659000
F	-2.74012100	-3.25520300	1.78571300
F	-2.07735800	-1.42475200	-0.00671400
F	2.08010800	1.80959600	-1.79020400
F	4.18534800	1.42968600	-3.39340200
F	5.32454200	-1.03871400	-3.59448600
F	4.29363600	-3.11556400	-2.16261900
F	2.20196700	-2.77011700	-0.58951100
N	-7.77099600	-0.53462600	-1.51974200
H	-8.21640500	-0.57793500	-0.60886400
H	-8.34480600	-0.73985000	-2.33105300

1a•$B(C_6F_5)_3$ (R = OMe, R′ = OMe)
E(SMD/M06-2X/6-31G(d)) = -2819.959076 au
H(SMD/M06-2X/6-31G(d)) = -2819.573768 au
G(SMD/M06-2X/6-31G(d)) = -2819.692595 au
E(SMD/M06-2X/def2-TZVP//SMD/M06-2X /6-31G(d)) = -2821.16845 au

C	2.30444100	0.71816200	-1.38715600
C	3.61805400	0.44959900	-1.27940200
C	4.31871300	0.63646500	-0.02830800
C	4.36901300	-0.02082300	-2.41813100
C	5.64813900	0.37311400	0.06620800
H	3.76375500	1.01518200	0.82388100
C	5.70228100	-0.29637500	-2.32616500
H	3.84053900	-0.15899200	-3.35597900
C	6.35521900	-0.10001500	-1.07795500
H	6.20394300	0.51205300	0.98680700
H	6.25188700	-0.65496200	-3.18764800
C	1.00494200	0.98100400	-1.48414100
O	0.11923200	0.01664100	-1.39887000
O	0.52221900	2.20863100	-1.71239000
C	1.47641900	3.26900200	-1.68299900
H	1.98911000	3.28837600	-0.71570300
H	2.20549700	3.15325100	-2.49257500
H	0.91048600	4.18843700	-1.82575600
B	-0.80185600	-0.12960700	-0.20035500
C	-1.71991200	1.21634000	0.02228800
C	-2.34706800	1.78436800	-1.08447100
C	-2.04426500	1.80366900	1.23836800
C	-3.19084700	2.88195600	-1.01399100

C	-2.88879000	2.90231200	1.35659200
C	-3.46400500	3.44798000	0.22236400
C	0.18334100	-0.48440300	1.06730400
C	0.41074600	-1.75478000	1.58427000
C	0.98381500	0.50942400	1.62364500
C	1.33844400	-2.02565400	2.58487000
C	1.92463100	0.28656700	2.61589200
C	2.11069300	-0.99908100	3.09936400
C	-1.87824100	-1.30383300	-0.56398100
C	-2.75087600	-1.72436900	0.43615000
C	-2.11754000	-1.85425200	-1.81674800
C	-3.77149200	-2.63887700	0.24450400
C	-3.13411100	-2.77688000	-2.05460900
C	-3.96501000	-3.17202100	-1.02230900
F	-1.54577100	1.33198600	2.39305700
F	-3.15332100	3.43172400	2.55549400
F	-4.27642300	4.50154300	0.31707800
F	-3.75242500	3.38941700	-2.11618200
F	-2.15315500	1.25956100	-2.30389200
F	-2.60469800	-1.22858300	1.67841200
F	-4.56813600	-3.00730000	1.25198500
F	-4.94246400	-4.05316400	-1.23988200
F	-3.31597600	-3.27984500	-3.28013000
F	-1.37900000	-1.52927000	-2.88672400
F	0.86714700	1.78249500	1.19852500
F	2.68751500	1.28839900	3.07006300
F	3.01997700	-1.24102900	4.04275700
F	1.50428100	-3.27166400	3.03659600
F	-0.26516800	-2.82262100	1.13126200
O	7.62948600	-0.32636300	-0.88480000
C	8.45241600	-0.79605700	-1.96521300
H	8.45386900	-0.06819300	-2.77965600
H	9.45068500	-0.89068200	-1.54317400
H	8.09567700	-1.76779800	-2.31317000

1a•B(C_6F_5)$_3$ (R = Me, R′ = OMe)
E(SMD/M06-2X/6-31G(d)) = -2744.766292 au
H(SMD/M06-2X/6-31G(d)) = -2744.390193 au
G(SMD/M06-2X/6-31G(d)) = -2744.504611 au
E(SMD/M06-2X/def2-TZVP//SMD/M06-2X /6-31G(d)) = -2745.941309 au

C	2.93957000	-0.93308800	-0.87837300
C	4.21636600	-0.52457400	-0.56130900
C	5.20115500	-1.51082400	-0.23679300
C	4.60144400	0.85478400	-0.54339800

C	6.48230700	-1.13746300	0.08746300
H	4.90325400	-2.55439100	-0.25151200
C	5.88860500	1.21333500	-0.22073400
H	3.85798400	1.60917700	-0.78534200
C	6.84372300	0.22650100	0.09954300
H	7.23006200	-1.88392000	0.33752700
H	6.18228000	2.25798000	-0.20647800
C	1.75508100	-0.26003900	-1.14567700
O	1.05044100	0.15032200	-0.15884600
O	1.26633000	-0.15485700	-2.36272300
C	2.03494400	-0.74524600	-3.42278800
H	3.00238400	-0.24528600	-3.51748300
H	2.17072900	-1.81270200	-3.22409500
H	1.44886400	-0.59660700	-4.32784900
B	-0.46709800	0.14866100	0.09824700
C	-1.33675200	-0.46686300	-1.14723800
C	-1.00078300	-1.71930200	-1.65375600
C	-2.49164700	0.08328300	-1.68829500
C	-1.70465400	-2.37157700	-2.65157500
C	-3.23804700	-0.53821300	-2.68468900
C	-2.84304500	-1.77200300	-3.17096300
C	-0.76455400	1.72343900	0.41780200
C	-1.07667600	2.28919600	1.64657800
C	-0.60640500	2.63975300	-0.61936300
C	-1.24576500	3.65793900	1.83618300
C	-0.76216800	4.00801100	-0.47770300
C	-1.08692000	4.52324300	0.76918900
C	-0.74604900	-0.86920600	1.34691300
C	-2.03535200	-0.92779700	1.86851900
C	0.14240600	-1.79494000	1.87673500
C	-2.42938900	-1.80262700	2.86549500
C	-0.21051100	-2.69487100	2.87912600
C	-1.50091300	-2.69895600	3.37713900
F	-2.96167400	1.26769600	-1.26947900
F	-4.33678300	0.04496000	-3.17073600
F	-3.54661200	-2.37801700	-4.12659400
F	-1.31077000	-3.56410700	-3.10621500
F	0.08845600	-2.35212100	-1.17443400
F	-2.96631900	-0.08053000	1.39508500
F	-3.68042200	-1.80005100	3.33301200
F	-1.85233000	-3.55584700	4.33607100
F	0.68794200	-3.56075700	3.35780500
F	1.41073700	-1.88585100	1.44357400
F	-0.29661600	2.19354700	-1.84774100
F	-0.60228900	4.83117600	-1.51771400
F	-1.23963400	5.83643000	0.93541700
F	-1.54785200	4.14116900	3.04430000
F	-1.22714900	1.53777900	2.74852400
C	8.24337300	0.60982600	0.45873000
H	8.47011400	0.27312300	1.47681900

H	8.95295200	0.10768900	-0.20813800
H	8.39845800	1.68829900	0.39943200

1a•B(C_6F_5)$_3$ (R = F, R′ = OMe)
E(SMD/M06-2X/6-31G(d)) = -2804.671699 au
H(SMD/M06-2X/6-31G(d)) = -2804.330191 au
G(SMD/M06-2X/6-31G(d)) = -2804.446733 au
E(SMD/M06-2X/def2-TZVP//SMD/M06-2X /6-31G(d)) = -2805.880069 au

C	2.41742500	0.74291700	-1.44657900
C	3.72018700	0.37091900	-1.51143000
C	4.55742400	0.49930100	-0.35092300
C	4.28020100	-0.14021400	-2.73204300
C	5.87773100	0.13452200	-0.40524100
H	4.12699100	0.91137100	0.55612400
C	5.59800100	-0.51485100	-2.78398300
H	3.63691000	-0.22902600	-3.60113700
C	6.36354500	-0.37005600	-1.61898000
H	6.54768500	0.21969800	0.44245300
H	6.06195300	-0.91224700	-3.67924500
C	1.11576000	0.99876200	-1.57998800
O	0.25667900	0.02340400	-1.43867600
O	0.62633000	2.21385900	-1.81914700
C	1.58893300	3.27105200	-1.85419500
H	2.12416900	3.31830200	-0.90002100
H	2.29414100	3.11994900	-2.67830000
H	1.02460600	4.18800300	-2.01487500
B	-0.60775500	-0.11687300	-0.18544500
C	-1.53373800	1.21766800	0.04893100
C	-2.21853300	1.75603500	-1.03802200
C	-1.81486600	1.81773800	1.26963400
C	-3.08001900	2.83822500	-0.94670300
C	-2.67487400	2.90162200	1.40830300
C	-3.30987600	3.41786700	0.29212900
C	0.45623100	-0.42374500	1.02677600
C	0.75236500	-1.68216400	1.53864500
C	1.26360600	0.59895200	1.51814600
C	1.75539500	-1.91700400	2.47323000
C	2.28043900	0.41094800	2.44029900
C	2.53442400	-0.86429200	2.92063600
C	-1.67191200	-1.31938000	-0.47333300
C	-2.47621700	-1.74413700	0.58083900
C	-1.96692400	-1.89426800	-1.70282700
C	-3.48304200	-2.68549900	0.45961100
C	-2.97189100	-2.84446300	-1.87002800
C	-3.73329600	-3.24320600	-0.78665400
F	-1.25572500	1.37297300	2.40646000
F	-2.89594600	3.44535600	2.60918000
F	-4.13781800	4.45682400	0.40648400
F	-3.69887400	3.31757800	-2.03010900
F	-2.06587500	1.21521200	-2.25641900
F	-2.27193700	-1.22477500	1.80488400

F	-4.21182000	-3.05801100	1.51549300
F	-4.69815100	-4.15161200	-0.93572400
F	-3.20924300	-3.37063000	-3.07581200
F	-1.29692600	-1.56719600	-2.81643500
F	1.07990300	1.86243200	1.09001400
F	3.05157800	1.43448700	2.82584000
F	3.51713600	-1.07231700	3.79563000
F	1.98755400	-3.15260200	2.92299900
F	0.07626600	-2.77145200	1.14188500
F	7.62980900	-0.72841000	-1.67022300

1a•B(C_6F_5)$_3$ (R = H, R′ = OMe)
E(SMD/M06-2X/6-31G(d)) = -2705.463847 au
H(SMD/M06-2X/6-31G(d)) = -2705.115172 au
G(SMD/M06-2X/6-31G(d)) = -2705.231343 au
E(SMD/M06-2X/def2-TZVP//SMD/M06-2X /6-31G(d)) = -2706.624664 au

C	2.56213700	0.70703800	-1.49689600
C	3.82003000	0.24284100	-1.74936500
C	4.80538100	0.28399500	-0.71170700
C	4.16516500	-0.28535000	-3.03527500
C	6.07590600	-0.18831900	-0.95499700
H	4.52811700	0.70738200	0.24881400
C	5.43783200	-0.76134400	-3.25766800
H	3.40386800	-0.30723500	-3.80872700
C	6.38182700	-0.71070000	-2.22039300
H	6.83525800	-0.16130500	-0.18168800
H	5.71476600	-1.17169900	-4.22212900
C	1.25688100	0.97192000	-1.64399400
O	0.40160700	0.00617300	-1.45075400
O	0.77365900	2.18453700	-1.88781700
C	1.75343400	3.22161000	-2.00521300
H	2.32174300	3.29935300	-1.07226500
H	2.42534300	3.01875000	-2.84541700
H	1.20005500	4.14105300	-2.18726200
B	-0.42619600	-0.10943200	-0.16547800
C	-1.34635800	1.22734800	0.06783100
C	-2.06631300	1.74291100	-1.00725300
C	-1.59151600	1.84862200	1.28576500
C	-2.93135700	2.82166000	-0.90828600
C	-2.45361400	2.92971400	1.43173000
C	-3.12637600	3.42183000	0.32668300
C	0.68349300	-0.38588700	1.01049700
C	1.01386800	-1.63349700	1.52743500
C	1.50571600	0.65165400	1.44383300
C	2.06549200	-1.84507700	2.41274500
C	2.57271500	0.48623400	2.31216900
C	2.85795400	-0.77915400	2.80126200
C	-1.49210700	-1.32131000	-0.39788000
C	-2.25090200	-1.74174200	0.69109700
C	-1.83212000	-1.90832400	-1.60999800
C	-3.25482300	-2.69123000	0.61757500

C	-2.83648300	-2.86623700	-1.72945400
C	-3.55065000	-3.26129300	-0.61301300
F	-0.99224300	1.42866500	2.41129600
F	-2.64011000	3.49420800	2.62873800
F	-3.95665900	4.45802700	0.44797300
F	-3.58567800	3.27873900	-1.98032000
F	-1.94674700	1.18131000	-2.22007200
F	-2.00248800	-1.20953900	1.90116800
F	-3.93807500	-3.06020200	1.70466300
F	-4.51379000	-4.17780600	-0.71571200
F	-3.11809800	-3.40384400	-2.92051200
F	-1.20940900	-1.58589300	-2.75201900
F	1.28937900	1.90499600	1.00255400
F	3.35540800	1.51920900	2.64214800
F	3.88640000	-0.96622000	3.62736500
F	2.32980700	-3.07072300	2.87228400
F	0.32720700	-2.73378100	1.18217800
H	7.38427900	-1.08664000	-2.40433400

1a•B$(C_6F_5)_3$ (R = Cl, R′ = OMe)
E(SMD/M06-2X/6-31G(d)) = -3165.032635 au
H(SMD/M06-2X/6-31G(d)) = -3164.692364 au
G(SMD/M06-2X/6-31G(d)) = -3164.809592 au
E(SMD/M06-2X/def2-TZVP//SMD/M06-2X /6-31G(d)) = -3166.227046 au

C	2.16025500	1.14250600	-2.14849300
C	3.47314800	0.83394200	-1.82124700
C	3.93132600	0.69579600	-0.47322300
C	4.40593900	0.63987200	-2.88315000
C	5.24249500	0.38030000	-0.20408500
H	3.23442600	0.85753100	0.34378400
C	5.71976400	0.31684800	-2.62207400
H	4.05333200	0.74638300	-3.90381200
C	6.11855200	0.19347500	-1.28504600
H	5.60049100	0.27559700	0.81410100
H	6.43533600	0.16192400	-3.42132900
C	1.04571100	1.27796800	-1.30235500
O	0.34193800	0.23628800	-1.10624800
O	0.62583400	2.44795000	-0.89056300
C	1.52276400	3.55933000	-1.07324400
B	-0.74860900	-0.13089800	-0.07407000
C	-1.55783400	1.17610400	0.47465400
C	-2.14236600	2.03290900	-0.45461800
C	-1.83343100	1.47317300	1.80158600
C	-2.91695700	3.12791600	-0.11322100
C	-2.61256600	2.55838700	2.19117200
C	-3.15642300	3.39068500	1.22897700
C	0.12229800	-0.92800700	1.06149700
C	0.07651800	-2.27773200	1.38595800
C	1.12912400	-0.21922100	1.70927700
C	0.95403200	-2.87564700	2.28762200
C	2.04069700	-0.77115200	2.59037000

C	1.94550200	-2.12256100	2.89058600
C	-1.85044500	-1.04004100	-0.85657300
C	-2.88450000	-1.59464300	-0.10756400
C	-1.95525800	-1.22214000	-2.22942400
C	-3.93539800	-2.31253600	-0.65075600
C	-2.99598800	-1.93567000	-2.81803500
C	-3.98962100	-2.48425500	-2.02720700
F	-1.35379800	0.71275900	2.79772200
F	-2.84073500	2.79999400	3.48481100
F	-3.90260900	4.43581300	1.58469200
F	-3.43839300	3.92412100	-1.05029100
F	-1.94059500	1.81420400	-1.76496900
F	-2.87338900	-1.44182900	1.22832900
F	-4.89122600	-2.83149000	0.12412600
F	-4.99126700	-3.16815300	-2.57993500
F	-3.04596800	-2.08600200	-4.14483300
F	-1.05515100	-0.70820300	-3.07965100
F	1.27515700	1.09867300	1.44894200
F	3.00915100	-0.02565000	3.12946600
F	2.80377900	-2.68706700	3.73772800
F	0.85724100	-4.17922400	2.55677400
F	-0.81407200	-3.10761600	0.82392700
H	1.80569400	3.63940700	-2.12774600
H	0.96239200	4.44006200	-0.76649700
H	2.40648400	3.43184200	-0.44400000
Cl	7.76326900	-0.20110400	-0.94879400

1a•B(C_6F_5)$_3$ (R = CF_3, R′ = OMe)
E(SMD/M06-2X/6-31G(d)) = -3042.393013 au
H(SMD/M06-2X/6-31G(d)) = -3042.035556 au
G(SMD/M06-2X/6-31G(d)) = -3042.157924 au
E(SMD/M06-2X/def2-TZVP//SMD/M06-2X /6-31G(d)) = -3043.707829 au

C	-1.94186600	0.91951900	0.92934500
C	-3.26351200	0.54498700	0.95682200
C	-4.07364900	0.80863600	-0.18524900
C	-3.83264400	-0.08344800	2.10577600
C	-5.40967500	0.45686100	-0.17903000
H	-3.62176000	1.29895100	-1.04198700
C	-5.16498200	-0.43297500	2.10079400
H	-3.19975100	-0.27918400	2.96517300
C	-5.93359300	-0.15671300	0.96096800
H	-6.04200800	0.65185700	-1.03714100
H	-5.61927300	-0.91338600	2.96130800
C	-0.67917400	0.96926700	1.39840000
O	0.07425200	-0.07056000	1.19742400
O	-0.13088600	2.04735800	1.93156400
C	-1.00233100	3.17875100	2.06355600
B	1.14365300	-0.11767800	0.08757500
C	2.22770000	1.09585200	0.26934000
C	2.75797500	1.34165700	1.53347200
C	2.78473400	1.85040000	-0.75532300

C	3.73411700	2.29269800	1.78686000
C	3.76824000	2.81076800	-0.54745300
C	4.24477800	3.03544700	0.73275700
C	0.27385900	-0.05955900	-1.30000600
C	-0.08251700	-1.15979800	-2.07178100
C	-0.32776000	1.13537300	-1.68873200
C	-0.95909000	-1.09018900	-3.14923800
C	-1.22132400	1.24895100	-2.74155800
C	-1.53975000	0.12132100	-3.48242600
C	1.99095800	-1.49076900	0.30978500
C	2.91582700	-1.84838200	-0.66747400
C	1.99145000	-2.29671100	1.44081800
C	3.76874700	-2.93340400	-0.56858300
C	2.83335400	-3.39747700	1.58220100
C	3.72531300	-3.71884200	0.57520500
F	2.39005700	1.68798700	-2.02786000
F	4.25925000	3.51447100	-1.57169500
F	5.18727500	3.95280100	0.95019000
F	4.19417300	2.49190900	3.02557200
F	2.32607400	0.63069700	2.58651700
F	2.99439200	-1.11016900	-1.78894200
F	4.62689900	-3.22924500	-1.54832800
F	4.53602000	-4.76977300	0.70027800
F	2.78781400	-4.14387800	2.68996600
F	1.17352000	-2.06241300	2.47632600
F	-0.06448200	2.26677800	-1.00887800
F	-1.80624900	2.41759900	-3.02260200
F	-2.40182900	0.20222300	-4.49476000
F	-1.26106700	-2.18578600	-3.85004000
F	0.39913500	-2.38304500	-1.80357200
C	-7.39876600	-0.53070700	1.00959900
F	-7.55054000	-1.81104400	1.36502100
F	-8.00596900	-0.35569900	-0.16524400
F	-8.03937800	0.21515900	1.91797300
H	-1.83336500	2.94144200	2.73480500
H	-0.39712800	3.97686000	2.48890800
H	-1.37627900	3.47124900	1.07677600

1a•B(C_6F_5)$_3$ (R = CN, R′ = OMe)
E(SMD/M06-2X/6-31G(d)) = -2797.672702 au
H(SMD/M06-2X/6-31G(d)) = -2797.323941 au
G(SMD/M06-2X/6-31G(d)) = -2797.443607 au
E(SMD/M06-2X/def2-TZVP//SMD/M06-2X /6-31G(d)) = -2798.866284 au

C	-2.31911100	2.42583700	0.04661000
C	-3.63902900	1.98984200	-0.10446700
C	-4.56179700	2.88301200	-0.71644000
C	-4.08430900	0.70059700	0.31139300
C	-5.87696800	2.50940500	-0.91222600
H	-4.20617800	3.85941300	-1.02778800
C	-5.39661700	0.32346200	0.11873700
H	-3.38135600	0.01542900	0.77404900

C	-6.27777800	1.23237600	-0.49208100
H	-6.59147300	3.17709500	-1.37965700
H	-5.75357900	-0.65287600	0.42598400
C	-1.22550900	1.68283400	0.54657900
O	-0.57543100	0.98368500	-0.28717600
O	-0.77648200	1.84986400	1.76185600
C	-1.62474100	2.57954600	2.67154300
H	-1.89360300	3.54535300	2.23154000
H	-2.51667000	1.99120200	2.89630400
H	-1.03036600	2.72741900	3.57044800
B	0.69318700	0.10622300	-0.15177700
C	0.92052400	-0.45025600	1.36960900
C	-0.15686900	-0.97157200	2.07861400
C	2.15192500	-0.60293900	1.99503100
C	-0.05824500	-1.55666000	3.32942200
C	2.30290100	-1.19204400	3.24607800
C	1.19213900	-1.67015900	3.91961000
C	1.88266100	1.06285500	-0.72866000
C	2.37792200	1.02909800	-2.02725000
C	2.40456700	2.08277600	0.06348500
C	3.33742200	1.91488000	-2.50475600
C	3.36066700	2.98756400	-0.36972700
C	3.83215700	2.90226200	-1.67088900
C	0.37821300	-1.23158300	-1.03381600
C	1.42899800	-2.10046500	-1.31474900
C	-0.87621900	-1.68533100	-1.42342300
C	1.28025300	-3.31303800	-1.96481300
C	-1.07154200	-2.89887700	-2.07756600
C	0.00973000	-3.71687800	-2.35131000
F	3.28289500	-0.18035200	1.41266400
F	3.51221700	-1.30349500	3.79980100
F	1.32037700	-2.23408900	5.11957400
F	-1.14032600	-2.01885900	3.96045900
F	-1.39296400	-0.91242500	1.53870000
F	2.67419500	-1.75502500	-0.94482300
F	2.33284600	-4.09486100	-2.21548700
F	-0.16487300	-4.88135600	-2.97526900
F	-2.30065700	-3.28206900	-2.43484500
F	-1.99469200	-0.97849000	-1.18674300
F	1.98795400	2.22167200	1.33210400
F	3.82600000	3.93667600	0.44635200
F	4.74842600	3.76201500	-2.11238400
F	3.77591600	1.82579400	-3.76285300
F	1.94414200	0.11963300	-2.91334000
C	-7.64850300	0.83428300	-0.69322500
N	-8.74822300	0.51565500	-0.85321300

1a•B(C_6F_5)$_3$ (R = NO_2, R′ = OMe)
E(SMD/M06-2X/6-31G(d)) = -2909.88039 au
H(SMD/M06-2X/6-31G(d)) = -2909.526593 au
G(SMD/M06-2X/6-31G(d)) = -2909.648496 au

E(SMD/M06-2X/def2-TZVP//SMD/M06-2X /6-31G(d)) = -2911.128631 au

C	-2.15159600	2.32812200	0.27765200
C	-3.46461000	1.86630300	0.11630800
C	-4.41319400	2.77679000	-0.42304000
C	-3.87521600	0.54538900	0.45703000
C	-5.72653400	2.38960500	-0.62253700
H	-4.08384500	3.77837700	-0.67736700
C	-5.18528500	0.15246800	0.26296200
H	-3.15319700	-0.15329800	0.86591900
C	-6.06772100	1.08839800	-0.27197400
H	-6.46834200	3.06147500	-1.03528200
H	-5.52681800	-0.84460400	0.51075200
C	-1.01689700	1.60270000	0.70579800
O	-0.34020900	1.02064600	-0.19363000
O	-0.56330300	1.69067100	1.92739700
C	-1.45348000	2.26148200	2.90808000
H	-1.81855900	3.23235900	2.55691900
H	-2.28289900	1.57613700	3.09453500
H	-0.85544900	2.38909900	3.80761800
B	0.92757400	0.12832500	-0.15391500
C	1.20893400	-0.49887600	1.33031100
C	0.16237600	-1.09121800	2.02970000
C	2.45463600	-0.63532500	1.92962600
C	0.30082800	-1.72941300	3.24998500
C	2.64627600	-1.27660000	3.14923400
C	1.56446200	-1.82637300	3.81485900
C	2.09588500	1.11239600	-0.72521500
C	2.60742400	1.10665800	-2.01727700
C	2.58659900	2.13079000	0.08824400
C	3.55448800	2.02006100	-2.46844000
C	3.52847600	3.06182800	-0.31751300
C	4.01814700	3.00454100	-1.61397000
C	0.57952000	-1.16306900	-1.08967700
C	1.61523100	-2.03550900	-1.41274800
C	-0.68345200	-1.57939600	-1.49130000
C	1.44396200	-3.21685400	-2.11252500
C	-0.90192000	-2.76074700	-2.19497100
C	0.16490400	-3.58328000	-2.50873700
F	3.56004100	-0.14418100	1.35234500
F	3.86742800	-1.37056200	3.67945900
F	1.73273600	-2.44218300	4.98382300
F	-0.75412200	-2.25769100	3.87487300
F	-1.08446400	-1.04430600	1.51285800
F	2.86720100	-1.72335500	-1.03594600
F	2.48210600	-4.00432300	-2.40229100
F	-0.03154100	-4.71679600	-3.18107800
F	-2.13862200	-3.10896800	-2.56140500
F	-1.78858800	-0.86456500	-1.21708700
F	2.14770300	2.23595800	1.35286500
F	3.96397500	4.00802100	0.51801400

F 4.92202200 3.88913600 -2.03118200
F 4.01042100 1.95931900 -3.72199300
F 2.20447300 0.20293800 -2.92334500
N -7.47575700 0.66519000 -0.48126500
O -7.76528100 -0.47764400 -0.18898800
O -8.24606000 1.49023500 -0.93016000

1 (R = H, R′ = Me)

E(SMD/M06-2X/6-31G(d)) = -532.2397061 au
H(SMD/M06-2X/6-31G(d)) = -532.074358 au
G(SMD/M06-2X/6-31G(d)) = -532.120356 au
E(SMD/M06-2X/def2-TZVP//SMD/M06-2X /6-31G(d)) = -532.4463632 au

C 0.90079600 0.17831600 0.00002000
N 1.38562200 1.40354500 0.00016600
N 1.79736600 2.45234100 0.00046700
C -0.57057000 0.05028900 -0.00004200
C -1.18809800 -1.20958900 0.00009000
C -1.38191100 1.19684400 -0.00023900
C -2.57726900 -1.30703000 0.00003300
H -0.57896100 -2.10299800 0.00023500
C -2.76711100 1.08694000 -0.00028800
H -0.93550400 2.18786500 -0.00036600
C -3.37584500 -0.16672800 -0.00015000
H -3.03540000 -2.29183400 0.00014000
H -3.37135600 1.98921600 -0.00043800
H -4.45800400 -0.25183600 -0.00018800
C 1.92144600 -0.88040600 -0.00001700
O 1.61119000 -2.06100200 -0.00010100
C 3.37265400 -0.44805500 0.00005700
H 3.60469900 0.15309400 -0.88597100
H 3.60462900 0.15303400 0.88614300
H 3.99491700 -1.34320900 0.00005100

1 (R = H, R′ = H)

E(SMD/M06-2X/6-31G(d)) = -492.9353776 au
H(SMD/M06-2X/6-31G(d)) = -492.798718 au
G(SMD/M06-2X/6-31G(d)) = -492.844035 au
E(SMD/M06-2X/def2-TZVP//SMD/M06-2X /6-31G(d)) = -493.1284201 au

C -1.26187600 -0.03392400 -0.00007800
N -1.88151600 -1.20126700 -0.00009700
N -2.41460700 -2.19166100 -0.00000200
C 0.21169200 -0.03737900 0.00000000
C 0.91984000 1.17309500 0.00010300
C 0.92768000 -1.24403500 -0.00003100
C 2.31200000 1.16473100 0.00016700
H 0.37752300 2.10963700 0.00012900
C 2.31744900 -1.23933900 0.00003700
H 0.40288000 -2.19591400 -0.00011100
C 3.01899300 -0.03493600 0.00013500
H 2.84553400 2.11065100 0.00024500
H 2.85267100 -2.18406400 0.00001000
H 4.10452200 -0.03316800 0.00018600

C	-2.18339800	1.09139900	-0.00012900
O	-1.85435500	2.26359700	-0.00010300
H	-3.24972400	0.79690800	-0.00016600

1 (R = H, R′ = Ph)
E(SMD/M06-2X/6-31G(d)) = -723.9061158 au
H(SMD/M06-2X/6-31G(d)) = -723.682848 au
G(SMD/M06-2X/6-31G(d)) = -723.738866 au
E(SMD/M06-2X/def2-TZVP//SMD/M06-2X /6-31G(d)) = -724.1798414 au

C	-0.51631300	0.26596400	0.08372700
N	-0.10206100	1.50333100	0.26735400
N	0.21656400	2.57048300	0.43739100
C	-1.97880000	0.06440100	0.01203700
C	-2.50734700	-1.12677100	-0.50608300
C	-2.86175000	1.06800400	0.43627500
C	-3.88543900	-1.30143900	-0.58876800
H	-1.84061000	-1.91263000	-0.83693300
C	-4.23727400	0.88636900	0.34385500
H	-2.47839300	1.99820300	0.84776000
C	-4.75789800	-0.30063400	-0.16691200
H	-4.27729300	-2.23039800	-0.99231400
H	-4.90224400	1.67659300	0.67911900
H	-5.83176200	-0.44386700	-0.23530900
C	0.50858200	-0.78571300	0.11673100
O	0.19437700	-1.95850400	0.25969700
C	1.95307000	-0.40058000	-0.00351600
C	2.87758300	-1.10523200	0.77339900
C	2.39895400	0.57277500	-0.90264500
C	4.23376900	-0.81501000	0.67791500
H	2.51984900	-1.87421200	1.45134600
C	3.75963300	0.84954400	-1.00792400
H	1.69351900	1.09419900	-1.54467800
C	4.67550800	0.16390400	-0.21240600
H	4.94755600	-1.35406600	1.29310900
H	4.10391000	1.59709300	-1.71567500
H	5.73527100	0.38691900	-0.29133100

1 (R = H, R′ = OH)
E(SMD/M06-2X/6-31G(d)) = -568.1599856 au
H(SMD/M06-2X/6-31G(d)) = -568.018327 au
G(SMD/M06-2X/6-31G(d)) = -568.063266 au
E(SMD/M06-2X/def2-TZVP//SMD/M06-2X /6-31G(d)) = -568.3909031 au

C	0.92062800	0.19961100	0.00007900
N	1.42229300	1.41816300	0.00024900
N	1.83360400	2.46514700	0.00016200
C	-0.54674900	0.06059300	0.00004800
C	-1.15311600	-1.20412500	0.00021500
C	-1.36439000	1.20177400	-0.00012500
C	-2.54109900	-1.31241100	0.00021800
H	-0.53907600	-2.09495900	0.00034700
C	-2.74883200	1.08044800	-0.00011200
H	-0.92426200	2.19561000	-0.00028400

C	-3.34790600	-0.17775100	0.00005900
H	-2.99150300	-2.30069300	0.00035300
H	-3.36007900	1.97791800	-0.00024400
H	-4.42931000	-0.27119700	0.00006600
C	1.91982000	-0.86522800	-0.00013500
O	1.67918700	-2.05343200	-0.00041300
O	3.17605600	-0.37700400	-0.00011800
H	3.78087500	-1.14382700	-0.00034200

1 (R = H, R′ = F)
E(SMD/M06-2X/6-31G(d)) = -592.1641238 au
H(SMD/M06-2X/6-31G(d)) = -592.035254 au
G(SMD/M06-2X/6-31G(d)) = -592.079864 au
E(SMD/M06-2X/def2-TZVP//SMD/M06-2X /6-31G(d)) = -592.40431 au

C	0.92605800	0.17541100	0.00002100
N	1.45886900	1.38630600	0.00011300
N	1.88300700	2.42502200	-0.00000100
C	-0.54438500	0.05955200	0.00004800
C	-1.16268200	-1.19832300	0.00016200
C	-1.34445500	1.21161100	-0.00002700
C	-2.55171900	-1.29006600	0.00020800
H	-0.56204800	-2.09875800	0.00021700
C	-2.73036600	1.10637500	0.00002500
H	-0.89150400	2.19952300	-0.00013600
C	-3.34343700	-0.14489300	0.00014300
H	-3.01422300	-2.27252600	0.00029900
H	-3.33131600	2.01058400	-0.00003300
H	-4.42582700	-0.22528500	0.00018200
C	1.88955700	-0.89913200	-0.00015300
O	1.69762200	-2.07807500	-0.00026300
F	3.15770800	-0.42127100	-0.00019800

1′ (R = H, R′ = Me)
E(SMD/M06-2X/6-31G(d)) = -532.1808618 au
H(SMD/M06-2X/6-31G(d)) = -532.018101 au
G(SMD/M06-2X/6-31G(d)) = -532.068893 au
E(SMD/M06-2X/def2-TZVP//SMD/M06-2X /6-31G(d)) = -532.3905711 au

C	-0.87896400	0.08766100	-0.64680300
N	-1.45190900	1.66778300	0.39935200
N	-1.59805100	2.75626800	0.48492500
C	0.52521000	-0.02511200	-0.28272800
C	1.43334900	0.91445200	-0.80427100
C	1.01319200	-1.05788100	0.54172000
C	2.78609600	0.83843100	-0.49918600
H	1.05223500	1.70336200	-1.44705500
C	2.37042100	-1.15379100	0.81719400
H	0.32105100	-1.79585700	0.93521000
C	3.25405500	-0.20084600	0.30604200
H	3.47808300	1.57353400	-0.89762000
H	2.74281200	-1.96060100	1.44071800
H	4.31363700	-0.27175900	0.53379500
C	-1.85880800	-0.90662300	-0.29252900

O	-1.64415400	-2.03598600	-0.74151200
C	-3.14638100	-0.57089900	0.42224900
H	-2.95434800	-0.37452600	1.48237400
H	-3.61952000	0.31774900	-0.00608600
H	-3.82002700	-1.42471800	0.33069500

1′ (R = H, R′ = H)
E(SMD/M06-2X/6-31G(d)) = -492.8765676 au
H(SMD/M06-2X/6-31G(d)) = -492.743532 au
G(SMD/M06-2X/6-31G(d)) = -492.791082 au
E(SMD/M06-2X/def2-TZVP//SMD/M06-2X /6-31G(d)) = -493.0726905 au

C	-1.20430200	0.11408200	0.55026900
N	-1.98823300	-1.38430900	-0.50685800
N	-2.27145200	-2.44235900	-0.61862000
C	0.20847100	0.05469900	0.25090900
C	0.99600900	-0.93882100	0.86392200
C	0.82806900	0.99143200	-0.60191100
C	2.36043000	-1.00795800	0.61897200
H	0.51189300	-1.65036900	1.52690600
C	2.19807800	0.94274300	-0.81620500
H	0.22517600	1.76665600	-1.06500600
C	2.95964500	-0.06233200	-0.21533200
H	2.96133400	-1.78234200	1.08467100
H	2.67527800	1.67442100	-1.46036600
H	4.02963400	-0.10615300	-0.39648400
C	-2.09143100	1.15452100	0.13589100
O	-1.88564400	2.31733800	0.47955300
H	-3.03018400	0.85557400	-0.36688900

1′ (R = H, R′ = Ph)
E(SMD/M06-2X/6-31G(d)) = -723.8481604 au
H(SMD/M06-2X/6-31G(d)) = -723.628412 au
G(SMD/M06-2X/6-31G(d)) = -723.686858 au
E(SMD/M06-2X/def2-TZVP//SMD/M06-2X /6-31G(d)) = -724.124156 au

C	-0.56177300	0.29279500	0.75936700
N	-0.01053300	1.65033000	-0.58824300
N	-0.01345200	2.66137000	-1.02376300
C	-1.92091800	0.03737400	0.30756500
C	-2.94066300	0.89734100	0.75234300
C	-2.25557100	-1.04073200	-0.53634400
C	-4.25959000	0.69204000	0.36610100
H	-2.67395600	1.72400700	1.40460400
C	-3.57486600	-1.25515800	-0.90781600
H	-1.47361100	-1.71276200	-0.87552800
C	-4.57403400	-0.38625700	-0.46076400
H	-5.04124300	1.36113000	0.71121200
H	-3.83066600	-2.09107000	-1.55127900
H	-5.60488300	-0.55379500	-0.75909300
C	0.47862300	-0.70085500	0.66356400
O	0.20002700	-1.83029500	1.08179700
C	1.89163600	-0.37031600	0.29142200
C	2.64111100	-1.36903400	-0.33576600

C	2.49311300	0.85186800	0.60729600
C	3.96926100	-1.13664600	-0.67831600
H	2.17024800	-2.32314900	-0.55357400
C	3.82670900	1.07703900	0.27703400
H	1.92748200	1.61340100	1.13676400
C	4.56298700	0.08777700	-0.37338200
H	4.54305700	-1.91129400	-1.17783000
H	4.29349200	2.02279700	0.53479100
H	5.60160700	0.26777100	-0.63424600

1′ (R = H, R′ = OH)
E(SMD/M06-2X/6-31G(d)) = -568.1015258 au
H(SMD/M06-2X/6-31G(d)) = -567.962251 au
G(SMD/M06-2X/6-31G(d)) = -568.011706 au
E(SMD/M06-2X/def2-TZVP//SMD/M06-2X /6-31G(d)) = -568.3351721 au

C	0.88362000	0.12251600	0.66786800
N	1.51809500	1.64681300	-0.43101700
N	1.79853500	2.70596800	-0.53813200
C	-0.51408200	0.00126700	0.28775800
C	-1.43188100	0.90321500	0.85783700
C	-0.99168000	-0.99160600	-0.59116900
C	-2.78431000	0.82669200	0.55210600
H	-1.05722200	1.66007800	1.54113000
C	-2.34702100	-1.08628800	-0.87329200
H	-0.29302900	-1.69919000	-1.02818700
C	-3.24011800	-0.17242800	-0.30881400
H	-3.48485200	1.53026200	0.99027200
H	-2.71164300	-1.86116800	-1.54014100
H	-4.29907700	-0.24310000	-0.53961300
C	1.82555800	-0.91187200	0.28556700
O	1.67670800	-2.04027200	0.72806700
O	2.91470900	-0.54438000	-0.41071700
H	3.49754900	-1.32810300	-0.44538800

1′ (R = H, R′ = F)
E(SMD/M06-2X/6-31G(d)) = -592.1071253 au
H(SMD/M06-2X/6-31G(d)) = -591.980704 au
G(SMD/M06-2X/6-31G(d)) = -592.029956 au
E(SMD/M06-2X/def2-TZVP//SMD/M06-2X /6-31G(d)) = -592.3492209 au

C	0.87856800	0.08530200	0.64785600
N	1.55019700	1.60105000	-0.45089700
N	1.79441600	2.66677800	-0.57581400
C	-0.51585000	-0.00653300	0.28177100
C	-1.42278300	0.87006700	0.90916700
C	-1.00434300	-0.95319200	-0.64324300
C	-2.77818300	0.80922000	0.61760600
H	-1.03672900	1.59110400	1.62380200
C	-2.36200500	-1.02908200	-0.91423800
H	-0.31084500	-1.63648100	-1.12508200
C	-3.24455100	-0.14341200	-0.28987100
H	-3.47309200	1.48935000	1.09911100
H	-2.73784300	-1.76616400	-1.61640900

H	-4.30624800	-0.19949600	-0.51180500
C	1.81416500	-0.94582600	0.28764400
O	1.71078600	-2.09077200	0.63883100
F	2.95289600	-0.52735500	-0.30815900

1a (R = H, R′ = Me)
E(SMD/M06-2X/6-31G(d)) = -422.7074864 au
H(SMD/M06-2X/6-31G(d)) = -422.554914 au
G(SMD/M06-2X/6-31G(d)) = -422.600805 au
E(SMD/M06-2X/def2-TZVP//SMD/M06-2X /6-31G(d)) = -422.8741275 au

C	1.05566800	-0.94720300	-0.19595800
C	-0.25959700	-0.42097300	-0.08817500
C	-1.34724100	-1.32882000	-0.06471500
C	-0.53288900	0.97109100	-0.01572000
C	-2.65115400	-0.86981300	0.02965900
H	-1.12698800	-2.39033700	-0.12356800
C	-1.83542800	1.42796900	0.07266100
H	0.29623400	1.67352300	-0.04186800
C	-2.88824100	0.50547200	0.09725700
H	-3.48261500	-1.56640800	0.04838700
H	-2.04506600	2.49124300	0.12443100
H	-3.91003900	0.86728500	0.16899800
C	2.20506600	-0.10359800	-0.19335100
O	2.71239100	0.38290600	-1.20439200
C	2.84211800	0.03529700	1.18011900
H	2.23460400	-0.40866400	1.97435400
H	3.82362900	-0.44848400	1.17239800
H	2.98130200	1.10206100	1.38133900

1a (R = H, R′ = H)
E(SMD/M06-2X/6-31G(d)) = -383.4010252 au
H(SMD/M06-2X/6-31G(d)) = -383.278613 au
G(SMD/M06-2X/6-31G(d)) = -383.32025 au
E(SMD/M06-2X/def2-TZVP//SMD/M06-2X /6-31G(d)) = -383.5535515 au

C	1.41409900	-0.88918300	0.10696200
C	0.08968400	-0.39393400	0.07274100
C	-0.97496200	-1.32426700	-0.04083700
C	-0.21723300	0.99373200	0.11464100
C	-2.28823700	-0.89041900	-0.10892100
H	-0.72861700	-2.38103000	-0.07513600
C	-1.52908300	1.42399400	0.04214400
H	0.59650000	1.70936400	0.19573300
C	-2.55798700	0.48028300	-0.06684800
H	-3.10205700	-1.60230700	-0.19603700
H	-1.76515800	2.48248900	0.07113700
H	-3.58773300	0.82207600	-0.12059700
C	2.56628400	-0.09904300	0.31335300
O	3.34836100	0.40822400	-0.48608200
H	2.78479000	-0.10336800	1.41414700

1a (R = H, R′ = Ph)
E(SMD/M06-2X/6-31G(d)) = -614.3788097 au
H(SMD/M06-2X/6-31G(d)) = -614.169033 au

G(SMD/M06-2X/6-31G(d)) = -614.222661 au
E(SMD/M06-2X/def2-TZVP//SMD/M06-2X /6-31G(d)) = -614.6110687 au

C	0.60499200	0.62197400	1.16796000
C	1.75740700	0.20814000	0.45340000
C	2.86117400	-0.28661000	1.19328700
C	1.85183600	0.25081000	-0.96457100
C	4.00707800	-0.72422500	0.54987900
H	2.77810300	-0.31221100	2.27547000
C	2.99901500	-0.18115900	-1.60423500
H	1.01397000	0.63691800	-1.53873400
C	4.07011800	-0.66839700	-0.84488100
H	4.84955000	-1.10414300	1.11789100
H	3.07330900	-0.14747400	-2.68608300
H	4.96846900	-1.00908500	-1.35194600
C	-0.52519200	1.19238400	0.49627300
O	-0.51294800	2.40140500	0.23987900
C	-1.72342300	0.33509300	0.24165800
C	-2.82097000	0.89952300	-0.41103800
C	-1.74246500	-1.01407400	0.60624000
C	-3.93443000	0.11753900	-0.69898200
H	-2.78156000	1.94901200	-0.68684400
C	-2.85657500	-1.79584100	0.31583700
H	-0.89228400	-1.44927200	1.12715600
C	-3.95208600	-1.22972300	-0.33594100
H	-4.78908100	0.55509500	-1.20598000
H	-2.87346800	-2.84316400	0.60093800
H	-4.82228800	-1.83951600	-0.56021800

1a (R = H, R′ = OH)
E(SMD/M06-2X/6-31G(d)) = -458.6269486 au
H(SMD/M06-2X/6-31G(d)) = -458.497667 au
G(SMD/M06-2X/6-31G(d)) = -458.541357 au
E(SMD/M06-2X/def2-TZVP//SMD/M06-2X /6-31G(d)) = -458.817644 au

C	1.05542000	-1.02388200	-0.04066100
C	-0.24074600	-0.44614900	-0.02823400
C	-1.35123400	-1.32844000	-0.00875400
C	-0.48600400	0.95469600	-0.01660100
C	-2.64641800	-0.83919300	0.02810600
H	-1.15169700	-2.39553800	-0.02030000
C	-1.77935500	1.44132700	0.01255400
H	0.35605900	1.64201700	-0.03807300
C	-2.85342000	0.54254600	0.03701100
H	-3.49357700	-1.51648200	0.04673700
H	-1.96721000	2.50979800	0.01885100
H	-3.86829800	0.92916500	0.06266300
C	2.19138100	-0.12821500	-0.09506900
O	2.67062200	0.25351600	-1.14892500
O	2.73398100	0.13913900	1.10578300
H	3.55015300	0.65367000	0.94515400

1a (R = H, R′ = F)
E(SMD/M06-2X/6-31G(d)) = -482.6329535 au

H(SMD/M06-2X/6-31G(d)) = -482.516434 au
G(SMD/M06-2X/6-31G(d)) = -482.559919 au
E(SMD/M06-2X/def2-TZVP//SMD/M06-2X /6-31G(d)) = -482.8325263 au

C	-1.06684700	-0.95358500	0.11910800
C	0.23474900	-0.41712300	0.06006000
C	1.32455100	-1.32947600	0.07477400
C	0.51267900	0.97825700	-0.01382800
C	2.62888000	-0.87268800	0.01420800
H	1.09922900	-2.38978600	0.13310500
C	1.81610500	1.43028400	-0.06912400
H	-0.31445200	1.68370900	-0.01965000
C	2.86702200	0.50325200	-0.05643200
H	3.46075800	-1.56856300	0.02351100
H	2.03099900	2.49207900	-0.12339300
H	3.89081800	0.86358400	-0.10155100
C	-2.20730700	-0.08476300	0.13029800
O	-2.77024700	0.39907200	1.07176000
F	-2.74048500	0.02238400	-1.11561000

1′•B(C_6F_5)$_3$ (R = H, R′ = Me)
E(SMD/M06-2X/6-31G(d)) = -2739.758245 au
H(SMD/M06-2X/6-31G(d)) = -2739.406337 au
G(SMD/M06-2X/6-31G(d)) = -2739.524287 au
E(SMD/M06-2X/def2-TZVP//SMD/M06-2X /6-31G(d)) = -2740.929869 au

C	-2.64329800	0.38328900	1.61173600
N	-2.33252700	-1.56158600	1.37847900
N	-2.43571500	-2.64963100	1.27111600
C	-4.04531000	0.54170200	1.67018900
C	-4.75440000	0.85029400	0.48843500
C	-4.72544000	0.41750900	2.90114900
C	-6.12396700	1.04931200	0.54770000
H	-4.21364200	0.93989500	-0.44842900
C	-6.09564800	0.61329100	2.94526300
H	-4.16167500	0.17357800	3.79615200
C	-6.78757400	0.92820200	1.77171000
H	-6.67857300	1.29657700	-0.35100100
H	-6.62994000	0.52534200	3.88497600
C	-1.39718200	0.83906500	1.54355500
O	-0.31670700	0.09901400	1.44697700
B	0.53910900	-0.04835000	0.19865800
C	1.15659900	1.39334300	-0.29428600
C	1.78512600	2.20631500	0.64659700
C	1.23398000	1.85507200	-1.60298700
C	2.40184300	3.40995300	0.34213900
C	1.84766800	3.05204400	-1.95580300
C	2.43353400	3.83657500	-0.97706900
C	-0.44261200	-0.77637700	-0.90238400
C	-0.44454600	-2.13066700	-1.21850500
C	-1.50480100	-0.06247500	-1.45077900
C	-1.41761700	-2.73568400	-2.00767100
C	-2.50127500	-0.62102900	-2.23565800

C	-2.46040300	-1.97821000	-2.51442100
C	1.84702800	-0.93020700	0.61881900
C	2.71676100	-1.33052700	-0.39214400
C	2.27396200	-1.22397300	1.90755000
C	3.90878000	-1.99782200	-0.17291700
C	3.46697400	-1.89221100	2.17378300
C	4.28830600	-2.28184100	1.13169200
F	0.70868500	1.15350400	-2.61898700
F	1.88012900	3.44857900	-3.23076000
F	3.02327300	4.98749300	-1.29810000
F	2.97201100	4.15101600	1.29570200
F	1.80650200	1.83526100	1.93888100
F	2.39011500	-1.07189300	-1.67136900
F	4.69254300	-2.36526000	-1.19035200
F	5.43223600	-2.92217200	1.37525500
F	3.82704700	-2.15399400	3.43425300
F	1.55937300	-0.87700500	2.98694300
F	-1.61317500	1.25998700	-1.21616300
F	-3.50144300	0.13084300	-2.70667100
F	-3.40632500	-2.54281500	-3.26184000
F	-1.36333500	-4.04386700	-2.26597800
F	0.49242300	-2.96124500	-0.73608300
H	-7.86167000	1.08273000	1.81237900
C	-1.28562000	2.34211900	1.68132900
H	-0.52712300	2.55061700	2.44004700
H	-0.96569300	2.75920700	0.72296400
H	-2.23161200	2.80195800	1.97192300

1′• B(C_6F_5)$_3$ (R = H, R′ = H)
E(SMD/M06-2X/6-31G(d)) = -2700.454818 au
H(SMD/M06-2X/6-31G(d)) = -2700.132438 au
G(SMD/M06-2X/6-31G(d)) = -2700.245914 au
E(SMD/M06-2X/def2-TZVP//SMD/M06-2X /6-31G(d)) = -2701.613288 au

C	-2.65455400	0.51106500	1.55717100
N	-2.44504200	-1.42480000	1.61320700
N	-2.59228700	-2.50891800	1.51656800
C	-4.03729800	0.78321500	1.62977000
C	-4.80266400	0.76099700	0.44293500
C	-4.64066500	1.08484900	2.86927500
C	-6.15424200	1.05543300	0.50086200
H	-4.31590500	0.52062600	-0.49791800
C	-5.99303600	1.38139300	2.91147100
H	-4.03369900	1.08765800	3.76890000
C	-6.74214900	1.36377000	1.73142900
H	-6.75379000	1.04889600	-0.40282100
H	-6.46962900	1.62418400	3.85496300
C	-1.38788600	0.86497600	1.40846500
O	-0.33694500	0.08919400	1.44910600
B	0.55039300	-0.05206200	0.21877500
C	0.90885600	1.40847400	-0.43541000
C	1.29948700	2.45282200	0.39740000

C	0.99305800	1.68345300	-1.79591900
C	1.70826900	3.69660900	-0.06206900
C	1.40310900	2.91093100	-2.30083700
C	1.76169300	3.92557900	-1.42797900
C	-0.34120200	-1.03469300	-0.75402400
C	-0.21468200	-2.41840000	-0.80976100
C	-1.47338400	-0.53714900	-1.39617200
C	-1.13207600	-3.25031800	-1.44343600
C	-2.42004600	-1.32693700	-2.03079100
C	-2.25002800	-2.70249200	-2.05181200
C	1.97452600	-0.67297700	0.70495600
C	2.87125400	-1.11026800	-0.26546000
C	2.45776700	-0.70083200	2.00694400
C	4.14641100	-1.57237100	0.00958200
C	3.73423800	-1.15620200	2.32791100
C	4.58237700	-1.59494400	1.32725400
F	0.67096100	0.75438900	-2.70779600
F	1.45632300	3.12192100	-3.61829300
F	2.15369300	5.10934500	-1.89695600
F	2.05709200	4.66339900	0.79027300
F	1.28971100	2.27867900	1.73108600
F	2.49151200	-1.09589100	-1.55540500
F	4.95653500	-1.98831800	-0.96749000
F	5.80690400	-2.03133500	1.62292200
F	4.14765700	-1.16700300	3.59881200
F	1.71741100	-0.28545000	3.04393700
F	-1.71433100	0.78756600	-1.40857300
F	-3.49680000	-0.77552400	-2.59975600
F	-3.14396800	-3.48458700	-2.65318300
F	-0.95181200	-4.57237800	-1.45845600
F	0.81190300	-3.04530500	-0.21457700
H	-7.80260300	1.59416400	1.77126200
H	-1.27740600	1.94578600	1.24101800

1′•B(C_6F_5)$_3$ (R = H, R′ = Ph)
E(SMD/M06-2X/6-31G(d)) = -2931.426627 au
H(SMD/M06-2X/6-31G(d)) = -2931.017982 au
G(SMD/M06-2X/6-31G(d)) = -2931.142321 au
E(SMD/M06-2X/def2-TZVP//SMD/M06-2X /6-31G(d)) = -2932.662721 au

C	2.50598600	-0.05250500	-1.42262100
N	1.95105700	-1.90976100	-1.65421200
N	1.92327200	-2.99369700	-1.83057100
C	3.92050000	-0.06783300	-1.46359400
C	4.64418900	-0.27687100	-0.26982600
C	4.59513500	0.13258800	-2.68576200
C	6.02888300	-0.26736100	-0.30314100
H	4.10352300	-0.44018300	0.65763600
C	5.98046500	0.14803600	-2.70327200
H	4.01817100	0.28314000	-3.59263900
C	6.68993600	-0.05325700	-1.51605400
H	6.59726600	-0.42277500	0.60742800

H	6.51228400	0.31304800	-3.63403000
C	1.33631600	0.54451500	-1.22292600
O	0.15183800	-0.01998800	-1.31197500
B	-0.74170300	-0.31212800	-0.10114700
C	-0.96161800	1.04050200	0.80388400
C	-1.41572200	2.18834200	0.15665900
C	-0.77992800	1.18483000	2.17418100
C	-1.56153600	3.42058000	0.77400200
C	-0.93308400	2.39589700	2.84040700
C	-1.31453600	3.52445000	2.13462600
C	0.02485400	-1.54904200	0.67506000
C	-0.31362100	-2.89496900	0.56678900
C	1.23146800	-1.32460800	1.33565800
C	0.46952000	-3.93142800	1.06563400
C	2.04661900	-2.32209800	1.84804700
C	1.66422300	-3.64647000	1.70567400
C	-2.21628500	-0.70147500	-0.67848000
C	-3.16622500	-1.18107100	0.21851600
C	-2.68567800	-0.48637500	-1.96783100
C	-4.47842600	-1.46538200	-0.11727500
C	-3.99754000	-0.75674600	-2.34886000
C	-4.89846200	-1.24959800	-1.42223100
F	-0.43455600	0.14204000	2.94523200
F	-0.72219700	2.47768700	4.15735200
F	-1.45688500	4.69362000	2.75808500
F	-1.94978500	4.49424300	0.08099600
F	-1.71950400	2.13855100	-1.15042200
F	-2.80191300	-1.40519500	1.49388500
F	-5.33712000	-1.93733300	0.79092600
F	-6.15709500	-1.51152600	-1.77650100
F	-4.39495300	-0.53507700	-3.60594300
F	-1.89877600	0.00940600	-2.93318900
F	1.68477700	-0.06616200	1.50169800
F	3.19593800	-2.01702800	2.45814000
F	2.42808100	-4.62654200	2.18194000
F	0.08369000	-5.20014300	0.92048400
F	-1.42774900	-3.28821500	-0.06848900
H	7.77565500	-0.04417500	-1.53629700
C	1.45906300	2.02743600	-1.02235800
C	0.81297800	2.85423600	-1.94656400
C	2.12984700	2.57201900	0.07383900
C	0.81122000	4.23032300	-1.75014700
H	0.29032300	2.41146500	-2.78866200
C	2.12062800	3.95174000	0.26436800
H	2.63437200	1.92179900	0.78079000
C	1.45794100	4.77829400	-0.64126400
H	0.29946400	4.87536300	-2.45747800
H	2.62612700	4.37924100	1.12456600
H	1.44610300	5.85268900	-0.48488000

1′•$B(C_6F_5)_3$ (R = H, R′ = OH)

E(SMD/M06-2X/6-31G(d)) = -2775.653778 au
H(SMD/M06-2X/6-31G(d)) = -2775.325804 au
G(SMD/M06-2X/6-31G(d)) = -2775.441403 au
E(SMD/M06-2X/def2-TZVP//SMD/M06-2X /6-31G(d)) = -2776.848871 au

C	-2.59618600	0.38259200	1.87573700
N	-2.33381000	-1.60474000	1.47499700
N	-2.45065400	-2.69136900	1.36327000
C	-3.98034700	0.59191900	1.75545200
C	-4.55853300	0.91991600	0.50234400
C	-4.80151800	0.48332500	2.90443100
C	-5.91889600	1.15227600	0.41443100
H	-3.91927300	0.99537600	-0.37097000
C	-6.16192500	0.71467000	2.80317400
H	-4.34206300	0.22294100	3.85265200
C	-6.71336300	1.04672700	1.56160000
H	-6.36939200	1.41085600	-0.53770100
H	-6.79802400	0.63995600	3.67829500
C	-1.34874700	0.82065000	1.55517000
O	-0.30722100	0.07280800	1.43799800
B	0.56559000	-0.06314200	0.18004400
C	1.05217400	1.39707100	-0.37439300
C	1.62100200	2.29650500	0.52435600
C	1.06967600	1.80580500	-1.70123000
C	2.13411800	3.53029300	0.15723600
C	1.57929600	3.03158100	-2.11516700
C	2.11323500	3.90067900	-1.17956200
C	-0.39164900	-0.90815400	-0.85220600
C	-0.30795100	-2.26823100	-1.12481300
C	-1.52483200	-0.28958700	-1.37296400
C	-1.27263100	-2.96557000	-1.84656500
C	-2.51479500	-0.93979200	-2.09084800
C	-2.38804200	-2.30051500	-2.32753000
C	1.92635200	-0.83500700	0.63141300
C	2.81218400	-1.22809600	-0.36788600
C	2.37668000	-1.03976700	1.92922800
C	4.04379800	-1.81103700	-0.12758600
C	3.60914200	-1.62171000	2.21627300
C	4.44653500	-2.00924200	1.18597000
F	0.58029900	1.02093500	-2.67422800
F	1.56042600	3.37472000	-3.40634800
F	2.60427200	5.08060300	-1.55812000
F	2.65538300	4.35699100	1.06820500
F	1.69258200	1.97806700	1.82562700
F	2.46013600	-1.05039700	-1.65374500
F	4.84305300	-2.17761100	-1.13287800
F	5.62847600	-2.56662200	1.45000700
F	3.99093600	-1.80359100	3.48402400
F	1.64592700	-0.68548000	2.99485300
F	-1.71333300	1.03073700	-1.16366000
F	-3.58264500	-0.27554900	-2.54207500

F	-3.32240700	-2.95533300	-3.01272200
F	-1.13655800	-4.27390600	-2.06830300
F	0.71239000	-3.01051100	-0.67086900
H	-7.78166600	1.22693800	1.48564400
O	-1.13641500	2.14224100	1.55458100
H	-1.97628000	2.64072100	1.50902200

1′•B(C_6F_5)$_3$ (R = H, R′ = F)
E(SMD/M06-2X/6-31G(d)) = -2799.657585 au
H(SMD/M06-2X/6-31G(d)) = -2799.342125 au
G(SMD/M06-2X/6-31G(d)) = -2799.457249 au
E(SMD/M06-2X/def2-TZVP//SMD/M06-2X /6-31G(d)) = -2800.860791 au

C	-2.64229700	0.44457800	1.63181800
N	-2.38086900	-1.55557600	1.53743800
N	-2.53607500	-2.63940200	1.44907100
C	-4.01837700	0.66843700	1.63289700
C	-4.70489800	0.79886700	0.39812800
C	-4.71902600	0.78997400	2.85910800
C	-6.06049900	1.06725100	0.39970000
H	-4.14897600	0.69741900	-0.52858200
C	-6.07468000	1.05932600	2.84394400
H	-4.17410200	0.67708300	3.79077200
C	-6.73708100	1.19514700	1.61847900
H	-6.59786600	1.17966100	-0.53540300
H	-6.62335900	1.16512000	3.77319900
C	-1.36833000	0.81645900	1.45732600
O	-0.30454800	0.11257300	1.44544500
B	0.58102200	-0.07730500	0.18448000
C	0.98432300	1.37003300	-0.45940400
C	1.46455300	2.37339700	0.37857800
C	1.02171300	1.67098600	-1.81525100
C	1.91390400	3.60430600	-0.07359300
C	1.46875200	2.88943800	-2.31274800
C	1.91623500	3.86330200	-1.43611300
C	-0.34461300	-1.02715100	-0.77619900
C	-0.21643100	-2.40444300	-0.91700300
C	-1.48262000	-0.49158600	-1.37528100
C	-1.14097300	-3.19456300	-1.59314600
C	-2.43411600	-1.23835100	-2.05103400
C	-2.26288600	-2.61027600	-2.15815300
C	1.97481500	-0.74451000	0.68776000
C	2.87209600	-1.18156600	-0.28269400
C	2.44220500	-0.81079500	1.99403500
C	4.13279400	-1.67914700	-0.00432500
C	3.70428900	-1.30249300	2.31813800
C	4.55323400	-1.73921100	1.31733500
F	0.61560900	0.77967400	-2.73208200
F	1.47266400	3.12699500	-3.62690800
F	2.34748800	5.03683000	-1.89624300
F	2.35208000	4.53202700	0.78099900
F	1.50983900	2.16536300	1.70402100

F	2.50389000	-1.13373500	-1.57508900
F	4.94293100	-2.09383700	-0.98137000
F	5.76332600	-2.21059400	1.61684500
F	4.10293700	-1.34951400	3.59249300
F	1.69992200	-0.40063700	3.03127000
F	-1.71659100	0.83384200	-1.29848300
F	-3.51122200	-0.65225900	-2.58132700
F	-3.16066700	-3.35378100	-2.79933600
F	-0.96235500	-4.51258800	-1.68994700
F	0.81450300	-3.06679600	-0.37332100
H	-7.80256700	1.40529900	1.61289400
F	-1.23610800	2.14365000	1.36200900

1a•B(C_6F_5)$_3$ (R = H, R′ = Me)
E(SMD/M06-2X/6-31G(d)) = -2630.281511 au
H(SMD/M06-2X/6-31G(d)) = -2629.939184 au
G(SMD/M06-2X/6-31G(d)) = -2630.051044 au
E(SMD/M06-2X/def2-TZVP//SMD/M06-2X /6-31G(d)) = -2631.408762 au

C	2.54098600	0.51499400	-1.87177000
C	3.87129200	0.21428700	-1.91838600
C	4.72594800	0.56939100	-0.82870200
C	4.40897700	-0.45657100	-3.06082700
C	6.06588500	0.25638500	-0.88772900
H	4.30425800	1.09774400	0.02021800
C	5.75054700	-0.76754600	-3.09688700
H	3.74160400	-0.71363500	-3.87675800
C	6.56784000	-0.41143700	-2.01449900
H	6.73078100	0.52125300	-0.07361400
H	6.17594000	-1.28059200	-3.95183100
C	1.26441600	0.81697300	-1.93310800
O	0.32372300	-0.06518900	-1.60780000
B	-0.39204300	-0.07784600	-0.27953700
C	-1.28692300	1.28917700	-0.05412500
C	-2.08945300	1.74159600	-1.09971400
C	-1.43931800	1.99157000	1.13597400
C	-2.94087700	2.83237000	-1.00736700
C	-2.28527700	3.08603700	1.27676800
C	-3.03997800	3.51195000	0.19710200
C	0.77108200	-0.30884300	0.85912600
C	1.09349300	-1.52655700	1.44884600
C	1.62781700	0.73358600	1.19985900
C	2.15982600	-1.70065900	2.32458700
C	2.70432900	0.60731700	2.06277800
C	2.98119700	-0.62805400	2.62637500
C	-1.50721600	-1.27199000	-0.35038900
C	-2.19924400	-1.59951100	0.81256100
C	-1.94906000	-1.92369200	-1.49503700
C	-3.23053300	-2.52040800	0.87120400
C	-2.98484500	-2.85541200	-1.48209000
C	-3.62851200	-3.15736500	-0.29593300
F	-0.76171200	1.64164500	2.24066400

F	-2.37958100	3.72678100	2.44536000
F	-3.85479000	4.56024500	0.31396200
F	-3.67279900	3.22335800	-2.05440300
F	-2.06554600	1.10669500	-2.28456200
F	-1.85559300	-0.99966800	1.96696300
F	-3.84499300	-2.79714600	2.02499000
F	-4.62051800	-4.04852600	-0.27195300
F	-3.36515900	-3.45764800	-2.61364200
F	-1.40321500	-1.69368200	-2.69723300
F	1.43425700	1.95989200	0.67801900
F	3.51137700	1.64845400	2.30128200
F	4.02220700	-0.78063300	3.44335000
F	2.41163300	-2.89865900	2.85831900
F	0.37729900	-2.63318000	1.19595600
H	7.62508200	-0.65789800	-2.05106500
C	0.88855000	2.18930700	-2.43210500
H	0.12067200	2.07097700	-3.20139200
H	0.48241700	2.77051900	-1.59948600
H	1.74817900	2.71964700	-2.84869900

1a•B(C_6F_5)$_3$ (R = H, R′ = H)
E(SMD/M06-2X/6-31G(d)) = -2590.977729 au
H(SMD/M06-2X/6-31G(d)) = -2590.665301 au
G(SMD/M06-2X/6-31G(d)) = -2590.774321 au
E(SMD/M06-2X/def2-TZVP//SMD/M06-2X /6-31G(d)) = -2592.092331 au

C	-2.68322900	1.06254900	1.68313700
C	-4.02815200	0.97454100	1.86838900
C	-4.85782700	0.63258100	0.75239400
C	-4.60938400	1.20693500	3.15408800
C	-6.21915400	0.52752800	0.92929100
H	-4.39036800	0.47244700	-0.21471500
C	-5.97281000	1.09592000	3.30833100
H	-3.95876600	1.46385500	3.98344500
C	-6.76572700	0.75769000	2.20073300
H	-6.86673300	0.27116200	0.09868700
H	-6.43605800	1.26601800	4.27351200
C	-1.42196200	1.18875600	1.35837500
O	-0.50300700	0.24792900	1.46600100
B	0.36924100	-0.05802400	0.26971400
C	0.90703400	1.32250600	-0.44639000
C	1.43791000	2.33199600	0.35297400
C	1.01455900	1.54784200	-1.81440000
C	2.00105400	3.49865900	-0.14350400
C	1.57623800	2.69754200	-2.35622000
C	2.07131000	3.68119800	-1.51559900
C	-0.58586600	-0.98974000	-0.69557300
C	-0.54665900	-2.37743100	-0.77081400
C	-1.65419400	-0.41395800	-1.37860800
C	-1.47791400	-3.13857400	-1.47011300
C	-2.61474200	-1.13001600	-2.07429400
C	-2.52681200	-2.51254600	-2.12074100

C	1.71823400	-0.79919400	0.81038200
C	2.59729100	-1.33442600	-0.12689600
C	2.16482400	-0.84292300	2.12477000
C	3.81842700	-1.90289600	0.19012300
C	3.38708100	-1.40387600	2.48815100
C	4.21773500	-1.93669500	1.51917500
F	0.57351600	0.64373400	-2.70136400
F	1.64658500	2.86185100	-3.67965400
F	2.60958300	4.79033700	-2.02067900
F	2.48103000	4.43435100	0.67955200
F	1.41770400	2.20012200	1.69137300
F	2.25358300	-1.31347600	-1.42724500
F	4.61250500	-2.40935000	-0.75745600
F	5.39067300	-2.47477900	1.85544400
F	3.76611400	-1.42477200	3.76995200
F	1.44059600	-0.34150700	3.13473200
F	-1.80492500	0.92544300	-1.38758000
F	-3.63149600	-0.50156100	-2.67485900
F	-3.43870100	-3.22653300	-2.77978400
F	-1.37905300	-4.47019700	-1.50103300
F	0.41092300	-3.08153900	-0.14603600
H	-7.84045700	0.67168500	2.33287600
H	-1.15109900	2.18858500	0.98839300

1a•B(C_6F_5)$_3$ (R = H, R′ = Ph)
E(SMD/M06-2X/6-31G(d)) = -2821.950769 au
H(SMD/M06-2X/6-31G(d)) = -2821.551927 au
G(SMD/M06-2X/6-31G(d)) = -2821.671464 au
E(SMD/M06-2X/def2-TZVP//SMD/M06-2X /6-31G(d)) = -2823.142621 au

C	-2.47433200	0.61940300	1.46578600
C	-3.80486800	0.35853400	1.62579300
C	-4.63733600	0.17706800	0.47836800
C	-4.36077300	0.26025200	2.93840300
C	-5.97584700	-0.09927500	0.64852700
H	-4.19692300	0.26504700	-0.50967600
C	-5.70255000	-0.01503000	3.08682800
H	-3.70931300	0.40271000	3.79426500
C	-6.49893600	-0.19439400	1.94649900
H	-6.62282700	-0.24132600	-0.20959800
H	-6.14318100	-0.09508400	4.07400100
C	-1.21425100	0.95392300	1.33099100
O	-0.19691600	0.10114800	1.40970300
B	0.51640600	-0.41054600	0.17088600
C	1.08666100	0.84660800	-0.72943500
C	1.82276800	1.83060300	-0.07068100
C	0.96173300	1.04454400	-2.09950500
C	2.28639800	2.98883200	-0.67502000
C	1.42727400	2.18024900	-2.75310100
C	2.08397600	3.16549000	-2.03535600
C	-0.58201100	-1.36843500	-0.60177600
C	-0.63691700	-2.75603800	-0.49571700

C	-1.64642100	-0.81900700	-1.31196700
C	-1.63762000	-3.53814700	-1.06225200
C	-2.66854000	-1.55589100	-1.89006200
C	-2.66887300	-2.93490300	-1.76087400
C	1.84813300	-1.20925100	0.67872200
C	2.59089200	-1.91931500	-0.26021900
C	2.40878300	-1.15786500	1.94870600
C	3.78407600	-2.56327600	0.01783700
C	3.60791000	-1.78850900	2.27178500
C	4.29920200	-2.49538500	1.30456000
F	0.36638400	0.13264800	-2.88458900
F	1.25530800	2.32372800	-4.07079400
F	2.53073400	4.26203800	-2.64760200
F	2.94227300	3.91558000	0.02922000
F	2.10803800	1.68925700	1.23397400
F	2.13315300	-2.00842100	-1.52238500
F	4.44147500	-3.23933700	-0.92900300
F	5.44916500	-3.10219900	1.60209100
F	4.10031300	-1.70881800	3.51243900
F	1.82867900	-0.48440000	2.95192900
F	-1.74085800	0.51641300	-1.47330400
F	-3.66619000	-0.94161000	-2.53740200
F	-3.64378900	-3.66581000	-2.29818300
F	-1.62096100	-4.86576600	-0.92103800
F	0.29508000	-3.44068000	0.18570600
H	-7.55540700	-0.41327900	2.07230300
C	-0.97102300	2.42532300	1.19096200
C	-0.13140900	3.04144800	2.12571300
C	-1.50567900	3.15262800	0.12495100
C	0.20121700	4.38089300	1.96653000
H	0.28005000	2.45804900	2.94336900
C	-1.17128800	4.49677000	-0.02338700
H	-2.15244400	2.66059300	-0.59406900
C	-0.31443300	5.10695600	0.89037200
H	0.86657800	4.85984000	2.67804300
H	-1.57146900	5.06268500	-0.85873500
H	-0.04647800	6.15188100	0.76662600

1a•B(C_6F_5)$_3$ (R = H, R′ = OH)
E(SMD/M06-2X/6-31G(d)) = -2666.176984 au
H(SMD/M06-2X/6-31G(d)) = -2665.858725 au
G(SMD/M06-2X/6-31G(d)) = -2665.970858 au
E(SMD/M06-2X/def2-TZVP//SMD/M06-2X /6-31G(d)) = -2667.328915 au

C	2.53138300	0.66614200	-1.70963700
C	3.81119100	0.23877500	-1.90343900
C	4.79625400	0.47894000	-0.89132300
C	4.18096800	-0.44927600	-3.10485300
C	6.08883800	0.04327800	-1.07837500
H	4.50016900	1.01583200	0.00494200
C	5.47727700	-0.88115200	-3.27209400
H	3.41988300	-0.62131200	-3.85905200

C	6.41947000	-0.63432400	-2.26155200
H	6.84731700	0.21701200	-0.32375000
H	5.77373300	-1.40808700	-4.17198900
C	1.21992600	0.85707800	-1.87141200
O	0.39969800	-0.09189600	-1.51804500
B	-0.38997600	-0.06762900	-0.20291200
C	-1.33049500	1.27121900	-0.10595900
C	-2.09968400	1.63037600	-1.21035300
C	-1.54794800	2.03890600	1.03126400
C	-2.98446300	2.69767200	-1.21804300
C	-2.42745500	3.11494200	1.06919800
C	-3.14890800	3.44890900	-0.06397200
C	0.75908500	-0.18593000	0.96116800
C	1.13336000	-1.36046200	1.60453900
C	1.57460600	0.90776400	1.24183700
C	2.21913400	-1.45140600	2.46879300
C	2.67502400	0.85983600	2.08231000
C	3.00515800	-0.33603300	2.70050900
C	-1.43579900	-1.31749400	-0.25730000
C	-2.15217600	-1.61905000	0.89770800
C	-1.79790500	-2.05175300	-1.37943000
C	-3.13452600	-2.59089200	0.97105200
C	-2.78190000	-3.03747400	-1.35055500
C	-3.45296600	-3.31014600	-0.17247600
F	-0.90376700	1.77566500	2.17911600
F	-2.58610700	3.82479500	2.19037200
F	-3.99730400	4.47735500	-0.04485300
F	-3.68674500	3.00004400	-2.31413600
F	-2.01222400	0.91771900	-2.34387500
F	-1.88150000	-0.94051200	2.02710000
F	-3.77684300	-2.83939600	2.11563500
F	-4.39713900	-4.25092700	-0.13436300
F	-3.08608500	-3.71978700	-2.45903900
F	-1.21807500	-1.85496300	-2.57142800
F	1.31709200	2.09794300	0.67075000
F	3.45225700	1.93516700	2.25412700
F	4.06690200	-0.41038000	3.50176800
F	2.52396500	-2.61175200	3.05532000
F	0.45819800	-2.50447400	1.41364400
H	7.44033200	-0.97849600	-2.40097000
O	0.67182800	1.99462100	-2.31010100
H	1.35789900	2.65223900	-2.53631600

1a•B(C_6F_5)$_3$ (R = H, R′ = F)
E(SMD/M06-2X/6-31G(d)) = -2690.182299 au
H(SMD/M06-2X/6-31G(d)) = -2689.876411 au
G(SMD/M06-2X/6-31G(d)) = -2689.987519 au
E(SMD/M06-2X/def2-TZVP//SMD/M06-2X /6-31G(d)) = -2691.341503 au

C	2.56099900	0.72302700	-1.79004500
C	3.88190600	0.45634900	-1.90865100
C	4.74734000	0.62048500	-0.77357700

C	4.42323800	0.02287500	-3.16668500
C	6.09058000	0.35968800	-0.90319100
H	4.31994400	0.96516700	0.16269800
C	5.76857500	-0.23927700	-3.27039000
H	3.74946200	-0.09233600	-4.00931300
C	6.59015200	-0.07004800	-2.14376600
H	6.76312400	0.48197700	-0.06205600
H	6.19825400	-0.57131700	-4.20838200
C	1.26145400	0.94877200	-1.74853100
O	0.37076500	0.03875500	-1.55805000
B	-0.41416400	-0.08986200	-0.23561900
C	-1.15385500	1.32499700	0.13306100
C	-1.84917600	2.00432700	-0.86463900
C	-1.27789000	1.86995800	1.40548700
C	-2.57828100	3.16258800	-0.64375100
C	-2.00257000	3.02548000	1.67308400
C	-2.65551900	3.67800300	0.64118200
C	0.70827500	-0.57830700	0.85275000
C	0.90495400	-1.88884300	1.27476200
C	1.65711700	0.32432900	1.32539500
C	1.94553300	-2.27947700	2.11073200
C	2.71447000	-0.02089100	2.15126400
C	2.86543900	-1.34095100	2.54556900
C	-1.61933200	-1.15019600	-0.50809200
C	-2.36986300	-1.57786000	0.58366500
C	-2.08005200	-1.58639900	-1.74385300
C	-3.47689900	-2.40244100	0.48924100
C	-3.19111100	-2.41469600	-1.88441700
C	-3.89254000	-2.82594400	-0.76562300
F	-0.68807800	1.29537400	2.46497800
F	-2.07710800	3.50863700	2.91614600
F	-3.35379300	4.78758100	0.87992300
F	-3.21413500	3.77537300	-1.64584500
F	-1.83928000	1.53344800	-2.12185900
F	-2.00597300	-1.18286500	1.81663700
F	-4.14645500	-2.78776300	1.57864900
F	-4.95723500	-3.61853900	-0.88914000
F	-3.58765500	-2.81088700	-3.09759800
F	-1.47835600	-1.23342400	-2.88784700
F	1.58026400	1.62295000	0.97864400
F	3.61054700	0.89841800	2.52831800
F	3.88027600	-1.69967300	3.32927100
F	2.07639900	-3.55595700	2.47839600
F	0.08544900	-2.87586100	0.88292300
H	7.65250800	-0.27776600	-2.23564900
F	0.84666000	2.20544100	-1.92612000

$B(C_6F_5)_3$

E(SMD/M06-2X/6-31G(d)) = -2207.513266 au

H(SMD/M06-2X/6-31G(d)) = -2207.326627 au

G(SMD/M06-2X/6-31G(d)) = -2207.414735 au

E(SMD/M06-2X/def2-TZVP//SMD/M06-2X /6-31G(d)) = -2208.482503 au

B	0.00022400	0.00013800	0.00117600
C	1.51100500	0.42357000	0.00020400
C	2.49865200	-0.33137800	-0.63852900
C	3.83560000	0.03076200	-0.65602500
C	4.23440500	1.18579000	0.00084100
C	3.29358900	1.96634700	0.65674100
C	1.96286200	1.58193600	0.63851900
C	-0.38891300	-1.51999700	0.00055200
C	-1.54011300	-1.99696000	-0.63277000
C	-1.89507800	-3.33574200	-0.65045700
C	-1.09027600	-4.25982100	-0.00014900
C	0.05938100	-3.83647500	0.65059800
C	0.39151800	-2.49178300	0.63321800
C	-1.12134400	1.09752000	0.00190500
C	-2.35131900	0.90899200	0.63822900
C	-3.35059000	1.86810000	0.65326600
C	-3.14443800	3.07305400	-0.00245100
C	-1.94295000	3.30694600	-0.65518800
C	-0.96032100	2.33080900	-0.63567900
F	2.17884900	-1.45500500	-1.28563500
F	4.73593600	-0.71739200	-1.29002500
F	5.51095300	1.54268700	0.00158700
F	3.67431300	3.07313700	1.29099400
F	1.10607500	2.37675200	1.28491900
F	-2.35710100	-1.15793500	-1.27471900
F	-2.99655900	-3.74037100	-1.27916800
F	-1.41908100	-5.54389200	-0.00038400
F	0.83049000	-4.72101100	1.27934700
F	1.51134000	-2.14861100	1.27487400
F	0.17379600	2.61662100	-1.28056800
F	-1.74505600	4.46100100	-1.28875100
F	-4.09302500	3.99883900	-0.00497500
F	-4.50108600	1.64401900	1.28457700
F	-2.61108300	-0.23003000	1.28539100

N_2

E(SMD/M06-2X/6-31G(d)) = -109.482264 au
H(SMD/M06-2X/6-31G(d)) = -109.473155 au
G(SMD/M06-2X/6-31G(d)) = -109.494898 au
E(SMD/M06-2X/def2-TZVP//SMD/M06-2X /6-31G(d)) = -109.5302842 au

N	0.00000000	0.00000000	0.54917900
N	0.00000000	0.00000000	-0.54917900

1•$B(C_6F_5)_3$ (R = NMe_2, R′ = OMe)

E(SMD/M06-2X/6-31G(d)) = -2948.895071 au
H(SMD/M06-2X/6-31G(d)) = -2948.454843 au
G(SMD/M06-2X/6-31G(d)) = -2948.582377 au
E(SMD/M06-2X/def2-TZVP//SMD/M06-2X /6-31G(d)) = -2950.147847 au

C	-2.14755700	0.24341800	-1.19991400
N	-1.78877000	1.53134000	-1.38566100
N	-1.55641000	2.61228000	-1.52717800

C	-3.60737900	0.00374600	-1.10751600
C	-4.28430600	0.26056900	0.09100800
C	-4.34064800	-0.45210600	-2.20766300
C	-5.64828400	0.05665100	0.19932100
H	-3.72530100	0.61554100	0.95405400
C	-5.70501800	-0.66943400	-2.11486100
H	-3.83154100	-0.64719200	-3.14771500
C	-6.39961500	-0.41970700	-0.90479300
H	-6.13529600	0.26568100	1.14388000
H	-6.23738600	-1.03016000	-2.98635300
C	-1.01586900	-0.58772800	-1.04065500
O	0.14827000	-0.08566800	-1.24213000
B	1.24147800	0.08726500	-0.14766900
C	1.48424800	-1.30239300	0.67197000
C	1.71124500	-2.47256900	-0.04860100
C	1.60092200	-1.42818000	2.04965500
C	1.99156300	-3.69516600	0.53984100
C	1.88708600	-2.63339700	2.68183000
C	2.08202000	-3.77409800	1.92239300
C	0.62759800	1.32283900	0.74110400
C	1.01870700	2.65509900	0.66558900
C	-0.52367700	1.10517000	1.49562200
C	0.32458700	3.69267600	1.28088500
C	-1.25493200	2.10536300	2.11460500
C	-0.82587800	3.41974000	2.00152500
C	2.63653200	0.41125200	-0.91295100
C	3.72621900	0.79444900	-0.13649700
C	2.91116800	0.22026300	-2.26059200
C	4.99806000	1.00701400	-0.63820800
C	4.17604500	0.42226900	-2.80707400
C	5.22386200	0.81666800	-1.99472600
F	1.43441200	-0.37003400	2.85761800
F	1.97846700	-2.69804400	4.01284000
F	2.35640500	-4.93730300	2.51172300
F	2.18609900	-4.78838200	-0.20260700
F	1.65425700	-2.44423400	-1.38854900
F	3.54658800	0.98519000	1.18236100
F	6.00057500	1.38557600	0.15841400
F	6.43886800	1.01057300	-2.50719700
F	4.38583600	0.22991600	-4.11252900
F	1.96579500	-0.17757000	-3.12245700

F	-1.00799300	-0.14843700	1.62000700
F	-2.36969400	1.81985400	2.79302200
F	-1.50758800	4.40289100	2.58352600
F	0.75127400	4.95105500	1.16811300
F	2.09659900	3.02709700	-0.03890000
O	-1.05319900	-1.85083600	-0.73085500
C	-2.22920300	-2.51923200	-0.23343300
H	-2.64608400	-1.96496700	0.60749000
H	-2.96447300	-2.63705300	-1.02956000
H	-1.86586700	-3.49163800	0.09554600
N	-7.74389300	-0.63019700	-0.80562500
C	-8.50011500	-1.04537200	-1.97272700
H	-9.55055200	-1.13829000	-1.69867700
H	-8.41894000	-0.31503800	-2.78795300
H	-8.15651900	-2.01734300	-2.34626700
C	-8.43681500	-0.31895900	0.43111100
H	-9.49211500	-0.56489700	0.31689900
H	-8.03842100	-0.90477900	1.26776300
H	-8.35444800	0.74544600	0.68639500

1•B(C_6F_5)$_3$ (R = NH_2, R′ = OMe)
E(SMD/M06-2X/6-31G(d)) = -2870.322167 au
H(SMD/M06-2X/6-31G(d)) = -2869.942516 au
G(SMD/M06-2X/6-31G(d)) = -2870.06209 au
E(SMD/M06-2X/def2-TZVP//SMD/M06-2X /6-31G(d)) = -2871.552012 au

C	2.53237600	-0.15791000	-1.26532400
N	2.23085000	-1.46435800	-1.40467000
N	2.03852100	-2.55774600	-1.50717800
C	3.97582200	0.17679300	-1.29579900
C	4.78114700	-0.09800700	-0.18358800
C	4.55389300	0.75789100	-2.42951000
C	6.13098100	0.20878200	-0.19637600
H	4.33415100	-0.54501300	0.70175500
C	5.90171400	1.07955600	-2.44549500
H	3.93792000	0.96600800	-3.30002000
C	6.71470600	0.80800300	-1.32914000
H	6.74906400	-0.00327600	0.67144800
H	6.34400500	1.54031300	-3.32433200
C	1.37265100	0.62646200	-1.07076400
O	0.22577600	0.07942100	-1.24059800
O	1.37705400	1.88967500	-0.75723700
C	2.52151000	2.56827300	-0.20091300
H	2.97195100	1.96034400	0.58349300

H	3.24388700	2.79737400	-0.98425700
H	2.11184200	3.48618400	0.21837400
B	-0.85042600	-0.10352200	-0.12843200
C	-1.11666600	1.28791900	0.67888200
C	-1.38889500	2.44259700	-0.05096400
C	-1.21231100	1.42625200	2.05685200
C	-1.69438900	3.66337900	0.52837800
C	-1.52167300	2.63037200	2.68036000
C	-1.76333300	3.75573500	1.91150400
C	-0.18700200	-1.31290000	0.76049600
C	-0.53906700	-2.65680200	0.70315200
C	0.97519500	-1.05493300	1.48475500
C	0.20191900	-3.66800300	1.30787800
C	1.75452500	-2.02756000	2.08868300
C	1.36203600	-3.35505500	1.99634600
C	-2.24572600	-0.47343500	-0.87122300
C	-3.31348800	-0.87965100	-0.07633300
C	-2.54324600	-0.30316000	-2.21677300
C	-4.58512800	-1.13337500	-0.55911200
C	-3.80879300	-0.54654600	-2.74450300
C	-4.83387100	-0.96329100	-1.91433700
F	-1.00103800	0.38253200	2.87305300
F	-1.59187800	2.70802000	4.01187000
F	-2.06144200	4.91735700	2.49222700
F	-1.93241600	4.74199800	-0.22254800
F	-1.35103400	2.39958800	-1.39128300
F	-3.11070800	-1.05180300	1.24164300
F	-5.56590900	-1.53290200	0.25411300
F	-6.04914300	-1.19783500	-2.40866800
F	-4.04133800	-0.37296000	-4.04868100
F	-1.62093300	0.11449600	-3.09413000
F	1.42238300	0.21433000	1.58981700
F	2.88016300	-1.70305600	2.73011600
F	2.08899800	-4.31324200	2.56499100
F	-0.18864500	-4.93955400	1.21451700
F	-1.62190300	-3.06614700	0.02775600
N	8.04169000	1.17319100	-1.32355200
H	8.47631300	1.29660300	-2.23015300
H	8.63719600	0.69296000	-0.65960000

1•B(C_6F_5)$_3$ (R = OMe, R′ = OMe)
E(SMD/M06-2X/6-31G(d)) = -2929.462368 au
H(SMD/M06-2X/6-31G(d)) = -2929.064847 au

G(SMD/M06-2X/6-31G(d)) = -2929.187428 au
E(SMD/M06-2X/def2-TZVP//SMD/M06-2X /6-31G(d)) = -2930.712882 au

C	2.39715800	-0.26847500	-1.07398800
N	2.03603500	-1.54478000	-1.30704600
N	1.78402800	-2.61577400	-1.48609800
C	3.85694400	-0.00754800	-1.01921200
C	4.60065700	-0.43276200	0.09153700
C	4.49996600	0.65187500	-2.06542400
C	5.96089200	-0.19462300	0.15131600
H	4.09755400	-0.93673100	0.91334600
C	5.86748400	0.91035900	-2.00927100
H	3.92750100	0.97607400	-2.92979800
C	6.60246600	0.48157300	-0.89873200
H	6.55216500	-0.51334500	1.00347000
H	6.34604900	1.43288100	-2.82878500
C	1.27182700	0.56871000	-0.89338400
O	0.11095800	0.09998800	-1.16325800
O	1.32923200	1.80591600	-0.49404000
C	2.46532700	2.36518500	0.19651000
H	2.84220900	1.65668800	0.93380600
H	3.24179100	2.64140000	-0.51694500
H	2.07065800	3.25200000	0.69055800
B	-1.04546700	-0.07975900	-0.13117200
C	-1.28458100	1.27902000	0.73707600
C	-1.44207500	2.48583600	0.05983800
C	-1.46091200	1.34845800	2.11239300
C	-1.71367900	3.68992000	0.68870500
C	-1.74241300	2.53340800	2.78388500
C	-1.86809100	3.71134400	2.06783900
C	-0.50784600	-1.37237800	0.72490200
C	-0.93307300	-2.68792200	0.57613800
C	0.62023400	-1.22251400	1.52925200
C	-0.29086200	-3.77077100	1.16977000
C	1.30476600	-2.26913900	2.12382700
C	0.84149500	-3.56379300	1.93974600
C	-2.40526000	-0.32788300	-0.98178300
C	-3.54404700	-0.71610900	-0.28216000
C	-2.60143400	-0.07251900	-2.33257600
C	-4.79028200	-0.87846900	-0.86081100
C	-3.83820000	-0.22218200	-2.95491100
C	-4.93700600	-0.62625000	-2.21808100
F	-1.35925100	0.25196600	2.87869600
F	-1.89491400	2.54294200	4.11064200
F	-2.13599900	4.85576300	2.69521000
F	-1.84026100	4.81978900	-0.01214500
F	-1.31925900	2.51329400	-1.27577700
F	-3.44122700	-0.96703400	1.03467300
F	-5.84280200	-1.26671700	-0.13678900
F	-6.12567700	-0.77107700	-2.80315200
F	-3.97153900	0.02984500	-4.26018100

F	-1.60169400	0.33896600	-3.12406800
F	1.13164400	0.01086200	1.72902700
F	2.40769000	-2.04562500	2.84271400
F	1.47566400	-4.59132100	2.49806700
F	-0.74868900	-5.00974800	0.98747700
F	-1.99366000	-2.99733300	-0.18232300
O	7.92819100	0.67333500	-0.74643700
C	8.62629100	1.34310300	-1.78646300
H	8.24355100	2.35969300	-1.92721100
H	9.66710400	1.38770900	-1.46647700
H	8.55654200	0.78853000	-2.72850100

1•B(C_6F_5)$_3$ (R = Me, R′ = OMe)
E(SMD/M06-2X/6-31G(d)) = -2854.280338 au
H(SMD/M06-2X/6-31G(d)) = -2853.889169 au
G(SMD/M06-2X/6-31G(d)) = -2854.011386 au
E(SMD/M06-2X/def2-TZVP//SMD/M06-2X /6-31G(d)) = -2855.497495 au

C	2.68763200	-0.19226800	-1.00488900
N	2.38759500	-1.47255700	-1.26672500
N	2.16430700	-2.55000400	-1.45352100
C	4.09861900	0.22703300	-1.14528400
C	5.13103700	-0.53816000	-0.59320200
C	4.40317100	1.39427200	-1.84939200
C	6.45056100	-0.13554900	-0.74952700
H	4.89789200	-1.44507500	-0.04072000
C	5.72849100	1.79972100	-1.97696100
H	3.60581800	1.98033600	-2.29952300
C	6.77068200	1.04277800	-1.43525100
H	7.24805500	-0.73898700	-0.32368400
H	5.95715800	2.71180800	-2.52153200
C	1.53389100	0.57902300	-0.70806800
O	0.39145100	0.12054000	-1.03640000
O	1.58658500	1.75149700	-0.14365600
C	2.65043400	2.14018800	0.75231800
H	3.16108700	1.26274800	1.14833900
H	3.34603700	2.79116400	0.22296500
H	2.15263500	2.68432500	1.55503100
B	-0.83888800	-0.09947700	-0.09873000
C	-1.09307100	1.17732100	0.88035200
C	-1.17777000	2.44737700	0.31535100
C	-1.35261300	1.12050700	2.24289300
C	-1.45790500	3.59400400	1.04009600
C	-1.64518500	2.24453400	3.00811500
C	-1.69622800	3.48869700	2.40349800
C	-0.38298800	-1.48342700	0.64973400
C	-0.81919600	-2.76623400	0.33946300
C	0.69213800	-1.43810100	1.53481700
C	-0.23781200	-3.91706500	0.86387800
C	1.30883900	-2.55360300	2.07598900
C	0.83448700	-3.81199700	1.73332100
C	-2.14535700	-0.21915000	-1.05296100

C	-3.33547700	-0.65015300	-0.47457200
C	-2.24596300	0.19102500	-2.37585600
C	-4.54429500	-0.70789600	-1.14552500
C	-3.44172000	0.15057800	-3.08775200
C	-4.59534400	-0.30075800	-2.47179400
F	-1.32739500	-0.04670600	2.90337800
F	-1.87895600	2.13226400	4.31810100
F	-1.97197800	4.57627300	3.12172100
F	-1.51104400	4.78878900	0.44604500
F	-0.96794000	2.59573200	-1.00162200
F	-3.32540600	-1.04823900	0.80945000
F	-5.65102700	-1.14281100	-0.53856400
F	-5.74592300	-0.34245500	-3.14275600
F	-3.48315700	0.55165700	-4.36133400
F	-1.18586000	0.65907900	-3.04858500
F	1.21410900	-0.24070600	1.87659100
F	2.34467700	-2.43294400	2.90763500
F	1.40433200	-4.90427300	2.23579800
F	-0.70030600	-5.12117000	0.52636100
F	-1.83004700	-2.97184700	-0.51578100
C	8.20481300	1.48075900	-1.56914600
H	8.59384600	1.83132700	-0.60660400
H	8.83993800	0.65096200	-1.89410700
H	8.30444800	2.29588100	-2.29015100

1•B(C_6F_5)$_3$ (R = F, R′ = OMe)

E(SMD/M06-2X/6-31G(d)) = -2914.186525 au

H(SMD/M06-2X/6-31G(d)) = -2913.832366 au

G(SMD/M06-2X/6-31G(d)) = -2913.951272 au

E(SMD/M06-2X/def2-TZVP//SMD/M06-2X /6-31G(d)) = -2915.436825 au

C	2.54364300	-0.15083100	-1.25593800
N	2.25208700	-1.45635400	-1.39750500
N	2.05336700	-2.54798100	-1.50476900
C	3.98759600	0.19700800	-1.29812000
C	4.80822400	-0.14233000	-0.21697900
C	4.52420800	0.84598100	-2.41362600
C	6.16178400	0.16860800	-0.24316600
H	4.37763000	-0.63585800	0.65068100
C	5.87459700	1.17623100	-2.44442400
H	3.88252700	1.09857000	-3.25225000
C	6.66359100	0.82550900	-1.35847100
H	6.82260500	-0.07885500	0.58016600
H	6.31955400	1.68658500	-3.29142900
C	1.38101500	0.63051400	-1.05201600
O	0.23951300	0.07955400	-1.22471000
O	1.38544300	1.88896700	-0.72306400
C	2.52223700	2.55488300	-0.13560700
H	2.98965400	1.91228500	0.61011200
H	3.23245400	2.84222900	-0.91093400
H	2.10094400	3.44032500	0.33840500
B	-0.84882000	-0.10630600	-0.11802900

C	-1.10722200	1.27966900	0.69950700
C	-1.37710200	2.43998200	-0.02231200
C	-1.19613500	1.41011700	2.07878100
C	-1.67655800	3.65825800	0.56517400
C	-1.49931200	2.61162900	2.71035400
C	-1.74049300	3.74227900	1.94912100
C	-0.19374100	-1.32771600	0.75941100
C	-0.55609600	-2.66851300	0.69279200
C	0.97261300	-1.08419600	1.48225400
C	0.17913500	-3.68969200	1.28800300
C	1.74746700	-2.06715100	2.07486700
C	1.34414000	-3.39092600	1.97445900
C	-2.23935600	-0.46139900	-0.87552600
C	-3.31606500	-0.86951500	-0.09378900
C	-2.52442000	-0.27692600	-2.22197200
C	-4.58481700	-1.11181000	-0.58995300
C	-3.78646700	-0.50864400	-2.76287400
C	-4.82097900	-0.92749200	-1.94552200
F	-0.98352600	0.36153200	2.88817100
F	-1.56365900	2.68177500	4.04241000
F	-2.03245800	4.90116200	2.53791100
F	-1.91239000	4.74220500	-0.17839500
F	-1.33974200	2.40515700	-1.36292400
F	-3.12566100	-1.05604100	1.22389600
F	-5.57442200	-1.51360800	0.21114300
F	-6.03305600	-1.15056300	-2.45245000
F	-4.00652900	-0.32140000	-4.06720600
F	-1.59231600	0.14420700	-3.08725800
F	1.42985800	0.18125000	1.59477400
F	2.87971700	-1.75622300	2.71120200
F	2.06626200	-4.35876600	2.53219400
F	-0.22130000	-4.95720500	1.18626800
F	-1.64335900	-3.06486800	0.01743900
F	7.96724900	1.13552800	-1.38640700

1•B(C_6F_5)$_3$ (R = H, R′ = OMe)

E(SMD/M06-2X/6-31G(d)) = -2814.982365 au

H(SMD/M06-2X/6-31G(d)) = -2814.620677 au

G(SMD/M06-2X/6-31G(d)) = -2814.738177 au

E(SMD/M06-2X/def2-TZVP//SMD/M06-2X /6-31G(d)) = -2816.185699 au

C	2.80723100	0.01245700	-1.25144100
N	2.59659500	-1.30086500	-1.42183200
N	2.44758000	-2.40065500	-1.53696700
C	4.17472600	0.52617000	-1.48608200
C	5.28374200	-0.13585700	-0.95068400
C	4.34960600	1.67942600	-2.25741000
C	6.56309600	0.35499800	-1.19030200
H	5.14257600	-1.02677100	-0.34367500
C	5.63135000	2.17760700	-2.47189800
H	3.48589800	2.17969400	-2.68767800
C	6.73798800	1.51473600	-1.94411400

H	7.42289200	-0.16256100	-0.77699100
H	5.76456000	3.07705900	-3.06457600
C	1.61393500	0.70818000	-0.92699900
O	0.49560400	0.13869600	-1.14769600
O	1.60200900	1.91636000	-0.44120500
C	2.67217600	2.44429200	0.37298700
H	3.27780500	1.63725800	0.78441200
H	3.27862300	3.12203500	-0.22737900
H	2.16998300	2.98762500	1.17364800
B	-0.63807700	-0.12170100	-0.10275500
C	-0.92718000	1.18998600	0.81968500
C	-1.16767900	2.40928100	0.19112500
C	-1.07660900	1.20014500	2.19987000
C	-1.49396100	3.57268100	0.86839600
C	-1.40966400	2.34279700	2.91978000
C	-1.61830100	3.53620200	2.25040900
C	-0.01191500	-1.41359900	0.68633400
C	-0.36491200	-2.74402200	0.49274500
C	1.11780200	-1.22662100	1.48007900
C	0.34241400	-3.80855400	1.04406700
C	1.85877000	-2.25190000	2.04318700
C	1.46276700	-3.56309000	1.81976400
C	-1.99872600	-0.40674500	-0.93850700
C	-3.10052500	-0.88623000	-0.23654500
C	-2.23581000	-0.09846400	-2.27152400
C	-4.34924000	-1.08398300	-0.79871300
C	-3.47636300	-0.28093900	-2.87686500
C	-4.53740200	-0.77515700	-2.13906900
F	-0.89860600	0.08489900	2.92352700
F	-1.53243400	2.29610700	4.24863900
F	-1.93617900	4.64018700	2.92469900
F	-1.69777100	4.71751400	0.21205300
F	-1.07173900	2.48977700	-1.14480000
F	-2.95673600	-1.19167500	1.06467400
F	-5.36511400	-1.55950400	-0.07468400
F	-5.72956700	-0.95235300	-2.70753300
F	-3.65072200	0.02680800	-4.16500600
F	-1.27408900	0.40301700	-3.05847100
F	1.56388200	0.02774600	1.70612600
F	2.93922200	-1.99561900	2.78214100
F	2.15260600	-4.57170500	2.34615000
F	-0.04593500	-5.06457000	0.82181000
F	-1.41491800	-3.08386900	-0.26705500
H	7.73694700	1.90073000	-2.12126100

1•B(C_6F_5)$_3$ (R = Cl, R′ = OMe)
E(SMD/M06-2X/6-31G(d)) = -3274.552192 au
H(SMD/M06-2X/6-31G(d)) = -3274.199208 au
G(SMD/M06-2X/6-31G(d)) = -3274.32033 au
E(SMD/M06-2X/def2-TZVP//SMD/M06-2X /6-31G(d)) = -3275.78925 au

C	2.54802600	-0.32792200	-0.84189900

N	2.19700800	-1.57591200	-1.17881900
N	1.91801700	-2.62769400	-1.42704000
C	3.97626500	0.03677100	-0.92096400
C	4.96752400	-0.85273500	-0.49727100
C	4.33554500	1.28214700	-1.44577000
C	6.30922000	-0.50450600	-0.59575200
H	4.69498000	-1.82030500	-0.08424900
C	5.67419100	1.64770500	-1.52503600
H	3.56993300	1.96723600	-1.80019300
C	6.64633600	0.74674200	-1.10242600
H	7.08213100	-1.19247200	-0.27088400
H	5.95760100	2.61436700	-1.92691300
C	1.42021100	0.48135900	-0.53284900
O	0.27696300	0.10789300	-0.94380100
O	1.51069500	1.60171500	0.12400900
C	2.53156700	1.84288300	1.11826900
B	-1.02809700	-0.08200400	-0.10097300
C	-1.24974600	1.13959700	0.95255200
C	-1.22040000	2.44679700	0.47285800
C	-1.57847700	1.01175700	2.29513400
C	-1.46063900	3.56031300	1.26054000
C	-1.83436200	2.10125200	3.12135100
C	-1.77378600	3.38249300	2.60122400
C	-0.71948300	-1.54583500	0.56454600
C	-1.24149500	-2.76585400	0.15115500
C	0.30652500	-1.64550900	1.50174600
C	-0.78981100	-3.99091100	0.63387500
C	0.79350900	-2.83873800	2.00711400
C	0.23471800	-4.02940000	1.56403500
C	-2.27249000	-0.03577700	-1.14030500
C	-3.52742500	-0.40608500	-0.66653800
C	-2.25311400	0.46066100	-2.43694700
C	-4.68942900	-0.32884700	-1.41389400
C	-3.39812400	0.55685800	-3.22311600
C	-4.62083200	0.16050400	-2.71125200
F	-1.66113100	-0.19485600	2.87490100
F	-2.13980400	1.92036200	4.40845500
F	-2.01330000	4.43718800	3.37877600
F	-1.40211400	4.79224800	0.74904100
F	-0.92781600	2.66520700	-0.81848100
F	-3.63264300	-0.88177800	0.58637100
F	-5.86344600	-0.71149000	-0.90658900
F	-5.72352700	0.24989600	-3.45398400
F	-3.32445800	1.03721400	-4.46739500
F	-1.11831200	0.88542400	-3.00928400
F	0.90680800	-0.51861100	1.94242700
F	1.78513600	-2.85611200	2.89889900
F	0.67939300	-5.19353100	2.02985600
F	-1.33153800	-5.12875100	0.19862300
F	-2.21490000	-2.83411300	-0.76681400

H	3.27924300	2.51998800	0.70551300
H	2.00638500	2.31467700	1.94880900
H	2.98655400	0.90622200	1.44002900
Cl	8.32988000	1.19527500	-1.21490600

1•B(C_6F_5)$_3$ (R = CF_3, R′ = OMe)
E(SMD/M06-2X/6-31G(d)) = -3151.918829 au
H(SMD/M06-2X/6-31G(d)) = -3151.548585 au
G(SMD/M06-2X/6-31G(d)) = -3151.675371 au
E(SMD/M06-2X/def2-TZVP//SMD/M06-2X /6-31G(d)) = -3153.276646 au

C	2.18087200	-0.47184700	-0.72952300
N	1.77837400	-1.69055200	-1.11432900
N	1.45374200	-2.71752900	-1.40580500
C	3.62922500	-0.18495400	-0.73535400
C	4.55075700	-1.14778200	-0.31599700
C	4.07317100	1.06364200	-1.18846400
C	5.91195500	-0.86606600	-0.34781600
H	4.20848000	-2.11541000	0.04032000
C	5.43060600	1.35066900	-1.19841800
H	3.35793200	1.80058900	-1.54300600
C	6.34418600	0.38284100	-0.78182800
H	6.62878600	-1.61358700	-0.02656200
H	5.78010900	2.31765300	-1.54762900
C	1.08448600	0.38957300	-0.44552100
O	-0.05907400	0.08215300	-0.90620500
O	1.20501900	1.49132700	0.23688400
C	2.19056300	1.66482900	1.27999900
B	-1.40043200	-0.06611800	-0.11092600
C	-1.60354100	1.14078200	0.96271100
C	-1.49666700	2.45575600	0.51669600
C	-1.99118900	0.99745200	2.28799100
C	-1.71801500	3.56085700	1.32162800
C	-2.23020100	2.07834500	3.13034300
C	-2.09198800	3.36700300	2.64435000
C	-1.18066600	-1.55590500	0.53150400
C	-1.74071900	-2.74170000	0.07093600
C	-0.19570100	-1.72208600	1.50281200
C	-1.35976500	-3.99609700	0.53893600
C	0.22140300	-2.94694800	1.99503400
C	-0.37214200	-4.10118100	1.50343100
C	-2.60533700	0.05914500	-1.18907400
C	-3.89055500	-0.26023100	-0.76157400
C	-2.52156900	0.57806400	-2.47432200
C	-5.02342700	-0.11350100	-1.54229000
C	-3.63556800	0.74408900	-3.29280100
C	-4.89093300	0.39641500	-2.82669900
F	-2.15195800	-0.21701700	2.83380500
F	-2.59367200	1.88234500	4.40000800
F	-2.31489400	4.41357700	3.43761200
F	-1.58511300	4.80012200	0.84289900
F	-1.14547900	2.68972800	-0.75716200

F	-4.05674900	-0.75407200	0.47757100
F	-6.22958700	-0.44982300	-1.07970300
F	-5.96427800	0.55141400	-3.60077700
F	-3.50060000	1.24398100	-4.52412500
F	-1.35016900	0.95749700	-3.00322900
F	0.43740700	-0.63326300	1.99063500
F	1.17817400	-3.02831300	2.92055500
F	0.00410100	-5.29440000	1.95559800
F	-1.93426800	-5.09883400	0.05788100
F	-2.68328300	-2.74523400	-0.88101600
C	7.80480300	0.72932400	-0.79457200
F	8.10313400	1.64923600	0.13889900
F	8.17697900	1.25085800	-1.97465800
F	8.58429200	-0.33493300	-0.56347200
H	2.97445000	2.33072900	0.91965500
H	1.64704700	2.12659800	2.10443900
H	2.60236600	0.70398700	1.58831000

1•B(C_6F_5)$_3$ (R = CN, R′ = OMe)
E(SMD/M06-2X/6-31G(d)) = -2907.198252 au
H(SMD/M06-2X/6-31G(d)) = -2906.836308 au
G(SMD/M06-2X/6-31G(d)) = -2906.958267 au
E(SMD/M06-2X/def2-TZVP//SMD/M06-2X /6-31G(d)) = -2908.436227 au

C	2.63202700	-0.29306300	-0.87601000
N	2.29443400	-1.55101300	-1.18970200
N	2.02290600	-2.60753300	-1.42393200
C	4.05264100	0.08763300	-0.97763400
C	5.06032300	-0.81471500	-0.61966000
C	4.38484500	1.35914000	-1.46008800
C	6.39311100	-0.45098900	-0.74046700
H	4.80351700	-1.79888600	-0.23791000
C	5.71593200	1.73588400	-1.56272500
H	3.60349900	2.04818300	-1.76807300
C	6.71902000	0.82796300	-1.20553000
H	7.17904300	-1.14576700	-0.46550100
H	5.98026300	2.72010400	-1.93387900
C	1.49524700	0.50509500	-0.55898300
O	0.35543600	0.11120000	-0.95426300
O	1.57876800	1.62893800	0.09128600
C	2.59494600	1.88656400	1.08700600
H	3.09310700	0.96301800	1.38151400
H	3.30820000	2.60653200	0.68628900
H	2.05659200	2.31377400	1.93319500
B	-0.94634200	-0.08432700	-0.10147000
C	-1.16506200	1.13637900	0.95285100
C	-1.15047300	2.44339400	0.47184900

C	-1.47877700	1.00708400	2.29893000
C	-1.39019100	3.55576600	1.26123900
C	-1.73389300	2.09538800	3.12694400
C	-1.68802400	3.37663700	2.60529000
C	-0.62275900	-1.54543800	0.56151500
C	-1.14567200	-2.76866400	0.15896500
C	0.41409600	-1.63795400	1.48745600
C	-0.68515800	-3.99016900	0.64252700
C	0.90965300	-2.82722300	1.99351300
C	0.34911100	-4.02144700	1.56207600
C	-2.19681800	-0.04569200	-1.13269900
C	-3.44669400	-0.42043000	-0.64922700
C	-2.18828400	0.44791500	-2.43042800
C	-4.61403700	-0.35014800	-1.38883600
C	-3.33893700	0.53712900	-3.20904100
C	-4.55634300	0.13648200	-2.68780700
F	-1.54678800	-0.19956800	2.88028100
F	-2.02471000	1.91326400	4.41713000
F	-1.92677500	4.43001500	3.38459100
F	-1.34573200	4.78760900	0.74839100
F	-0.87249800	2.66240500	-0.82262100
F	-3.54069500	-0.89336600	0.60557200
F	-5.78278400	-0.73669200	-0.87266600
F	-5.66436100	0.21908800	-3.42319200
F	-3.27586200	1.01484100	-4.45483000
F	-1.05893000	0.87665500	-3.01058700
F	1.01637200	-0.50681900	1.91503300
F	1.91111000	-2.83769400	2.87423000
F	0.80201200	-5.18204800	2.02832100
F	-1.22787000	-5.13140600	0.21798200
F	-2.12851000	-2.84380600	-0.74808500
C	8.10058800	1.21185500	-1.32072300
N	9.21202100	1.52069300	-1.41239900

1•B(C_6F_5)$_3$ (R = NO_2, R′ = OMe)
E(SMD/M06-2X/6-31G(d)) = -3019.408439 au
H(SMD/M06-2X/6-31G(d)) = -3019.041354 au
G(SMD/M06-2X/6-31G(d)) = -3019.164564 au
E(SMD/M06-2X/def2-TZVP//SMD/M06-2X /6-31G(d)) = -3020.700948 au

C	2.43538300	-0.39742000	-0.74539700
N	2.05759300	-1.62437100	-1.12841300
N	1.74351400	-2.65515600	-1.41696700
C	3.86818600	-0.06146300	-0.79508300

C	4.84067200	-1.03391300	-0.53180200
C	4.24957200	1.24253100	-1.13850200
C	6.18707400	-0.70782500	-0.60309000
H	4.55014800	-2.04518300	-0.26210500
C	5.59360000	1.58206200	-1.19176500
H	3.49722100	1.98805300	-1.37847300
C	6.53633600	0.59787000	-0.92378700
H	6.95388700	-1.44567600	-0.40193900
H	5.90776900	2.58512000	-1.45335300
C	1.31670400	0.42661500	-0.42229000
O	0.18143100	0.09835800	-0.88148700
O	1.41757200	1.50735400	0.29464600
C	2.39246500	1.65840600	1.35231200
H	2.83781600	0.69740100	1.60916000
H	3.15244600	2.37068300	1.03278300
H	1.83017300	2.05448900	2.19808600
B	-1.16944900	-0.07306100	-0.10194400
C	-1.39027800	1.11353200	0.98925700
C	-1.29312700	2.43629700	0.56472800
C	-1.78278000	0.94508300	2.31003400
C	-1.52903200	3.52641200	1.38557600
C	-2.03682500	2.01030700	3.16785400
C	-1.90863000	3.30779600	2.70288800
C	-0.94043200	-1.57186700	0.51384200
C	-1.49138600	-2.75416700	0.03383100
C	0.04173800	-1.74578900	1.48650600
C	-1.10693400	-4.01259400	0.48818800
C	0.46130700	-2.97446300	1.96635200
C	-0.12411100	-4.12505000	1.45659100
C	-2.36213100	0.06031200	-1.19173700
C	-3.64968100	-0.27297600	-0.78241500
C	-2.26729100	0.59620800	-2.46910300
C	-4.77464400	-0.12373300	-1.57392500
C	-3.37296800	0.76478200	-3.29816800
C	-4.63107500	0.40302500	-2.85037300
F	-1.93311600	-0.27950100	2.83542500
F	-2.40482100	1.79099000	4.43225100
F	-2.14629400	4.33923700	3.51132900
F	-1.40426000	4.77417100	0.92749400
F	-0.93461000	2.69267500	-0.70295800
F	-3.82550800	-0.78394600	0.44835100

F	-5.98362400	-0.47333100	-1.12916300
F	-5.69664100	0.56059400	-3.63440800
F	-3.22769700	1.28081200	-4.52153300
F	-1.09223800	0.99042500	-2.97900200
F	0.66760700	-0.65943000	1.98925000
F	1.41320300	-3.06311800	2.89610100
F	0.25509800	-5.32204200	1.89585500
F	-1.67294400	-5.11181100	-0.01047100
F	-2.42934800	-2.74978900	-0.92244700
N	7.95910100	0.95073000	-0.98587600
O	8.24797500	2.10319600	-1.25557900
O	8.77337100	0.07218100	-0.76300900

1•B(C_6F_5)$_3$ (R = NMe_2, R′ = OMe), B←NMe_2
E(SMD/M06-2X/6-31G(d)) = -2948.884061 au
H(SMD/M06-2X/6-31G(d)) = -2948.442029 au
G(SMD/M06-2X/6-31G(d)) = -2948.564472 au
E(SMD/M06-2X/def2-TZVP//SMD/M06-2X /6-31G(d)) = -2950.131856 au

C	-5.35683400	0.06752000	-1.14399300
N	-6.20733800	-0.79411000	-1.65725500
N	-6.94183400	-1.52378000	-2.09890300
C	-3.92743300	-0.07292300	-1.47548000
C	-3.36000900	-1.33740900	-1.66215800
C	-3.10122400	1.05007100	-1.57794700
C	-1.99727900	-1.48399700	-1.88698000
H	-3.97947700	-2.22804800	-1.59680100
C	-1.73650900	0.90672800	-1.79030300
H	-3.51822900	2.04293900	-1.45714900
C	-1.17033900	-0.35992000	-1.91156400
H	-1.60375200	-2.48717200	-1.98494600
H	-1.11623000	1.79608000	-1.80006300
C	-5.96587400	0.92439600	-0.12137100
O	-7.14380300	0.89952800	0.17191900
O	-5.05830700	1.71386100	0.45841000
B	1.06229900	-0.18405200	-0.45954400
C	2.56690300	-0.86797700	-0.32340800
C	3.58135700	-0.94547700	-1.27569800
C	3.00413900	-1.21614400	0.96128200
C	4.87643200	-1.38118400	-1.01511700
C	4.28347600	-1.65072000	1.26924300
C	5.23772700	-1.73786400	0.26926200
C	-0.06832900	-0.88606700	0.51958000
C	-1.19571800	-0.21103300	0.99978200
C	-0.07600500	-2.24807500	0.81005900
C	-2.25879000	-0.82454800	1.64463600
C	-1.11278900	-2.90568000	1.46082500
C	-2.22121700	-2.19000300	1.87664900

C	1.38056800	1.39277700	-0.07618800
C	1.21335900	1.89765200	1.22098000
C	2.11993500	2.25300400	-0.89160600
C	1.59289400	3.17409500	1.61673200
C	2.50921200	3.53952300	-0.54353400
C	2.23163100	4.01615700	0.72422800
F	2.17394900	-1.12817500	2.01147600
F	4.60258500	-1.97230700	2.52382800
F	6.47264400	-2.15118600	0.54120600
F	5.77182200	-1.44144700	-2.00246000
F	3.37305800	-0.61487000	-2.56451400
F	0.70810900	1.15267300	2.21294600
F	1.36713900	3.58045700	2.86742700
F	2.60383400	5.23979900	1.08916900
F	3.18672300	4.29439600	-1.41060200
F	2.56792900	1.84420600	-2.08901900
F	0.94914200	-3.03911500	0.44059900
F	-1.04246200	-4.21917600	1.68697800
F	-3.22914800	-2.80032800	2.49514200
F	-3.31665300	-0.11050700	2.03558500
F	-1.33700100	1.11194900	0.82961400
C	-5.53717400	2.49053700	1.56068000
H	-6.32098000	3.17706300	1.23391800
H	-5.92603900	1.83557100	2.34349500
H	-4.67249800	3.04388300	1.92414800
N	0.30969500	-0.47472300	-2.03971600
C	0.66788300	0.39070100	-3.22025900
H	1.70995300	0.24550000	-3.47360000
H	0.03256500	0.08064800	-4.05294200
H	0.48745600	1.43660300	-2.99350700
C	0.67375800	-1.86788400	-2.45513400
H	0.49180500	-2.57346800	-1.64914700
H	0.08458800	-2.13984800	-3.33316300
H	1.72502200	-1.88314100	-2.71961100

1•B(C_6F_5)$_3$ (R = OMe, R′ = OMe), B←OMe
E(SMD/M06-2X/6-31G(d)) = -2929.455907 au
H(SMD/M06-2X/6-31G(d)) = -2929.058051 au
G(SMD/M06-2X/6-31G(d)) = -2929.181297 au
E(SMD/M06-2X/def2-TZVP//SMD/M06-2X /6-31G(d)) = -2930.704369 au

C	5.62084900	0.97977600	-0.82456600
N	6.04701100	2.21881400	-0.67672200
N	6.41834400	3.27249200	-0.55125400
C	4.16877200	0.77101800	-0.94780100
C	3.28055600	1.76354500	-0.50980400
C	3.64514700	-0.39575100	-1.52779700
C	1.90523200	1.60335700	-0.63766800
H	3.65620400	2.67724400	-0.05831200

C	2.27307200	-0.57176700	-1.64014500
H	4.30926900	-1.16828100	-1.89202800
C	1.42449200	0.42523700	-1.18089300
H	1.22823400	2.37914300	-0.30554800
H	1.86152500	-1.47353900	-2.07898700
C	6.73559600	0.02583200	-0.76989600
O	7.89798800	0.36318500	-0.67519700
O	6.32427200	-1.24361500	-0.81958200
B	-1.12197500	0.06868000	-0.21843200
C	-1.94616600	-1.28440100	-0.59979000
C	-1.57461200	-2.28838400	-1.48387900
C	-3.09585300	-1.54993700	0.13996000
C	-2.30398200	-3.46188500	-1.64913200
C	-3.85039800	-2.70250300	0.00988000
C	-3.44729800	-3.67140300	-0.89881600
C	-1.98774100	1.44812600	-0.33328300
C	-1.56452900	2.64681300	0.23926400
C	-3.16832900	1.55868100	-1.06713700
C	-2.24787700	3.84800900	0.12949400
C	-3.88776800	2.73929300	-1.20398800
C	-3.42680700	3.89447600	-0.59721500
C	-0.41105200	-0.24360300	1.20651400
C	-0.78172900	0.30234700	2.43353300
C	0.49823100	-1.29772800	1.29526800
C	-0.21508500	-0.08808400	3.64102400
C	1.09423900	-1.71256900	2.47537100
C	0.73548400	-1.09458100	3.66319700
F	-3.51373500	-0.64113700	1.03766400
F	-4.94756600	-2.89244800	0.74396800
F	-4.15418600	-4.79009700	-1.04518400
F	-1.90222000	-4.38807900	-2.52182900
F	-0.47529000	-2.17829900	-2.25073100
F	-1.74671300	1.22572400	2.52568100
F	-0.60020500	0.48487300	4.78227000
F	1.28127400	-1.47951500	4.81342600
F	1.97413000	-2.71450100	2.48159200
F	0.81046200	-1.99634800	0.19217100
F	-3.67627500	0.50442300	-1.72520700
F	-5.01001800	2.76704500	-1.92333800
F	-4.09914500	5.03479400	-0.71847500
F	-1.78118800	4.95134600	0.71458600

F	-0.42795600	2.69437200	0.95432100
C	7.36985600	-2.22256400	-0.75374500
H	8.05783300	-2.10022300	-1.59266100
H	7.91656900	-2.12885400	0.18656600
H	6.86863400	-3.18754400	-0.80863100
O	0.02949000	0.23927500	-1.35587900
C	-0.43014200	0.59512800	-2.69203600
H	-0.38707400	1.68047800	-2.79837300
H	0.22279600	0.10087000	-3.40969600
H	-1.45087600	0.22916300	-2.79278500

1′•BCl_3 (R = H, R′ = OMe)
E(SMD/M06-2X/6-31G(d)) = -2012.892679 au
H(SMD/M06-2X/6-31G(d)) = -2012.707705 au
G(SMD/M06-2X/6-31G(d)) = -2012.776251 au
E(SMD/M06-2X/def2-TZVP//SMD/M06-2X /6-31G(d)) = -2013.240995 au

C	-1.05143000	0.07877800	0.06247900
N	-0.43415100	1.97617900	-0.16688600
N	-0.19086200	3.02897200	0.02903400
C	-2.45219600	0.09614800	-0.03956700
C	-3.08326900	-0.31170800	-1.24038500
C	-3.23239100	0.48830200	1.07467300
C	-4.46521600	-0.35087600	-1.30803900
H	-2.46759400	-0.59879500	-2.08747400
C	-4.61233400	0.46530600	0.98745300
H	-2.72823700	0.80034400	1.98425600
C	-5.22147100	0.04300400	-0.19965300
H	-4.96009900	-0.67580000	-2.21675100
H	-5.22049100	0.76395400	1.83424900
C	0.11234800	-0.54564700	-0.26372300
O	1.15168600	0.08937200	-0.73457300
O	0.27643100	-1.83635200	-0.03680400
C	-0.76344300	-2.57659900	0.61875800
H	-1.03772100	-2.08191500	1.55546500
H	-1.63352000	-2.67332800	-0.03480100
H	-0.33421300	-3.55626600	0.81903700
B	2.48077100	0.03065900	-0.09770400
H	-6.30544400	0.02009800	-0.26131700
Cl	2.27631400	0.00855200	1.76321500
Cl	3.33808800	1.59444300	-0.62981500
Cl	3.43559900	-1.45782200	-0.66988000

1′•BH_3 (R = H, R′ = OMe)
E(SMD/M06-2X/6-31G(d)) = -634.0092548 au
H(SMD/M06-2X/6-31G(d)) = -633.804804 au
G(SMD/M06-2X/6-31G(d)) = -633.862718 au
E(SMD/M06-2X/def2-TZVP//SMD/M06-2X /6-31G(d)) = -634.2580511 au

C	0.38866400	0.33997000	0.60480900

N	0.78470900	2.20673000	0.06727200
N	0.82103700	3.29194700	0.24069400
C	-0.95587900	0.11599800	0.15571300
C	-1.24470800	-0.54324600	-1.06041700
C	-2.01808700	0.53231900	0.98583900
C	-2.55901000	-0.79774400	-1.41946000
H	-0.42664700	-0.85445200	-1.70562300
C	-3.33171800	0.29400200	0.61282100
H	-1.78244900	1.03706600	1.91809300
C	-3.59727800	-0.37386900	-0.58524700
H	-2.78223300	-1.31488000	-2.34671900
H	-4.14921200	0.61517400	1.24953500
C	1.56078100	-0.28034600	0.07831000
O	2.45309800	0.33246800	-0.56740300
O	1.79655100	-1.53199800	0.41642200
C	0.87369200	-2.25213800	1.25305400
H	0.60767600	-1.64978100	2.12609000
H	-0.01968300	-2.52606200	0.68884200
H	1.41035300	-3.14781600	1.56040300
B	3.86206300	-0.29056200	-0.88564800
H	-4.62647000	-0.56740900	-0.87286900
H	4.37264600	-0.62256400	0.16536000
H	4.45887500	0.61611100	-1.42645800
H	3.69068100	-1.23675100	-1.62884200

1′•BF_3 (R = H, R′ = OMe)
E(SMD/M06-2X/6-31G(d)) = -931.8710157 au
H(SMD/M06-2X/6-31G(d)) = -931.682587 au
G(SMD/M06-2X/6-31G(d)) = -931.747222 au
E(SMD/M06-2X/def2-TZVP//SMD/M06-2X /6-31G(d)) = -932.2713155 au

C	-0.50719100	0.36780100	0.63883100
N	-0.13709000	2.26157000	0.15515300
N	-0.16890500	3.34926500	0.31049700
C	-1.81998100	0.11600400	0.14050500
C	-2.03420200	-0.52869400	-1.09982500
C	-2.92818200	0.48692500	0.93317900
C	-3.32342300	-0.81540400	-1.51816800
H	-1.17766500	-0.80108400	-1.71146900
C	-4.21563400	0.21465800	0.50013600
H	-2.74723000	0.98193500	1.88261700
C	-4.40792000	-0.43875900	-0.72028600
H	-3.49236900	-1.32136600	-2.46277400
H	-5.06965600	0.49773900	1.10607500
C	0.72944000	-0.19328100	0.26564100
O	1.67725000	0.47038600	-0.26321400
O	0.98342700	-1.43702800	0.61575200
C	0.00633200	-2.21158500	1.33357300
H	-0.37748200	-1.63801700	2.18180300
H	-0.80759100	-2.50882900	0.66993800
H	0.54483400	-3.09044300	1.68266500

B	3.13642500	-0.05966000	-0.32931200
H	-5.41759200	-0.65809900	-1.05476700
F	3.54361800	-0.33180200	0.95933200
F	3.83538000	0.98773300	-0.88685000
F	3.14586200	-1.17729100	-1.13569400

1′•BPh_3 (R = H, R′ = OMe)
E(SMD/M06-2X/6-31G(d)) = -1326.932936 au
H(SMD/M06-2X/6-31G(d)) = -1326.465611 au
G(SMD/M06-2X/6-31G(d)) = -1326.557785 au
E(SMD/M06-2X/def2-TZVP//SMD/M06-2X /6-31G(d)) = -1327.428966 au

C	-2.31717900	0.36361000	0.18672500
N	-1.69566800	2.11967800	-0.52723900
N	-1.38997300	3.17498400	-0.49404000
C	-3.64986200	0.17448400	-0.27956700
C	-3.93248300	-0.66393700	-1.38222100
C	-4.71192300	0.79294800	0.41573900
C	-5.24601100	-0.89381300	-1.75925800
H	-3.11026100	-1.13000700	-1.91870400
C	-6.02136100	0.57983700	0.01704200
H	-4.47932400	1.43055200	1.26333300
C	-6.28364800	-0.26644800	-1.06414800
H	-5.46850300	-1.54801400	-2.59553100
H	-6.83987700	1.05606900	0.54601400
C	-1.12843200	-0.34790600	-0.04173700
O	-0.11174800	0.13805600	-0.62557500
O	-1.00117200	-1.54329800	0.50616300
C	-1.95396200	-2.00755000	1.47546600
H	-2.12063200	-1.23278800	2.22934400
H	-2.89434600	-2.28355100	0.99301900
H	-1.49482500	-2.88481300	1.92846000
B	1.37963800	-0.04448400	-0.08077900
H	-7.31147200	-0.44060300	-1.36854700
C	2.02830000	-1.33993000	-0.80038300
C	3.37976000	-1.65911000	-0.58240800
C	1.31998900	-2.17684300	-1.67300600
C	3.98912400	-2.75580300	-1.18760200
H	3.97252500	-1.02894500	0.08021500
C	1.91847000	-3.27547900	-2.29316000
H	0.27258200	-1.96707800	-1.87903600
C	3.25695100	-3.57251700	-2.05016400
H	5.03604200	-2.97274900	-0.99118600
H	1.33715200	-3.90079800	-2.96634900
H	3.72684900	-4.42734300	-2.52856900
C	1.26703300	-0.13119500	1.54013400
C	0.67892500	0.93828400	2.23840800
C	1.66561700	-1.23689300	2.30326800
C	0.48990100	0.90779100	3.61924100
H	0.36343300	1.82158800	1.68197500
C	1.49374200	-1.27818900	3.68812700

H	2.11276800	-2.09332700	1.80269300
C	0.90091000	-0.20582800	4.35180800
H	0.02895900	1.75226700	4.12533600
H	1.81766200	-2.15126900	4.24907900
H	0.76227100	-0.23528400	5.42907300
C	2.10287000	1.32771300	-0.56398600
C	3.20429700	1.85043300	0.13170300
C	1.69522800	2.02444400	-1.71241500
C	3.87142200	2.99878100	-0.29451400
H	3.54237200	1.35310900	1.03971500
C	2.34626700	3.17956600	-2.14526900
H	0.84257700	1.65577400	-2.27954000
C	3.44193600	3.67140000	-1.43716400
H	4.72105500	3.37431700	0.27019800
H	1.99964100	3.69685200	-3.03656800
H	3.95272700	4.57066100	-1.77023400

1a•BCl_3 (R = H, R′ = OMe)
E(SMD/M06-2X/6-31G(d)) = -1903.420534 au
H(SMD/M06-2X/6-31G(d)) = -1903.245078 au
G(SMD/M06-2X/6-31G(d)) = -1903.307661 au
E(SMD/M06-2X/def2-TZVP//SMD/M06-2X /6-31G(d)) = -1903.723597 au

C	1.05845000	0.64805200	-0.61364800
C	2.28274600	0.13224300	-0.32504200
C	3.32731900	0.16293500	-1.30629000
C	2.53314100	-0.44469100	0.96477500
C	4.56319200	-0.35956100	-1.00223000
H	3.11432400	0.60247300	-2.27529800
C	3.77516400	-0.96392500	1.24861900
H	1.72415700	-0.46249000	1.68865000
C	4.77837900	-0.91804900	0.26773300
H	5.36619200	-0.34596700	-1.73034800
H	3.98296300	-1.40697500	2.21595800
C	-0.23089500	0.97769000	-0.53093400
O	-1.11691400	0.11416500	-0.99427600
O	-0.68619800	2.15348000	-0.13186000
C	0.29181800	3.02385300	0.45138300
H	0.71831300	2.56088200	1.34626800
H	1.07964500	3.24475100	-0.27743500
H	-0.23806600	3.93691600	0.71508700
B	-2.14182400	-0.45515500	-0.11183700
H	5.75608200	-1.33121700	0.49922800
Cl	-1.44202600	-0.68851500	1.61669600
Cl	-2.60609500	-2.09862400	-0.84631000
Cl	-3.63690700	0.65684300	-0.03744600

1a•BH_3 (R = H, R′ = OMe)
E(SMD/M06-2X/6-31G(d)) = -524.5376283 au
H(SMD/M06-2X/6-31G(d)) = -524.343022 au
G(SMD/M06-2X/6-31G(d)) = -524.395153 au
E(SMD/M06-2X/def2-TZVP//SMD/M06-2X /6-31G(d)) = -524.7417497 au

C	-0.40006500	0.44190400	0.86645800
C	0.87644200	0.12462400	0.39943400
C	1.10781600	-0.62632500	-0.79362000
C	1.99629800	0.58140400	1.15342700
C	2.39564600	-0.89862700	-1.20459300
H	0.25652200	-0.98234400	-1.36836400
C	3.28303400	0.30408600	0.73536300
H	1.80364000	1.14901600	2.05831500
C	3.47464700	-0.43207100	-0.43952000
H	2.57937900	-1.46762600	-2.10931100
H	4.13835500	0.64934100	1.30545600
C	-1.61091500	0.00467200	0.26235200
O	-2.06377500	-1.13382200	0.56124000
O	-2.34579000	0.82413100	-0.45348300
C	-1.76210500	2.09469300	-0.79469600
H	-1.43703000	2.61156000	0.11328900
H	-0.92145700	1.95117700	-1.47743200
H	-2.55231100	2.65776100	-1.28713900
B	-3.53595700	-1.58512300	0.22112300
H	4.48700700	-0.64900200	-0.76838300
H	-4.29500400	-0.79277500	0.74243100
H	-3.61760000	-2.69364700	0.70450400
H	-3.64998500	-1.59647600	-0.98866600

1a•BF_3 (R = H, R′ = OMe)
E(SMD/M06-2X/6-31G(d)) = -822.3995429 au
H(SMD/M06-2X/6-31G(d)) = -822.220836 au
G(SMD/M06-2X/6-31G(d)) = -822.279425 au
E(SMD/M06-2X/def2-TZVP//SMD/M06-2X /6-31G(d)) = -822.7549344 au

C	0.56095600	0.55192300	0.84603700
C	1.79428600	0.09829200	0.39826100
C	1.97220100	-0.51108300	-0.88323300
C	2.92476800	0.25015500	1.25558400
C	3.21993800	-0.94302300	-1.27782000
H	1.11001600	-0.62965000	-1.53419600
C	4.16943100	-0.18937200	0.85284700
H	2.77245800	0.71485700	2.22455300
C	4.30924400	-0.78137000	-0.40859400
H	3.36462000	-1.40659200	-2.24737600
H	5.03254100	-0.08109200	1.49996500
C	-0.69076800	0.44523500	0.21561700
O	-1.38472500	-0.60536300	0.40302900
O	-1.23065300	1.46259500	-0.41212600
C	-0.39458100	2.61630600	-0.61773600
H	-0.01329200	2.97293100	0.34395700

H	0.42942600	2.36844800	-1.29049900
H	-1.03780800	3.36721300	-1.07153300
B	-2.90515300	-0.66832200	0.08076500
H	5.28972900	-1.12665200	-0.72411700
F	-3.52378400	0.32837600	0.80449200
F	-3.27757300	-1.92639800	0.49567400
F	-3.05328200	-0.50398800	-1.27989700

1a•BPh_3 (R = H, R′ = OMe)
E(SMD/M06-2X/6-31G(d)) = -1217.459414 au
H(SMD/M06-2X/6-31G(d)) = -1217.001898 au
G(SMD/M06-2X/6-31G(d)) = -1217.089912 au
E(SMD/M06-2X/def2-TZVP//SMD/M06-2X /6-31G(d)) = -1217.911774 au

C	2.44936800	-1.04230700	-0.57900900
C	3.63551500	-0.32155700	-0.45618800
C	3.80237400	0.74358600	0.48147200
C	4.72656400	-0.67085200	-1.30444400
C	5.00226700	1.41763500	0.56097800
H	2.96849900	1.01443200	1.12459100
C	5.92477400	0.01041800	-1.21903300
H	4.58416200	-1.48102400	-2.01249400
C	6.05549000	1.04783200	-0.28841700
H	5.13654900	2.22724100	1.26983700
H	6.75795800	-0.24995300	-1.86216600
C	1.24071600	-0.79761500	0.11282700
O	0.44789600	0.07536900	-0.34949300
O	0.85373400	-1.56377400	1.10484300
C	1.80690200	-2.52267600	1.59618400
H	2.15803900	-3.15249700	0.77321400
H	2.64547300	-2.00870200	2.07206400
H	1.26919900	-3.12208000	2.32809300
B	-1.12674400	0.11173300	-0.08685400
H	6.99851500	1.58287600	-0.22190300
C	-1.37738800	0.71140000	1.39856700
C	-2.69571200	0.89770200	1.85053700
C	-0.35695700	1.12660700	2.26407100
C	-2.98197600	1.44586200	3.09844800
H	-3.52200700	0.60601300	1.20269900
C	-0.62689400	1.68419600	3.51542400
H	0.68282000	1.02056800	1.95917200
C	-1.94300400	1.84276300	3.94102800
H	-4.01499200	1.56894600	3.41358500
H	0.19279900	1.99583400	4.15809300
H	-2.15864900	2.27436800	4.91441600
C	-1.67752400	-1.39503500	-0.34348800
C	-1.46094200	-1.99473500	-1.59647300
C	-2.34150800	-2.16953700	0.61719000
C	-1.87940100	-3.29365400	-1.87848500
H	-0.95108100	-1.42479000	-2.37269000
C	-2.77704800	-3.46788800	0.34669500

H	-2.51639200	-1.75355300	1.60708700
C	-2.54606600	-4.03647200	-0.90397800
H	-1.69200200	-3.72588500	-2.85820600
H	-3.29246200	-4.03812200	1.11553500
H	-2.88111200	-5.04762300	-1.11842600
C	-1.65668400	1.17031000	-1.19828700
C	-2.92732100	1.05617200	-1.78155800
C	-0.89453900	2.29243000	-1.56065800
C	-3.41817900	2.00939100	-2.67505900
H	-3.54807600	0.19558800	-1.53728200
C	-1.36711600	3.24718500	-2.45907900
H	0.09655200	2.41921600	-1.12860600
C	-2.63672100	3.10980600	-3.02035600
H	-4.40839800	1.88876300	-3.10719000
H	-0.74746000	4.10115200	-2.72144000
H	-3.01119700	3.85200100	-3.71996200

BCl_3

E(SMD/M06-2X/6-31G(d)) = -1405.461661 au
H(SMD/M06-2X/6-31G(d)) = -1405.448686 au
G(SMD/M06-2X/6-31G(d)) = -1405.483198 au
E(SMD/M06-2X/def2-TZVP//SMD/M06-2X /6-31G(d)) = -1405.575414 au

B	-0.00005100	-0.00008200	-0.00005700
Cl	1.47548100	-0.93214800	0.00000600
Cl	0.06982500	1.74343300	0.00000600
Cl	-1.54529100	-0.81126000	0.00000600

BH_3

E(SMD/M06-2X/6-31G(d)) = -26.58533473 au
H(SMD/M06-2X/6-31G(d)) = -26.554763 au
G(SMD/M06-2X/6-31G(d)) = -26.577832 au
E(SMD/M06-2X/def2-TZVP//SMD/M06-2X /6-31G(d)) = -26.59541085 au

B	0.00005600	0.00000000	0.00001400
H	-0.59587600	-1.03096100	-0.00002400
H	-0.59525000	1.03132200	-0.00002400
H	1.19084700	-0.00036100	-0.00002400

BF_3

E(SMD/M06-2X/6-31G(d)) = -324.4521062 au
H(SMD/M06-2X/6-31G(d)) = -324.435197 au
G(SMD/M06-2X/6-31G(d)) = -324.465829 au
E(SMD/M06-2X/def2-TZVP//SMD/M06-2X /6-31G(d)) = -324.6110106 au

B	-0.00002800	0.00011400	0.00020600
F	1.09783800	0.72382900	-0.00003800
F	-1.17591700	0.58860200	-0.00003800
F	0.07809500	-1.31249400	-0.00003800

BPh_3

E(SMD/M06-2X/6-31G(d)) = -719.5320178 au

H(SMD/M06-2X/6-31G(d)) = -719.235809 au
G(SMD/M06-2X/6-31G(d)) = -719.295326 au
E(SMD/M06-2X/def2-TZVP//SMD/M06-2X /6-31G(d)) = -719.790855 au

B	0.00010100	-0.00026400	-0.00000400
C	-0.61991400	1.44032100	0.00030800
C	0.02242600	2.51306200	-0.64525700
C	-1.84074300	1.71107300	0.64558700
C	-0.53187900	3.79027100	-0.66034600
H	0.96705100	2.33848100	-1.15466300
C	-2.38805600	2.99129100	0.65963300
H	-2.36305700	0.90510100	1.15537300
C	-1.73629900	4.03215200	-0.00070100
H	-0.02405000	4.59789200	-1.17957100
H	-3.32392000	3.17746800	1.17849200
H	-2.16619400	5.02982200	-0.00127500
C	-0.93737500	-1.25752100	-0.00022800
C	-0.56042900	-2.45106200	0.64285700
C	-2.18889900	-1.23649400	-0.64326000
C	-1.39595600	-3.56475100	0.65756700
H	0.39974500	-2.50120300	1.15062400
C	-3.01843900	-2.35464400	-0.65753400
H	-2.51074700	-0.33042400	-1.15094100
C	-2.62477400	-3.51944400	0.00011200
H	-1.08853100	-4.46909200	1.17466600
H	-3.97298200	-2.31773100	-1.17446200
H	-3.27432400	-4.39018700	0.00034400
C	1.55775400	-0.18329700	-0.00007500
C	2.16607400	-1.27567000	-0.64566700
C	2.40218100	0.73865400	0.64572600
C	3.54934700	-1.43419300	-0.65993200
H	1.54291400	-2.00641700	-1.15555300
C	3.78453600	0.57255200	0.66066400
H	1.96490200	1.59378300	1.15545500
C	4.36054800	-0.51223300	0.00051700
H	3.99519800	-2.27763600	-1.17912300
H	4.41334800	1.28987100	1.18007100
H	5.43951700	-0.63880300	0.00075800
